# Toward Public Health Resilience in the Eastern Mediterranean Region: Findings From the Seventh Eastern Mediterranean Public Health Network Regional Conference

**DOI:** 10.2196/36356

**Published:** 2023-03-09

**Authors:** The Eastern Mediterranean Public Health Network (EMPHNET)

**Keywords:** COVID-19, surveillance, public health, health system, conference, Eastern Mediterranean Region

## Abstract

The resilience of public health in the Eastern Mediterranean Region (EMR) varies from country to country, mostly based on the governmental and financial situation of the countries. With the theme of Towards Public Health Resilience in the EMR: Breaking Barriers, the seventh Eastern Mediterranean Public Health Network regional conference, held from November 14 to 18, 2021, was dedicated to exploring ways for achieving public health resilience. A total of 101 oral presentations and 13 poster presentations were presented on various public health topics. The conference included 6 keynote sessions, 10 roundtable sessions, and 5 preconference workshops. The preconference workshops were conducted on border health; the mobilization of Field Epidemiology Training Program (FETP) residents and graduates and rapid responders in EMR countries; continuous professional development for the public health workforce; brucellosis surveillance using the “One Health” approach; and strategies to integrate and use noncommunicable diseases data sources. The roundtable sessions included discussions on the following topics: the role of FETPs in responding to COVID-19, institutionalization of rapid response to public health emergencies, health systems resilience, integration of early warning and response with event-based and indicator-based surveillance, sustaining international health regulations, strengthening the “One Health” approach, the anticipated future of public health in the post COVID-19 era, supporting public health research capacity in a diverse region, and COVID-19 vaccines and routine immunization synergies and drawbacks. The keynote speaker sessions covered topics on essential public health functions and the universal health coverage challenge in the EMR, lessons from the US COVID-19 public health response, learning from COVID-19, reshaping public health after the pandemic era, COVID-19 resilient primary health care, and the cohesion of society during and after a pandemic. The conference sessions provided highly promising opportunities to explore ways to achieve such goals in the EMR and shed light on the latest scientific findings, important lessons learned, and discussions on the ways in which current barriers can be broken down through coordination and collaboration.

## Background

COVID-19 has caused an unprecedented global crisis, resulting in the loss of millions of lives, shock to public health systems, and economic and social disruption. The pandemic has disproportionately affected the most vulnerable populations [1]. It has challenged local, national, regional, and global capacities and exposed the limitations of many health systems, including those previously known for their high performance and resilience [2]. Health system resilience can be defined as the capacity of health actors, institutions, and populations to prepare for and effectively respond to crises; maintain core functions when a crisis hits; and, informed by lessons learned during the crisis, reorganize if conditions require it. Public health resilience is the ability to prepare for, manage (absorb, adapt, and transform), and learn from shocks. Shocks can predominantly affect the demand side of the health system (eg, an epidemic will increase health care needs) or its supply side (eg, an economic crisis will typically cause a reduction in available resources) or both and can be more or less severe.

Public health plays a pivotal role in building and strengthening resilience at the individual, community, and system levels. Public health resilience is needed more than ever before to recover from the extreme disruptions of today’s life and to adapt and prosper in the face of challenges and barriers, while maintaining the core functions of the health systems. During crises, resilient health systems can effectively adapt in response to evolving situations and reduce vulnerability across and beyond the systems. The resilience of public health in the Eastern Mediterranean Region (EMR) varies from country to country, mostly based on the governmental and financial situation of the countries. The various national strategies used to control viral transmission are widely debated. However, the relative success of these strategies depends largely on the structure, governance, and financing of the existing health system across all levels.

The seventh Eastern Mediterranean Public Health Network (EMPHNET) regional conference, held from November 14 to 18, 2021, was dedicated to exploring ways to achieve public health resilience. To this effect, EMPHNET adopted the theme of *Towards Public Health Resilience in the EMR: Breaking Barriers*. Conference sessions provided highly promising opportunities to explore ways to achieve such goals in the EMR and shed light on the latest scientific findings, important lessons learned, and discussions on the ways in which current barriers can be broken down through coordination and collaboration. The objectives of the conference were to engage public health experts and national, regional, and international entities in a discussion of public health challenges hindering the achievement of public health resilience in the EMR; share public health lessons and expertise; and present the accomplishments of public health professionals from the region. The conference consisted of oral and poster presentations, preconference workshops, roundtable discussions, and keynote speeches.

## Oral and Poster Abstract Sessions

A total of 101 oral presentations and 13 poster presentations were presented by FETP residents and graduates as well as public health specialists in the region. Their presentations offered opportunities to critically discuss new lessons learned from past years and explore new opportunities to become more resilient health systems. A total of 32 abstracts were in the area of COVID-19, 18 abstracts on surveillance, 7 abstracts on zoonotic diseases, 7 abstracts on vaccine-preventable diseases, 7 abstracts on noncommunicable diseases, 6 abstracts on vector-borne diseases, 6 abstracts on tuberculosis and HIV/AIDS, and 6 abstracts on maternal and child health. The remaining abstracts covered other areas of public health. All abstracts are made available in the abstract book (Multimedia Appendix 1).

## Preconference Workshops

There were 5 preconference workshops, facilitated by experts within their respective fields, highlighting public health concepts and topics relevant to the region. These topics were border health approaches to mitigate cross-border communicable disease spread; the mobilization of FETPs and rapid responders in EMR countries; continuous professional development for public health workforce; brucellosis surveillance, diagnosis, and control using the “One Health” approach; and identifying barriers and strategies to integrate and use noncommunicable diseases data sources.

### Border Health Approaches to Mitigate Cross-border Communicable Disease Spread

The COVID-19 pandemic has highlighted the fact that communicable diseases have no borders and has proved the vital role of border health strategies to limit the spread of such outbreaks globally. Border health measures aim to mitigate the international spread of communicable diseases through improved systems designed to detect, prevent, and respond to public health events [3]. In our increasingly connected world, border health strategies must remain innovative and resilient to effectively mitigate the cross-border spread of communicable diseases [4]. Therefore, it is important to strengthen cross-border public health surveillance, information sharing, and collaboration as part of a comprehensive border health system. This workshop aimed to understand population movement and connectivity to improve public health systems. The participants were able to identify border health factors and activities that a public health authority would consider when faced with a highly pathogenic outbreak in a neighboring country. Such factors include identifying resource needs, reviewing current public health emergency or pandemic plans and standard operating procedures (SOPs), enhancing surveillance, considering nonpharmaceutical interventions, and risk communication messaging. Participants were also able to explain how understanding population mobility and strengthening cross-border collaboration contribute to a comprehensive approach to border health and identify actions that a country should take to communicate a public health problem to its counterparts in a neighboring country. Challenges for cross-border collaborations were also identified, such as lack of up-to-date information from the borders, case admissions, lack of resources, language barriers, and political tension. At the end of the workshop, it was recommended that countries implement Public Health Corridors to harmonize their COVID-19 recovery road maps. Public Health Corridors is a strategy developed by the Collaborative Arrangement for the Prevention and Management of Public Health Events in Civil Aviation, which describes, from an aviation standpoint, how to handle essential flights in a manner that maintains safety and prevents the transmission of COVID-19, all while minimizing additional burdens. It is also important for countries to adopt multiple border health mechanisms as different layers of protection against the spread of internationally transmissible diseases. Moreover, there is a need to strengthen the detection, protection, and response capacities at the borders, including building the capacity of the officers responsible for public health activities at points of entry (POEs). Countries must ensure that they have relevant cross-border public health plans and SOPs developed and that relevant staff are familiar with and trained on these SOPs.

### Mobilization of FETPs and Rapid Responders in EMR Countries

The roles of public health rapid response teams (RRTs), FETPs, and public health emergency operations centers (PHEOCs) were witnessed during the COVID-19 pandemic. In the EMR, the FETP residents, graduates, and mentors have contributed substantially to the public health responses to the COVID-19 pandemic in their respective countries by being involved in case investigations, POE and arrival screening, isolation protocols, transferring cases, risk communication, and training on infection control [5]. RRTs, FETPs, and PHEOCs are critical assets within a country’s public health emergency response and manage disease outbreaks and other events [6]. Thus, coordinating the roles and responsibilities of these 3 elements is crucial for effective and efficient emergency response plans. This workshop aimed to introduce an operational framework that coordinates the roles of FETPs, RRTs, and PHEOCs. A framework that links FETPs, RRTs, and emergency operations centers (EOCs) was discussed and proposed during this workshop, as this will enhance the EMR emergency response capacity and strengthen the management and coordination among EMR countries. The operationalization of the proposed conceptual framework requires commitment from the EOCs, RRTs, and FETPs. The core element of an efficient coordinated response is preparedness. During the “nonemergency phase,” the development of SOPs is essential to indicate the role of each actor in the response. Having standardized training and capacity building is important to ensure that all actors share the same competencies and skills. During the emergency phase, maintaining good communication and sharing information are essential to ensure a coordinated response. Including FETPs in the development of SOPs and response strategies as well as their involvement at various levels of the health system is beneficial.

### Continuous Professional Development for Public Health Workforce

Continuing professional development (CPD) refers to all formal and informal activities that health workers engage in to maintain, update, develop, and improve their professional skills, knowledge, and attitudes [7]. CPD for public health workers is essential to ensure the best practices in a field that involves multiprofessional, multidisciplinary, and multiorganizational activities. FETPs are competency-based training and service programs in applied epidemiology and public health that help countries build public health system capacity. The COVID-19 pandemic revealed the need to equip FETP graduates with the additional competencies needed to manage the newly emerging threats. This regional workshop was conducted to propose a road map for CPD as part of FETP graduates’ career pathway. The workshop participants identified FETP graduates’ ongoing competency requirements and proposed a strategy for incorporating CPD activities to meet those requirements in the EMR in the coming years. This workshop devolved the participants’ skills to identify the needs and potential opportunities for FETP graduates to continue their professional education and to outline a work plan for FETP graduate support in the EMR. At the end of the workshop, it was recommended that CPD be institutionalized within the Public Health system and that the FETP career path should include CPD activities. Each ministry of health should appraise the public health workforce and have clear objectives and CPD plans to meet the needs of individuals and teams.

### Brucellosis Surveillance, Diagnosis, and Control Using the “One Health” Approach

Brucellosis is one of the most common zoonotic diseases worldwide [8] affecting public and veterinary health. It is reported to be particularly widespread in the Middle Eastern and North African countries. However, many countries do not yet have well-established surveillance systems or the laboratory capacities required to accurately confirm brucellosis in their human or animal populations [9]. This workshop aimed to present the latest recommendations regarding laboratory diagnosis of brucellosis, discuss how the concept of “One Health” can be applied in low-income settings to control the spread of brucellosis, and provide a proper methodology for conducting surveillance studies of zoonotic diseases. The workshop provided an excellent opportunity for public health professionals and officials from health and agricultural ministries of the EMR to reflect on the endemic of brucellosis and what needs to be done to control its spread. Regarding laboratory detection of Brucella species, culturing is the gold standard but is unfeasible owing to its lengthy process. Serological testing may be ideal in reduced-resource settings; however, confirmation requires repeated testing. Surveillance studies in the EMR require a combination of capacity-building activities and awareness-raising activities. The veterinary sector in the EMR can benefit from training on advanced laboratory detection methods. Moreover, updates to animal vaccination strategies are necessary to reduce the loss of livestock. Applying the One Health concept by building multisectoral teams is necessary for the successful implementation of programs aimed at controlling the spread of zoonotic diseases such as brucellosis. Among the lessons learned were the importance of border activity in controlling case numbers within individual nations and creating a demand for multinational or regional collaboration on the issue of brucellosis. Establishing a sustainable communication channel and a culture of collaboration between public health, animal control, and environmental bodies within the government were unanimously championed by workshop facilitators and participants as the way forward to deal with brucellosis and zoonoses. The next steps should include an analysis of the socioeconomic assessment and burden of the disease, as it can be a powerful tool to influence the development of national action plans in EMR countries. Properly designed surveillance programs, which involve the animal health sector as well as the human health sector and use appropriate methods for testing, can provide a more accurate picture of the epidemiology of brucellosis and aid in creating more specific action plans to target and respond to it. The workshop participants made different recommendations including conducting regular awareness sessions for farmers on the benefits of vaccinating their herds and for community members on safe practices for dairy production and consumption. The use of vaccination against *Brucella abortus* in cattle should be covered by animal vaccination programs as it has been isolated in Jordan and Iraq. In any laboratory handling Brucella samples, proper implementation of biosafety and biosecurity guidelines is necessary to prevent laboratory-acquired infections. Due to the scarcity of biosafety level III laboratories in the EMR, serological detection methods may be preferred, as they are less dangerous than culturing methods.

### Identifying Barriers and Strategies to Integrate and Use Noncommunicable Diseases Data Sources

The generation of high-quality health data is a prerequisite for evidence-based decision-making and national planning [10]. An effective health information system (HIS) provides accurate and timely information on health indicators to guide national health system management efforts [11]. The goal of this workshop was to review the fundamentals of HIS and to identify barriers and strategies for the integration and use of noncommunicable diseases data sources, using Jordan as a case study. The workshop highlighted the current efforts in Jordan toward establishing an HIS that caters to noncommunicable diseases. The comprehensive HIS assessment conducted by the World Health Organization (WHO) in 2016 and 2018 was also highlighted. The discussions raised several barriers and challenges toward achieving a comprehensive and consolidated national HIS. One of these barriers is the multisectoral approach. In Jordan, the health care system includes the public sector, private sector, Royal Medical Services, King Hussein Cancer Center, and university hospitals [12], and data exchange between the different health sectors is the ultimate goal of attaining a national HIS. In contrast, the multisectoral approach of the health care system creates difficulties in integrating the HIS, considering the complexities in obtaining approval from the concerned parties to integrate their HIS. There is a lack of useful data, where unnecessary data are sometimes collected, creating technical and financial burdens in translating the data into information. In addition, a low workforce capacity in developing, deploying, and running the HIS is another barrier [13]. Finally, financial constraints and lack of sustainable health system financing prevent the continuation of such an HIS. There was consensus on the importance of a national HIS that caters to noncommunicable diseases in Jordan. Several recommendations were made during the discussion, including the need to develop a noncommunicable disease strategy that identifies national priorities for data collection and establishes a unified mechanism for all sectors. Case definitions of the indicators and targets for noncommunicable diseases should be agreed upon, unified between data sources, and used by the HIS. The WHO’s [14] HIS assessment recommendations should be considered, and action should be taken with regard to establishing committees of representatives from involved sectors; developing a prototype of the dashboard model; developing HIS policy; continuing with Survey, Count, Optimize, Review, Enable assessment annually to monitor the progress of the work; and finally disseminating the data to all stakeholders. Human resource development is key to ensuring that the collected data are analyzed and translated into information that can be used by policy makers for action. A mechanism to govern patient data should be available, with transparency in how the data are collected, analyzed, used, and shared to ensure that patient confidentiality is maintained.

## Roundtables

Several topics were discussed in the roundtable discussions including the role of FETPs in responding to COVID-19; institutionalization of rapid response to public health emergencies in EMR countries; integration of early warning and response (EWAR) with event-based and indicator-based surveillance; sustaining the International Health Regulations (IHR) in Iraq; health systems resilience; strengthening the “One Health” approach; supporting public health research capacity, quality, and productivity in a diverse region such as the EMR; the anticipated future of public health post COVID-19; and COVID-19 vaccines and routine immunization synergies and drawbacks.

### Role of FETPs in Responding to COVID-19: Lessons Learned and Challenges

Established in 1980 by the US Centers for Disease Control and Prevention, the FETPs are competency-based training programs aimed at enhancing the epidemiological capacity of the public health workforce [15]. FETP fellows and graduates in the EMR have contributed significantly to the control of many epidemics in the past and continue to contribute to emerging public health threats, including COVID-19 [16].

FETP graduates worldwide and in the EMR were well engaged in the response to the pandemic, including conducting surveillance activities; conducting screening interviews at POEs; data collection, management, and analysis; risk communications; and other activities. However, it is also important to focus and learn from mistakes, shortcomings, and failures. The major challenges faced during the pandemic dealt with logistics, establishing solid surveillance systems, meeting country-specific needs, and vaccine acceptance. FETP residents, both graduates and in training, gained experience improvising and innovating their resources and abilities to combat the challenges brought by the novel coronavirus. The COVID-19 pandemic confronted field epidemiologists with tough challenges, but it also taught them valuable lessons that will better equip them to be well prepared for future outbreaks and pandemics. This roundtable highlighted the need for continuous technical and financial support to FETPs, in addition to the need to institutionalize FETPs and establish new FETPs in other countries.

### Institutionalization of Rapid Response to Public Health Emergencies in EMR Countries

The IHR dictates the need for state parties to establish the capacity to respond promptly and effectively to public health risks [4]. Consequently, RRTs, the multidisciplinary teams who respond to public health events, are essential to contain the harmful effects of emergencies and coordinate responses in fragile situations such as the EMR. Institutionalization of the rapid response process allows the deployment process to become part of the national system and facilitates a timely and effective response to emergencies [17].

Although the setup of RRTs varies from one country to another, there have been various efforts to build and sustain the capacities of RRTs in the region. It is important to link the RRT, FETP, and EOC structures across the health system and invest in the career development of RRTs and FETP graduates and alumni to better retain and mobilize them. To leverage resources at the regional level and better coordinate workforce mobilization, more institutionalization needs to take place nationally. Moreover, coordinating different capacity-building efforts is imperative for standardizing the curriculum and competencies for RRT training. More work is needed to develop key performance indicators for RRTs and to document the challenges and opportunities in their countries.

### Integration of EWAR With Event-Based Surveillance and Indicator-Based Surveillance

Countries, particularly low-income and middle-income countries, must have EWAR systems incorporated in their surveillance system as required by the IHR that was adopted in 2005. EWAR aids in detecting and responding rapidly to signals and alerts coming from both formal and informal sources and within and outside the health sector to rapidly mobilize required resources in a flexible and responsive manner. The EMR has been one of the regions that is most affected by humanitarian emergencies, including armed conflicts and the influx of displaced people. The EMR currently has 8 active Early Warning Alert and Response Network systems in 7 countries experiencing protracted humanitarian emergencies: Afghanistan, Iraq, Libya, Somalia, Sudan, Syria, and Yemen [18]. Studies have shown that EWAR performance in the EMR has been optimal when looking at the timeliness and completeness of reporting and verification of alert systems. However, the population coverage was low for most, and the Early Warning Alert and Response Network’s main focus of outbreak detection was weakened by the increasing number of other diseases. Currently, the WHO is working on multiple levels of surveillance with the Ministries of Health in the EMR. One of the main challenges is the political commitment toward the surveillance systems in the region’s countries as well as the fragmentation of data in the EMR. The WHO aims to finalize all tools, systems, SOPs, and guidelines and secure the support of the involved partners and stakeholders to integrate surveillance systems into the current Ministries of Health systems by 2025. Multiple EMR countries have worked on both event-based surveillance and integrated disease surveillance and response with the WHO. The region needs to have 1 surveillance system with a unified data collection point, where all information can be located to describe the country’s situation. These systems should have IT infrastructure in the country to collect the required information.

### Health Systems Resilience

During crises, resilient health systems can effectively adapt in response to evolving situations and reduce vulnerability across and beyond the systems. It is a key factor in coping with a crisis such as the economic crisis and the COVID-19 pandemic [19]. Key findings presented during this session included building resilient health systems—as a priority for all member states, adequate investments in health for socioeconomic development, adequate investments in health emergency preparedness, integrated approach to health security and universal health coverage (UHC), the importance of building and strengthening the primary health care (PHC) foundation, investing in the essential public health functions (EPHFs), including all hazard emergency risk management capacities, applying the whole-of-society approach, and attention to vulnerable and marginalized groups. Continuous support is needed for FETP graduates to work toward strengthening surveillance systems, investigating outbreaks, and participating in regional and global efforts to respond to COVID-19. Lessons learned from the current situation in the EMR to strengthen both pandemic preparedness and health systems include the importance of investing in EPHFs, including those required for all-hazards emergency risk management; institutionalized mechanisms for whole-of-society engagement; strengthening the PHC approach for health security and UHC; and promoting enabling environments for research, innovation, and learning.

### Sustaining the IHR in Iraq—Enhancing Multisectoral Coordination in the Face of Conflict

Implementation of the IHR in the EMR comes with unique challenges because of the lack of necessary funding, expertise, and infrastructure needed to develop the capacities for disease surveillance, as stipulated by the IHR [20,21]. In contrast, the advent of the COVID-19 pandemic highlighted that improved compliance with the IHR presents an opportunity to reduce the costs of life and improve the economy [22]. Moreover, the pandemic further emphasized the necessity for international aid and cooperation for the successful implementation of the IHR in low- to middle-income countries [21]. In this regard, Iraq has worked actively to build a solid foundation to establish the necessary tools, facilities, and procedures to comply with the IHR and protect national and international health security. Since 2017, the Government of Iraq (GOI) and Kurdistan Regional Government (KRG) have partnered with Georgetown University to implement the regulations and enhance coordination between the relevant sectors within the country. Great strides have been achieved with regard to IHR compliance through the various governmental bodies in Iraq since the start of the collaboration with Georgetown University in 2017. It is evident that the continuous work to build the core capacities, appoint IHR focal points, and build multisectorial networks has been advantageous in monitoring, reporting, and responding to communicable disease cases across all the governorates of the GOI and KRG. Nonetheless, the COVID-19 pandemic exposed certain weaknesses of IHR compliance that still need to be addressed, especially regarding rapid communication between all the stakeholders and with the WHO networks, in addition to the need for capacity development, particularly at the POEs and for the zoonotic disease surveillance teams. The panelists recommended unification of all the processes regarding IHR implementation in the KRG, strengthening communication between IHR focal points of the KRG and the GOI and sustaining the relationships between them, and increasing funding for more basic scientific research on viral pathogens. In addition, increasing awareness of the contributions of veterinary laboratories in disease outbreaks, providing better definitions of the roles and responsibilities of the veterinary laboratories of the GOI and KRG, and improving communication between the Central Public Health Laboratories and veterinary laboratories are all necessary for the improved application of the One Health concept. Biorisk capacity-building activities are needed in the KRG to improve preparedness for future PHEOCs. There is also a need for efficient reporting by focal points of various POEs in the country. This requires technology, training, and personnel support for the POEs. Building sustainable networks between all ministries, focal points, and the WHO for IHR compliance is important for continued improvements.

### Strengthening the “One Health” Approach

“One Health” is an integrated unifying approach that aims to sustainably balance and optimize the health of people, domestic and wild animals, and the wider environment [22]. Its area of work includes food safety, control of zoonoses, laboratory services, neglected tropical diseases, environmental health, and combating antibiotic resistance. This roundtable discussion offered an opportunity to present the dynamics of emerging infectious disease pathogens; their impact on global health security; and the integrated solutions, definitions, principles, and institutionalization of One Health. Institutionalization and governance of the One Health strategy, securing political commitment, influencing policy changes, promoting multisectoral collaboration, community engagement for breaking silos, cultural changes for working together, and a better understanding of the interconnectedness and interdependence of human-animal-ecosystems are essential to strengthen the role of One Health. This roundtable provided recommendations regarding strengthening One Health capacity building based on the 5 principles of equity, parity, equilibrium, stewardship, and transdisciplinary. The engagement of nongovernmental organizations, the private sector, and other relevant players will strengthen the role of One Health. Epidemiological data and laboratory information should be shared across sectors to effectively detect, respond to, and prevent outbreaks of zoonoses and food safety problems. Joint responses to health threats and improving surveillance systems, early detection, notification, and management of wildlife diseases should be implemented by government officials, researchers, and workers across sectors at the local, national, regional, and global levels.

### The Anticipated Future of Public Health Post COVID-19

COVID-19 has highlighted the need for better governance, more robust health systems and capacities, and the need to shift the paradigm toward public health and preventive medicine. This sheds light on the importance of coordination and collaboration among countries and stakeholders in different multilateral and global initiatives. Although the focus has rightly been on the immediate response to the virus, it is important to consider what comes next and ensure that lessons learned are followed. Many aspects need to be revised, including collaboration among countries, world trade regulations, budget allocation, partnership with the private sector, and health inequalities. Governments should build on their experiences and sustain the positive impacts of COVID-19 on public health by promoting and facilitating the adoption of lifestyles to reduce environmental pollution, revising tobacco control policies to build on the success of smoking reduction, and facilitating patients with chronic diseases to adopt regular self-care and healthy lifestyles. Governments should also consider aligning international strategies, including partnerships with the private sector; investment in digital technologies to strengthen pandemic management and future preparedness for other infectious diseases; and improving digital inclusivity by providing all society segments, especially the unprivileged, with access to digital skills and appropriate infrastructure. Governments should recognize the root causes of health inequalities and commit to short and medium road map remedy strategies. They should build trust among nations to enhance global collaboration and strengthen a wide network of global public health institutions and laboratories to prepare for any future outbreaks or pandemics by timely sharing of information, viral specimens, and genomic sequences; allocate appropriate research and development funds for the development of vaccines, diagnostics, and therapeutics; and establish regional centers of excellence for the rapid manufacturing of vaccines when needed. The appreciation of health professionals should not be viewed as a short-term response. Leaders must apply incentives to improve workforce retention and empower health professionals at all levels with the skills and equipment required to deal with future public health challenges. We need to enhance and apply complex systems modeling, especially in epidemiology and behavioral science, and strengthen surveillance systems for viruses, especially in birds and animals.

### Supporting Public Health Research Capacity, Quality, and Productivity in a Diverse Region

Public health research plays a critical role in strengthening health systems. However, public health research productivity in the EMR remains below the world average [23]. Many challenges and barriers face public health research capacity, quality, and productivity in the EMR, including lack of funding, problems with data availability, language barriers for publishing, lack of guidelines and regulations, and inadequate research skills and competencies. Researchers conducting research in conflict contexts face more challenges compared with those in many other settings. However, there have been success stories in the EMR regarding research publication and its positive and effective impact on policy makers and decision makers. More research should be conducted in conflict areas to investigate the needs of people living in such areas and, consequently, the appropriate responses to their needs. Official guidelines on data sharing should be developed and should be clear and consistent with all public health data. The guidelines must find a balance between making data available and accessible to researchers and safeguarding privacy. Universities in the EMR must make research skills and competencies part of their undergraduate and postgraduate curricula. Research institutions are encouraged to focus on developing a research capacity educational program such as “train the trainer,” where institutions adopting such a program must be flexible and willing to revise the plan if faced with barriers and challenges. Initiatives to facilitate the publication of research in the EMR must be implemented, such as hiring professional copy editors to read the manuscript before submission to the journal. Knowledge transfer frameworks and programs should be developed and implemented for collaborative knowledge transfer between researchers, policy makers, and other relevant stakeholders to facilitate the linkage between science and policy.

### COVID-19 Vaccines and Routine Immunization Synergies and Drawbacks

Disruptions in health services because of the COVID-19 pandemic have strained health systems, resulting in 22.7 million children missing out on vaccination in 2020 [24]. Most of these children live in communities affected by conflict, underserved remote places, or informal or slum settings facing multiple deprivations including limited access to basic health and key social services [14]. Although access to immunization was hindered in 2020 because of the imposed curfews and deferred or postponed supplementary outreach activities, the Expanded Program on Immunization (EPI) was further challenged in 2021 by bearing the additional constraint of introducing the COVID-19 vaccine and implementing the vaccine uptake, often at the cost of routine pediatric vaccine-preventable diseases in most countries. In the EMR, this disruption varied across countries with respect to their socioeconomic status on the one hand and their pre–COVID-19 system infrastructure on the other hand. Although some countries in the region are struggling with the drastic coverage drop in essential vaccines or the below-target COVID-19 vaccine uptake, other countries succeeded in maintaining their vaccine-preventable disease coverage rates while achieving the WHO target for COVID-19 vaccines. For instance, in Oman, acknowledging that any coverage drop is significant and might jeopardize decades of effort urged the timely monitoring of drops and the timely intervention at lower levels. In Iraq, consolidating national efforts to compensate for the drop and planning and executing the COVID-19 vaccine deployment plan using the available infrastructure was deemed successful. Lessons learned from the region reveal that preparedness is the fundamental pillar for successfully maintaining the functionality of EPI and delivering the COVID-19 vaccine. COVID-19 vaccination highlighted the availability of opportunities in terms of funding, technical and capacity building, and social mobilization. It is of utmost importance to benefit from these investments to strengthen and maintain routine immunization in the future. Embracing that “every child counts” is the fundamental driver to consider when going over the lessons learned, challenges, and success stories of countries. Sharing experience and expertise among countries is essential to accelerate efforts to compensate for the drop in vaccine coverage. In addition, structural adjustments can be implemented at the country level in terms of preparedness to avoid dropping in future disruptions. The introduction of the COVID-19 vaccine highlighted the shift in EPI service delivery from those aged <18 years to vaccine delivery covering all age groups. As this paved the way for other new vaccines that might be introduced in the future to various age groups, it is important to translate all the lessons learned and investments into sustainability in terms of quality and not just quantity of EPI service delivery.

## Keynote Speakers’ Sessions

### EPHFs and the UHC Challenge in the EMR

EPHFs are important for achieving the public health goal of improving, promoting, protecting, and restoring population health. UHC aims for all people and communities to have access to quality health services without financial hardship, which in turn improves health security and resilience to crisis.

In 2013, EMR countries endorsed UHC as a priority and developed a regional framework for action to advance UHC. The framework includes strategic actions for countries to achieve UHC and enhance financial risk protection to reduce out-of-pocket spending and financial hardship [25]. Several countries in the EMR are involved in the process of assessing their public health system strengths and weaknesses. To date, 2 countries, namely, Qatar and Morocco, have undertaken the formal assessment of the EPHFs using the EMR assessment tool [26,27]. The COVID-19 pandemic provides an opportunity for strengthening EPHFs and reinforcing health security that should not be missed. COVID-19 has highlighted the urgent need for a global commitment to make health systems resilient against all public health threats to sustain the progress toward health security and UHC. The pandemic has emphasized the lack of preparedness around the world and the need for stronger health systems to address pertinent gaps. Assessments of health systems can help reveal these gaps, but assessments need to be owned by the countries and engage all stakeholders. Strengthening EPHFs is the most cost-effective and sustainable way of achieving UHC.

### Evidence, Experience, and Expertise: Lessons From the US Coronavirus Disease Public Health Response

The COVID-19 outbreak control requires synergic and multisectoral pillars: continuous support of epidemiological and genomic surveillance that is crucial for the detection and identification of circulating and potential new emerging variants; enhancing surveillance in specific congregates of high-risk groups to timely detect and stop the spread of the outbreak; and increasing vaccine demand and booster uptake paralleled with the clarification of rumors and misinformation. Maintaining adequate epidemiological surveillance is crucial for learning about the effectiveness of the COVID-19 vaccines against the new variants. Conducting prompt investigations of outbreaks in highly vaccinated areas or populations in addition to adequate genomic surveillance are key priorities to learn about virus dynamics and mutations and to provide knowledge that would benefit vaccine production. In addition, the use of telemedicine was beneficial during the lockdown, as it allowed people with comorbidities to seek medical care. It is important to build on this success to strengthen the infrastructure of telemedicine in the near future.

### Learning From COVID-19: What It Would Take to Be Better Prepared

The political instability and fragile health systems in some EMR countries have hampered the effectiveness and efficiency of the strategies adopted to combat the COVID-19 pandemic [28]. Over time, this region has witnessed many outbreaks, including Middle East Respiratory Syndrome, cholera, polio, and increasing vector-borne and zoonotic outbreaks [29]. The region is also burdened by the refugee crisis and numerous internally displaced people. Multiple factors affected transmission, including conflict, demographics, timeliness of travel and social restrictions, migrant workers, and mass gatherings or pilgrimages. Countries that responded well to COVID-19 had a high level of political commitment, with multisectoral coordination. In addition, existing infrastructures including polio teams and regional laboratories were quick to mobilize and build on polio and influenza infrastructure. There have been several innovations, including applications, telemedicine, hotlines, e-clinics, and solar-powered oxygen generators. Most countries have banned waterpipe smoking and established mental health hotlines. However, the pandemic has also highlighted the weak epidemiological capacity of the region, the fragmented surveillance systems, and the lack of trust. Different areas of improvement were also highlighted. At the country level, there is a need to invest in building human capacities including epidemiologists, emergency responders, community health workers, health economists, communication specialists, and, most crucially, health leaders; strengthen health systems and work toward UHC and health security; work toward community engagement and community trust; and develop and update a multisectoral emergency preparedness plan. At the regional level, certain countries have greater capacities than others do in the region, and there is a need for more cooperation, solidarity, and support to effectively control the spread of the pandemic. Rich countries should ensure vaccine sharing, equity, and distribution with low-income countries. Moreover, countries should implement twinning programs in which human resources are shared across countries.

### Reshaping Public Health After the Pandemic Era: The Agenda for the Next Decade—Are We Ready?

In 2020, the WHO conducted a global pulse survey to understand the impact of COVID-19 on the health system. Almost 90% of the 105 engaged countries reported an interruption to a different type of services that ranged from routine and elective service delivery to critical care, especially in low- and middle-income countries. There are many sources of disruptions. Financial constraints, supply chain disruptions, redirection of services to care for patients with COVID-19, and unavailability of the workforce are some of the factors affecting accessibility to essential health care services [30]. The findings demonstrated that investment in PHC is essential to mitigate the risks of future pandemics and to maintain the accessibility and delivery of essential health services during emergencies [31]. Investment in the health workforce is another dimension for a successful response, which includes training, mobilization, and redistribution to sustain high-quality essential health service delivery. Therefore, the COVID-19 pandemic serves as an opportunity for countries to reshape their public health to improve public health security for future pandemics. Integrating public health into primary care and investment in public health workforce capacity building is an essential approach for reshaping public health. A total of 6 models were identified by the WHO technical series on primary care called “Closing the Gaps Between Public Health and Primary Care Through Integration” to attain the integration of public health into PHC. This, in return, will focus the services on the population needs and achieve the person-centered approach. These models can be applied either individually or in combination depending on the flexibility of the health systems [32].

### COVID-19–Resilient PHC: Experience and Challenge in United Nations Relief and Works Agency for Palestine Refugees in the Near East

The United Nations Relief and Works Agency for Palestine Refugees in the Near East (UNRWA) provides assistance and protection to Palestine refugees in Jordan, Lebanon, Syria, the Gaza Strip, and the West Bank, including East Jerusalem. The UNRWA has adopted multiple mechanisms to address the various health care needs. The adopted mechanism allowed for the effective sustainability of services for UNRWA PHC clinic services during the pandemic. In addition, using innovative and electronic services allowed the community access to essential health care services when needed. For this, health care staff must adopt available protection measures such as vaccination and infection protection and control practices. In addition, embracing the new normal for PHC UNRWA clinics is important to ensure continuity of services and inclusion of vulnerable community groups. Many actions can be taken to promote the sustainability of PHC services during the COVID-19 pandemic or any future outbreak. These actions include the need to improve the UNRWA system to include a resilient family medicine approach and services that adopt innovative digital systems and programs. There is a need to focus on strengthening the capacity of human resources by providing them with continuous education and clear career paths that ensure their development. In addition, COVID-19 brought organizations together, and partnerships proved to play a vital role in the ability to respond to outbreaks and emergencies. In addition, focusing on staff vaccination and protection measures and ensuring that UNRWA staff are well protected is the key to ensuring service sustainability. Supporting UNRWA through donations is another action that can be taken to sustain and enhance PHC at UNRWA clinics.

### The Cohesion of Society During and After a Pandemic: How Does This Translate in the EMR Public

Social cohesion refers to the strength of relationships and the sense of solidarity among members of a community [33]. A group of researchers from the Bertelsmann Foundation studied social cohesion during the COVID-19 pandemic in 2020 to examine the fabric of society during the 3 waves of the pandemic in Germany. The results showed that social cohesion remained stable during the first wave, leading to greater visibility of solidarity in some areas. People in the middle and higher socioeconomic categories were more satisfied and confident in the pandemic response measures than those in the other categories. In the second half of the year, concerns about the future increased among all groups surveyed. In terms of division, middle-aged and low-educated respondents living in unsafe circumstances felt that society is highly divided. This was accompanied by lower levels of trust, a more significant rejection of pandemic response measures, and a growing fear that societal consensus is unattainable. Solidarity is a value that should be learned. These values are not always at the forefront of political choices when building healthy societies. The concept of solidarity is based on the idea that people can unite their differences because they have a common interest in not being divided and conquered. It is important to enhance global cohesion within the current inequalities between low-income and high-income countries, and it is recommended to enhance 2-way communication with public health professionals. Both sides must exchange information and decisions. Governments are responsible for educating and training their health workforce and improving their job conditions to minimize brain drains.

## Conclusions

The seventh EMPHNET regional conference was dedicated to exploring ways to achieve public health resilience. To this effect, EMPHNET adopted the theme of *Towards Public Health Resilience in the EMR: Breaking Barriers*. Conference sessions provided highly promising opportunities to explore ways to achieve such goals in the EMR. The conference sessions shed light on the latest scientific findings, important lessons learned, and discussions on ways in which current barriers can be broken down through coordination and collaboration.

## Appendix

Multimedia Appendix 1 Abstract book.

## Abbreviations

CPD: continuing professional development
EMPHNET: Eastern Mediterranean Public Health Network
EMR: Eastern Mediterranean Region
EOC: emergency operations center
EPHF: essential public health function
EPI: Expanded Program on Immunization
EWAR: early warning and response
FETP: Field Epidemiology Training Program
GOI: Government of Iraq
HIS: health information system
IHR: International Health Regulations
KRG: Kurdistan Regional Government
PHC: primary health care
PHEOC: public health emergency operations center
POE: points of entry
RRT: rapid response team
SOP: standard operating procedure
UHC: universal health coverage
UNRWA: United Nations Relief and Works Agency for Palestine Refugees in the Near East
WHO: World Health Organization

## References

[1] Bhaskar S, Rastogi A, Menon KV, Kunheri B, Balakrishnan S, Howick J (2020). Call for action to address equity and justice divide during COVID-19. Front Psychiatry.

[2] WHO’s 7 policy recommendations on building resilient health systems. World Health Organization.

[3] (2020). Handbook for Public Health Capacity-building at ground crossings and cross-border collaboration. World Health Organization.

[4] (2016). International Health Regulations (2005) Third Edition.

[5] Al Nsour M, Bashier H, Al Serouri A, Malik E, Khader Y, Saeed K, Ikram A, Abdalla AM, Belalia A, Assarag B, Baig MA, Almudarra S, Arqoub K, Osman S, Abu-Khader I, Shalabi D, Majeed Y (2020). The role of the global health development/eastern mediterranean public health network and the eastern Mediterranean field epidemiology training programs in preparedness for COVID-19. JMIR Public Health Surveill.

[6] Griffith M, Ochirpurev A, Yamagishi T, Nishiki S, Jantsansengee B, Matsui T, Oishi K (2018). An approach to building Field Epidemiology Training Programme (FETP) trainees' capacities as educators. Western Pac Surveill Response J.

[7] Campbell C, Silver I, Sherbino J, Cate OT, Holmboe ES (2010). Competency-based continuing professional development. Medical Teacher.

[8] Seleem MN, Boyle SM, Sriranganathan N (2010). Brucellosis: a re-emerging zoonosis. Vet Microbiol.

[9] Rubach MP, Halliday JE, Cleaveland S, Crump JA (2013). Brucellosis in low-income and middle-income countries. Current Opinion Infect Dis.

[10] Sorenson C, Chalkidou K (2012). Reflections on the evolution of health technology assessment in Europe. Health Econ Policy Law.

[11] (2008). Toolkit on monitoring health systems strengthening: health information systems. World Health Organization.

[12] The Higher Health Council (2015). The National Strategy for Health Sector in Jordan 2015- 2019.

[13] Jabareen H, Khader Y, Taweel A (2020). Health information systems in Jordan and Palestine: the need for health informatics training. East Mediterr Health J.

[14] COVID-19 pandemic leads to major backsliding on childhood vaccinations, new WHO, UNICEF data shows. World Health Organization.

[15] Field epidemiology training programs. The Eastern Mediterranean Public Health Network (EMPHNET).

[16] FETP activities in response to coronavirus disease 19 (COVID-19). TEPHINET.

[17] Global Health Security Agenda homepage. Global Health Security Agenda.

[18] Mala P, Abubakar A, Takeuchi A, Buliva E, Husain F, Malik MR, Tayyab M, Elnoserry S (2021). Structure, function and performance of Early Warning Alert and Response Network (EWARN) in emergencies in the Eastern Mediterranean Region. Int J Infect Dis.

[19] Thomas S, Sagan A, Larkin J, Cylus J, Figueras J, Karanikolos M Strengthening health systems resilience: key concepts and strategies. POLICY BRIEF 36.

[20] Wilson K, von Tigerstrom B, McDougall C (2008). Protecting global health security through the International Health Regulations: requirements and challenges. CMAJ.

[21] Kandel N, Chungong S, Omaar A, Xing J (2020). Health security capacities in the context of COVID-19 outbreak: an analysis of International Health Regulations annual report data from 182 countries. Lancet.

[22] One health. World Health Organization.

[23] Tadmouri GO, Mandil A, Rashidian A (2019). Biomedical and health research geography in the Eastern Mediterranean Region. East Mediterr Health J.

[24] WHO/UNICEF (2021). Progresses and challenges with sustaining and advancing immunization coverage during the COVID-19 pandemic. World Health Organization.

[25] Universal health coverage. World Health Organization.

[26] Alwan A, Shideed O, Siddiqi S (2016). Essential public health functions: the experience of the Eastern Mediterranean Region. East Mediterr Health J.

[27] (2017). Assessment of Essential Public Health Functions in Countries of the Eastern Mediterranean Region.

[28] Brennan R, Hajjeh R, Al-Mandhari A (2022). Responding to health emergencies in the Eastern Mediterranean region in times of conflict. Lancet.

[29] Mostafavi E, Ghasemian A, Abdinasir A, Nematollahi Mahani SA, Rawaf S, Salehi Vaziri M, Gouya MM, Minh Nhu Nguyen T, Al Awaidy S, Al Ariqi L, Islam MM, Abu Baker Abd Farag E, Obtel M, Omondi Mala P, Matar GM, Asghar RJ, Barakat A, Sahak MN, Abdulmonem Mansouri M, Swaka A (2021). Emerging and re-emerging infectious diseases in the WHO eastern Mediterranean region, 2001-2018. Int J Health Policy Manag.

[30] Beyond COVID-19: a whole of health look at impacts during the pandemic response. Center For Global Development.

[31] (2020). Maintaining essential health services: operational guidance for the COVID-19 context: interim guidance. World Health Organization.

[32] Ho K, Al-Shorjabji N, Brown E, Zelmer J, Gabor N, Maeder A, Marcelo A, Ritz D, Messina L, Loyola M, Abbott P, Nazira J, McKinnon A, Dissanayake V, Akeel A, Gardner N, Doyle T (2016). Applying the resilient health system framework for universal health coverage. Stud Health Technol Inform.

[33] Kawachi I, Berkman LF (2000). Social cohesion, social capital, and health. Social Epidemiology.

