# Supplementary material for: Toward Public Health Resilience in the Eastern Mediterranean Region: Findings From the Seventh Eastern Mediterranean Public Health Network Regional Conference
Source: Interact J Med Res. 2023 Mar 9;12:e36356. doi: 10.2196/36356 (PMC10037172; doi:10.2196/36356)
Supplement: Multimedia Appendix 1 [file ijmr_v12i1e36356_app1.pdf]

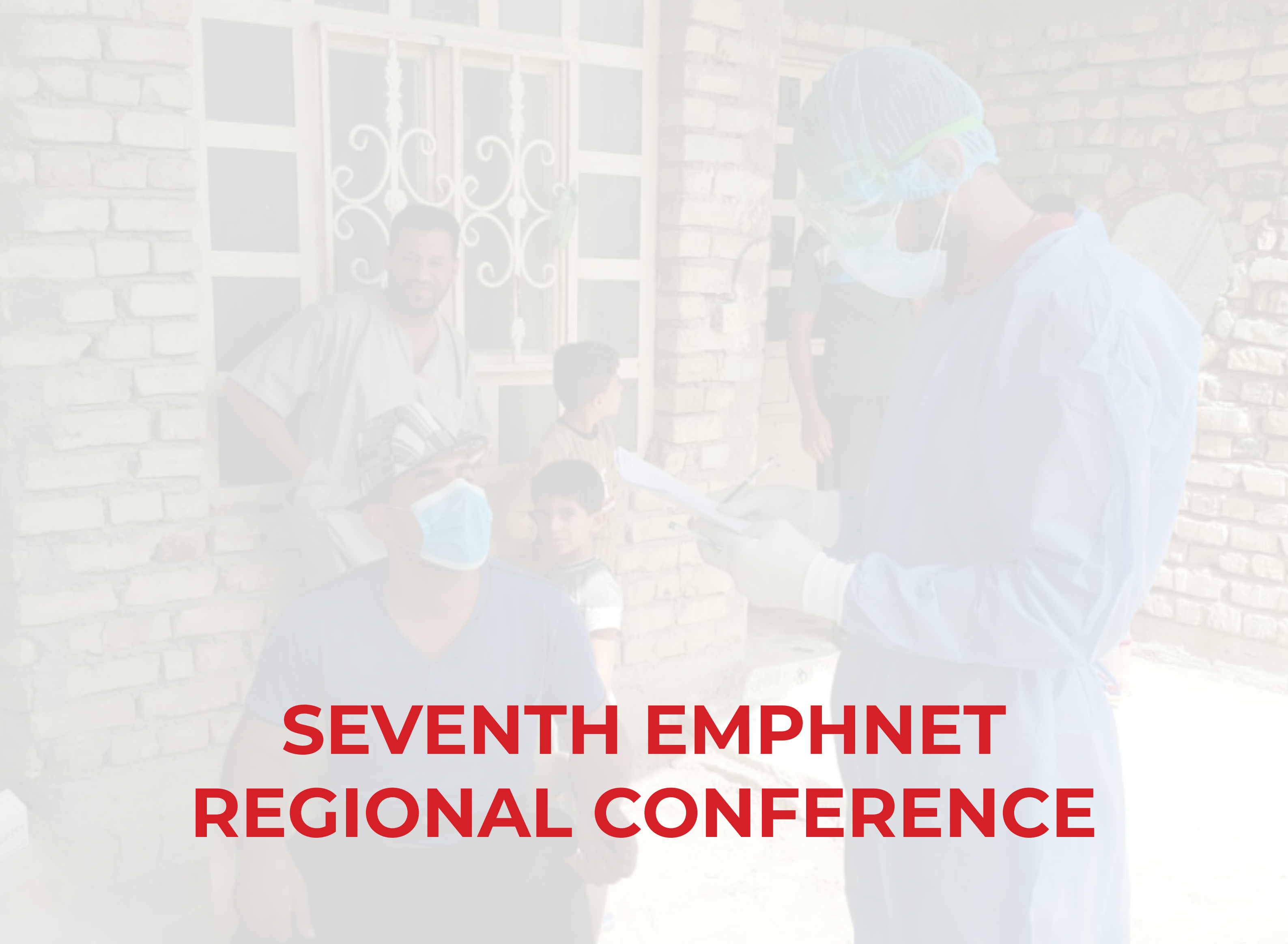

SEVENTH EMPHNET REGIONAL CONFERENCE

Towards Public Health Resilience in the Eastern Mediterranean Region: Breaking Barriers

Amman, Jordan
November 14-18, 2021
Landmark Hotel

Abstract Book

Seventh EMPHNET Regional Conference
Towards Public Health Resilience in the EMR: Breaking Barriers

EMPHNET
The Eastern Mediterranean Public Health Network

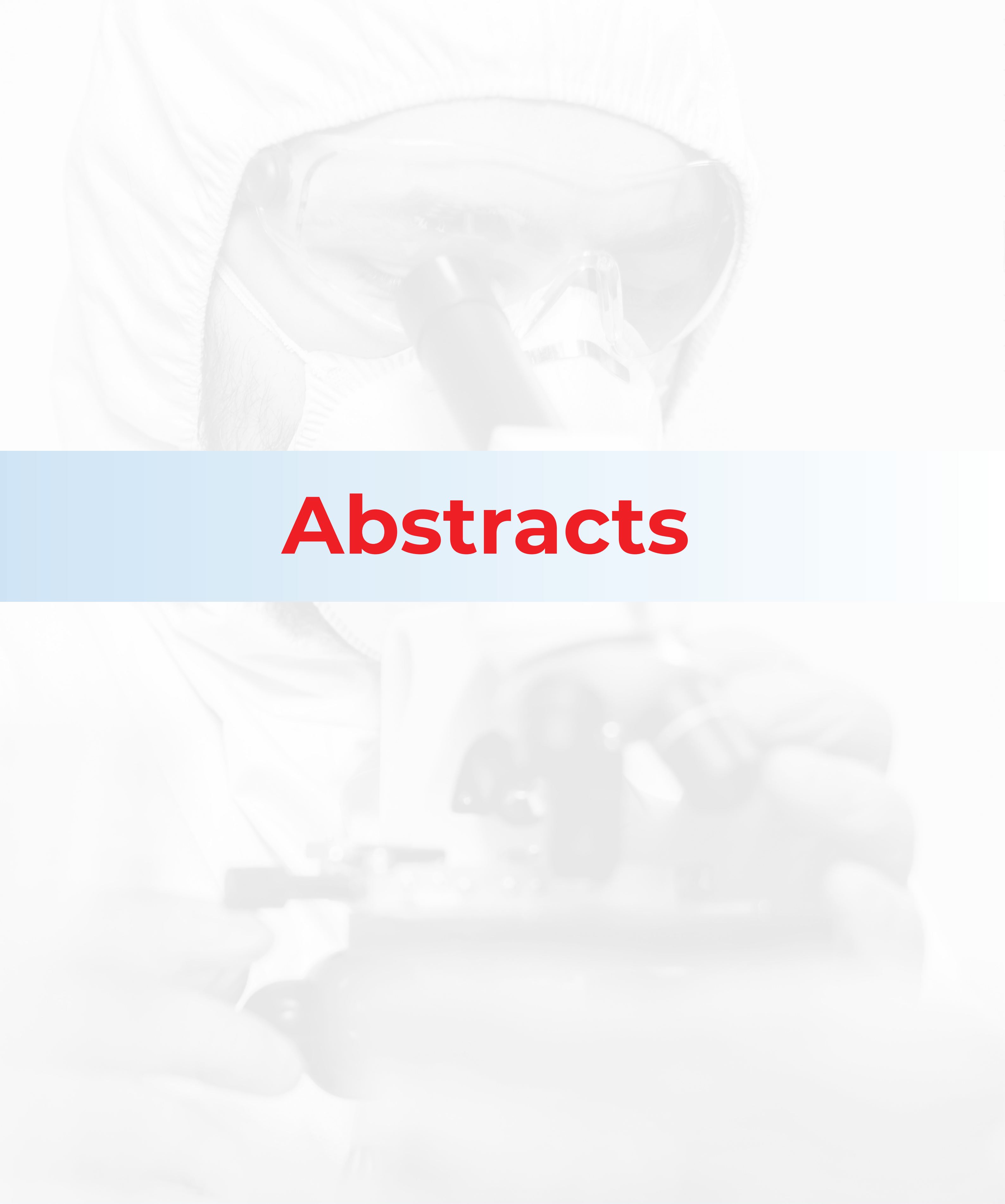

Abstracts

9 - Abstracts (Day 2, November 15th, 2021 Session 1)

COVID-19

Abstract Code: 2021-EOH-777
Presenter Name: Essam Mostafa
Country: Egypt
Presenter Email: essammostafafoka1414@yahoo.com
Authors: Dr. Alaa Eid, Dr. Ehab Attia, Dr. Hala BahaaEldin, Dr. Hanaa Abu Elsood, Dr. Mohamed Abdel-fattah, Dr. Sahar Samy, Dr. Salma Afifi

Title: The Impact of COVID-19 Pandemic on Healthcare-associated Infections in Intensive Care Units: Results from the Egypt National Healthcare-associated Infections surveillance, 2019-2020

Background:
COVID-19 pandemic resulted in unexpected influx of patients leading to high rates of hospitalization. Focusing resources to mitigate pandemic unintentionally reduced attention to healthcare-associated infections (HAIs) prevention programs. Intensive care units (ICUs) have suffered most burden due to requirement of ventilation.

Purpose:
We estimated the national HAIs rates at ICUs before and during COVID-19 pandemic to better identify pandemic impact on HAIs.

Methodology:
Egypt HAI surveillance was established in 2016 in 177 governmental ICUs. CDC case definitions and questionnaire were used to collect patients' data. The types of HAIs targeted included bloodstream infections (BSI), pneumonia, and urinary tract infections (UTIs). Pathogens identification and antimicrobial resistance were performed at the Central laboratory. Surveillance data 2019-2020 were obtained and descriptive data analysis was performed. HAIs Rates per 100 patient-days, and device-associated infections (DAIs) per 1000 device-days were compared between 2019 and 2020.

Results:
In 2020, 4,028 HAIs were reported including 777(19.3%) ICU-acquired while in 2019, 6,242 HAIs were reported including 1,084 (17.4%) ICU-acquired. The incidence significantly decreased in 2020 compared with the year 2019 (2.67 vs 2.72, p<0.001). Percents of BSI, pneumonia, UTI in 2020 vs. 20019 were (64.0% vs. 61.6% and 10.9% vs. 12.1% and 25.1% vs. 23.8%). DAIs decreased significantly including CLABSI (2.6 vs. 2.5, p<0.001), VAP (0.75 vs. 0.87, p=0.04), CAUTI (1.5 vs 1.6, p=0.02). Klebsiella spp. was the predominant pathogen in both years representing (35.6 and 38.1%), followed by S. aureus (11.2 and 15.4%). Rate carbapenem-resistant K. pneumoniae insignificantly increased (25% vs. 23%, p=0.3) and Methicillin-resistant S. aureus decreased (68% vs 70%, p=0.4).

Conclusions:
Egypt HAI surveillance successfully described impact of COVID-19 pandemic on HAIs. It identified significant decrease in ICU-acquired HAIs and DAIs at first pandemic year, that could reflect better infection control measures. Types of HAIs, causative pathogens and antimicrobial resistance pattern did not change significantly. Surveillance should be maintained to guide HAIs preventive and control measures.

Keywords:
COVID-19, Hospital Acquired infection, Intensive care unit, Device associated infection.

9 - Abstracts (Day 2, November 15th, 2021 Session 1)

COVID-19

Abstract Code: 2021-EOH-735
Presenter Name: Ghadeer Musleh
Country: Jordan
Presenter Email: ghadeermusleh71094@yahoo.com
Authors: Dr. Ala'a Al-Tammemi, Dr. Ghadeer Musleh, Mr. Ahmad Malkawi, Mr. Hussam Fakhouri, Ms. Muna Barakat, Ms. Rama Kanj

Title: Online Teaching and the Psychological Status of University Teachers During COVID-19

Background:
The COVID-19 pandemic has struck many countries globally. Jordan has implemented strict nationwide control measures to halt the viral spread, one of which was the closure of universities and shifting to remote teaching. Online teaching has had several consequences including psychological effects.

Purpose:
To assess the psychological status of university teachers in Jordan, challenges of online teaching, and coping practices during the COVID-19 pandemic.

Methodology:
This was a cross-sectional study using an anonymous online survey. The measure of psychological distress was obtained using a validated Arabic version of the Kessler Distress Scale (K10). Other information collected included the sociodemographic profile, methods used to handle distress, motivation to participate in online teaching, challenges of online teaching, and issues that concern teachers during this pandemic.

Results:
Three hundred eighty-two university teachers (n=382) returned completed surveys. Findings of the Kessler Distress Scale showed that 31.4% of respondents had severe distress and 38.2% had mild to moderate distress. The only significant factors associated with distress severity was age, which showed a weak negative correlation (Rho = -0.19, P < 0.0001). Interestingly, most teachers had moderate to high motivation for online teaching. Engagement with family was the most reported self-coping practice. More than half of the participants were most concerned and fearful about the COVID-19 infection.

Conclusions:
University teachers exhibited various levels of psychological distress during the implementation of precautionary national measures in the battle against COVID-19 in Jordan. It seems that healthy family relationships has many advantages, including the ability to cope with the encountered stress.

Keywords:
Jordan, University, Online, COVID-19, Stress, Psychological Status.

Seventh EMPHNET Regional Conference
Amman, Jordan

9 - Abstracts (Day 2, November 15th, 2021 Session 1)

COVID-19

Abstract Code: 2021-MCH-722
Presenter Name: Hiba Thabit
Country: Iraq
Presenter Email: heba.mohammed1300a@comed.uobaghdad.edu.iq
Author: Dr. Hiba Thabit

Violence Against Healthcare Workers (HCWs) During the COVID-19 Pandemic in Baghdad, Iraq, 2020

Background:
Workplace violence (WPV) is defined as events where employees are exposed to abuse or threats in situations associated with their work. Recently, violence against healthcare workers was reaching to peak levels worldwide.

Purpose:
To estimate the prevalence and identify the profile of WPV, perceived causes, and its consequences against a sample of Iraqi HCWs in Baghdad during the COVID-19 pandemic year, 2020.

Methodology:
A cross-sectional online study was conducted on a sample of 311 HCWs working in a number of hospitals and PHCs in Baghdad from January to April 2021. Data was collected using a self-administered questionnaire to gather data on demographic and job characteristics, exposure to violence, the circumstances, the response of the victims and the institutions, and the impact of the violence on the victims.

Results:
The total participants were 311 HCWs with a mean working year at service of 6.75 ± 5.35 years. A total of 215 (69.3%) participants had experienced violence in 2020, and 97 (45%) of them related the violence to COVID-19 circumstances. Verbal violence was reported by 152 (71%), mixed verbal and physical violence was reported by 28 (13%), and 12 (6%) of study participants were exposed to physical violence. Exposure to violence was significantly higher among doctors (78.4%, 127, P=0.001), and those working in the emergency ward (82.6%, 67, P=0.04). About 87 (40.5%) participants did nothing against violence, while 51 (23.7%) reported this to the manager, and the majority of participants 155 (71.6%) reported that the health institute did nothing against violence. As a consequence of violence, around 68% (141) of workers complained of mental issues and a decrease in performance.

Conclusions:
Violence against HCWs is considerably high, particularly among doctors and in the emergency wards. It affected doctors and HCWs at the emergency ward more than others. Further work is needed to improve the communication skills of HCWs and to make the workplace safer.

Keywords:
Violence, Workplace, Healthcare workers, Iraq.

9 - Abstracts (Day 2, November 15th, 2021 Session 1)

COVID-19

Abstract Code: 2021-FWBD-725
Presenter Name: Samsor Rahat
Country: Afghanistan
Presenter Email: samsoorrahat@gmail.com
Author: Dr. Khawaja Mir Islam Saeed

Title: Effects of the COVID-19 Pandemic on Routine Immunization Coverage – Afghanistan, 2020

Background:
Routine immunization is a vital component in reducing morbidity and mortality. During CCOVID-10 pandemic, essential health services including immunization were disrupted.

Purpose:
This study aimed to compare routine immunization services during pandemic/lockdown period in 2020 as well as normal situation in 2019.

Methodology:
A descriptive study was conducted to analyze secondary data existed in the EPIMIS database at national level in Ministry of Public Health. The data was extracted from database from April-July for 2019 and 2020. Immunization coverage for measles, tetanus and PENTA vaccines were calculated and compared. A descriptive study was conducted to analyze secondary data existed in the EPIMIS database at national level in Ministry of Public Health. The data was extracted from database from April-July for 2019 and 2020. Immunization coverage for measles, tetanus and PENTA vaccines were calculated and compared.

Results:
The routine immunization coverage dropped 11%, 2% and 12% for Penta-3, Measles-1 and Tetanus Toxoid 2nd dose (TT2+ Pregnant women) respectively during COVID-19 Pandemic lockdown period (April to July, 2020) as compared to 2019 for the same period. Moreover, coverage at various provincial levels also decreased due to COVID-19 pandemic. The highest decreased coverage for PENTA-3 and Measles-1 was observed 45% and 43% in Paktya province during COVIDd-19 pandemic as compared to the same period of 2019. In addition, the highest TT2+ coverage for pregnant women dropped in Kunduz province which is 67%. During lockdown, the fixed sessions declined 3% while outreach sessions declined by 1%. Overall, 202,408 children in fixed and 24,173 children in mobile sessions were missed in lockdown period due to COVID-19 as compared to the same period of 2019.

Conclusions:
COVID-19 pandemic has adversely affected the immunization as part of essential health services. It will further reduce the already low level of immunization in Afghanistan, which has led to child and mother morbidity and mortality. During pandemic, the government should focus and maintain essential health services.

Keywords:
Afghanistan, Immunization, Measles, Tetanus, COVID-19.

Seventh EMPHNET Regional Conference
Amman, Jordan

9 - Abstracts (Day 2, November 15th, 2021 Session 1)

COVID-19

Abstract Code: 2021-EOH-710
Presenter Name: Shahd Ramz
Country: Iraq
Presenter Email: Shahed.Raed1300a@comed.uobaghdad.edu.iq
Author: Dr. Shahd Ramz

Title: Long-term Sequel of COVID-19 Among Recovered Patients, Baghdad, Iraq, 2020

Background:
Typically, people recover from COVID-19 after 2 to 6 weeks. For some people, however, some symptoms may persist or recur for weeks or months following recovery. Increasing reports from around the world demonstrated that some patients with COVID-19 may suffer from a certain medical sequel and long-term sequelae.

Purpose:
To identify the types, the proportion, and the risk factors of long-term sequel among a sample of COVID-19 patients who recovered for at least two months, Baghdad, Iraq, 2020.

Methodology:
This cross-sectional study was conducted in three Primary Healthcare Centers that were selected using a systematic cluster random sample. All the registered COVID-19 patients in the catchment area of these centers were reviewed, and those who recovered from COVID-19 infection for a minimum of two months were considered eligible. The patients were contacted by telephone to have their approval. A questionnaire was used to gather demographic data, disease characteristics, and data concerning the long-term medical sequelae and filled through the telephone interview after obtaining patients' verbal approval.

Results:
Among 350 illegible patients, 239 (68.3%) patients responded. The long-term sequelae were reported by 103 (43.1%) individuals who seek medical care and 25% reported more than one symptom. The most frequent manifestations were headache (25.1%), fatigability (22.6%), sleep problems (19.2%), concentration deficit (18.8%), cough (18.0%), loss of smell (17.6%), memory deficit (15.9%), loss of taste (14.6%), fainting (13.4%), chest pain (9.2%), hearing deficit (8.4%), and tremor (5.4%). Reporting long-term sequel was significantly higher in older patients, having comorbidities, patients treated at the hospital, and patients who needed ICU admission or oxygen therapy ($P<0.05$). There was no significant association between reporting long-term sequel and gender, educational level, or smoking status ($P>0.05$).

Conclusions:
The long-term sequelae after recovering from COVID-19 are common. A close, long-term follow-up after recovery should be considered particularly for high-risk patients.

Keywords:
Long COVID, Iraq, Prevalence, Risk Factors

9 - Abstracts (Day 2, November 15th, 2021 Session 1)

COVID-19

Abstract Code: 2021-EOH-675
Presenter Name: Shereen Elghazaly
Country: Egypt
Presenter Email: drshereen84@yahoo.com
Authors: Dr. Alaa Eid, Dr. Hanaa Abu El Sood, Dr. Mohamad AbdelFatah, Dr. Mohamed Hassany, Dr. Sahar Samy, Dr. Salma Afifi, Dr. Yousef Khader

Title: The Impact of Comorbidities on COVID -19 Severity and Mortality in Egypt

Background:
Older population and people of any age with underlying certain comorbidities such as diabetes mellitus, cardiovascular, lung disease, kidney disease, liver disease and cancer are at higher risk of severe disease course and death if they become infected with COVID-19. Identifying risky group and risk factors for COVID-19 severity and mortality is important for guiding efficient and appropriate prevention and management of patients with COVID-19.

Purpose:
This study aimed at describing demographics and epidemiologic characteristics of confirmed COVID-19 cases in Egypt and determine the impact of different comorbidities on patients' outcomes.

Methodology:
Data of all confirmed COVID-19 patients admitted to 408 governmental hospitals all over Egypt during February-May 2020 were collected retrospectively from the National Egyptian Disease Surveillance System. Cases were confirmed using RT-PCR.

Results:
Overall, 28,415 patients (55.0% males, 45.0% females) were identified. Their median age was 44 years. Of those, 743(2.6%) were admitted to ICU, 408 (1.4%) required ventilator and 1,045 (3.7%) died. Of 21,617 (76.1%) patients with completed data, 4,687 (21.7%) had comorbidities. Overall , 11.8% had diabetes, 5.3% had cardiovascular disease and 4.3% had chronic obstructive pulmonary disease. Those with one comorbidity were more likely to die (OR = 2.83), admitted to ICU (OR = 6.36) and need ventilator (OR = 5.95) compared to patients with no comorbidities. Having multiple comorbidities increased risk of mortality (OR = 3.53), ICU admission (OR = 8.62), and requiring ventilator (OR = 9.06).

Conclusions:
COVID-19 Patients with comorbidities had higher risk of disease severity and mortality. Multiple comorbidities further increase the risk to higher extent. All necessary precautions should be taken for patients with comorbidities to avoid COVID-19 infection to prevent the worst prognosis.

Keywords:
COVID-19, comorbidities, mortality, severe outcome, public health surveillance.

9 - Abstracts (Day 2, November 15th, 2021 Session 1)

Maternal, Child, and Reproductive Health

Abstract Code: 2021-EOH-732
Presenter Name: Areej Aljasser
Country: Saudi Arabia
Presenter Email: dr.aljasser@hotmail.com
Authors: Dr. Adel Alotaibi, Dr. Eman Elsayed Abd-Ellatif

Title: Neonatal COVID-19 Infection - Al Dawadmi, Saudi Arabia, March 2020: A Case Report

Background:
Saudi Arabia reported its first case of COVID-19 on 2 March 2020, and by 24 March 2020, cases had occurred throughout the Kingdom. Vertical transmission and post-infection immunity for COVID-19 have not been completely established. On 29 March 2020, the Saudi Arabian Field Epidemiology Training Program (FETP) was notified of a potential case of COVID-19 in a neonate in Al Dawadmi, Saudi Arabia.

Purpose:
We sought to determine the source of infection and mode of transmission and identify mitigation measures to prevent further spread.

Methodology:
Working with the Regional Preventive Medicine Department, we interviewed the case's parents and reviewed medical records. We collected data on demographics, clinical course, and contact history. We observed hospital infection control practices and collected data on COVID-19 infection among obstetrics staff and patients.

Results:
AWe identified one case of neonatal COVID-19. He was born through normal vaginal delivery at Hospital A and discharged the next day. The 3-day-old boy was brought to Hospital B on 27 March 2020 with symptoms of fever, cough, and rhinorrhoea. PCR analysis of a nasopharyngeal swab from the neonate was positive for SARS-CoV2. Complete blood screening and chest X-Ray were normal. We isolated the parents who tested negative for SARS-CoV2. We visited the family home for contact tracing. We identified ten household contacts: none reported any symptoms, and all refused to be tested. The case was discharged four days after admission.

Conclusions:
A neonatal case of COVID-19 had mild illness and recovered. At Hospital A, we observed staff donning PPE correctly, and no infections were reported in obstetric ward healthcare staff, other neonates, or mothers. Our investigation highlights the importance of testing all contacts to better understand the source of infection, as these are the primary unknown contact for the infant.

Keywords:
Neonatal COVID-19, Vertical transmission, SARS-CoV-2.

9 - Abstracts (Day 2, November 15th, 2021 Session 1)

Maternal, Child, and Reproductive Health

Abstract Code: 2021-EOH-649
Presenter Name: Dalal Youssef
Country: Lebanon
Presenter Email: dalalyoussef.esu@gmail.com
Authors: Dr. Atika Berry, Dr. Janet Youssef, Dr. Linda Abou Abbas

Title: Knowledge, Attitudes and Practices Among Lebanese Obstetricians and Gynecologists Toward Coronavirus Disease-2019 (COVID-19) and Pregnancy

Background:
The pandemic of Coronavirus Disease 2019 (COVID-19) has seriously disturbed the daily life of the general population particularly the life of pregnant women. Since obstetricians and gynecologists (OBGYNs) are often the primary health care providers during pregnancy, hence the importance of their critical role in preventing and managing COVID-19 infection in their patients.

Purpose:
This study aimed to assess the knowledge, attitudes, and practices of OBGYNs in order to identify existing gaps and to improve patient and occupational safety.

Methodology:
A cross-sectional study, using an online survey, was conducted during the rapid rise of the COVID-19 pandemic in Lebanon over the period extending between 20th October and 20th November 2020 among Lebanese OBGYNs. Knowledge, attitude, and practice scores were computed. A good level of knowledge was defined when the percentage of correct answers reaches more than 80% of the respondents. The analysis was performed using Statistical Package for Social Sciences (SPSS) software.

Results:
A total of 279 OBGYNs participated in the survey of which 57% were males. The majority of them (64.2%) were aged more than 45 years, married (79.9%) with large work experience (70.3%). Only 28.3% were reluctant to take care of COVID-19 patients. Most of them feared contracting COVID-19 or transmitting COVID-19 to their family member due to occupational exposure and 42.3% felt overwhelmed. 62.7% of them considered that policies implemented by the ministry of public health are sufficient. The majority of OBGYNs had a good level of knowledge in different basic and specific domains related to COVID-19 and pregnancy. Furthermore, a good practice score in all relevant aspects (personal, clinic, and patient) was revealed.

Conclusions:
The high knowledge and practice scores among Lebanese OBGYNs disclose a strong commitment from the part of these physicians to fulfill their responsibilities during this pandemic towards themselves and their patients.

Keywords:
Knowledge, attitudes, practices, pregnancy, COVID-19, Obstetricians, gynecologist

9 - Abstracts (Day 2, November 15th, 2021 Session 1)

Maternal, Child, and Reproductive Health

Abstract Code: 2021-OTH-607
Presenter Name: Huda Basaleem
Country: Yemen
Presenter Email: hudabasaleem@gmail.com
Authors: Dr. Eman Al-Kubati, Dr. Fouad Othman, Dr.Fouzia Shafique, Dr. Hannah Tappis, Dr. Iman Al-Gawfi, Dr. Nagiba AlShawafi, Dr. Nuzhat Rafique, Dr. Paul Spiegel, Dr. Sarah Elaraby, Dr. Shatha Elnakib

Title: Reproductive, Maternal, Newborn and Child Health Service Delivery During Conflict in Yemen.

Background:
Armed conflict, food insecurity, epidemic cholera, economic decline and deterioration of essential public services present overwhelming challenges to population health and well-being in Yemen.

Purpose:
To examine how reproductive, maternal, newborn, child and adolescent health and nutrition (RMNCAH+N) services have been delivered since 2015, and identifies factors influencing implementation of these services in three governorates of Yemen.

Methodology:
Content analysis methods were used to analyze available documents and datasets published since 2000 as well as 94 semi-structured individual and group interviews conducted with government officials, humanitarian agency staff and facility-based healthcare providers and six focus group discussions conducted with community health midwives and volunteers in September-October 2018.

Results:
Humanitarian response efforts focus on maintaining basic services at functioning facilities, and deploying mobile clinics, outreach teams and community health volunteer networks to address urgent needs where access is possible. Attention to specific aspects of RMNCAH+N varies by location, levels of violence, humanitarian access and availability of qualified human resources. Health services for women and children are considered to be a priority; however, cholera control and treatment of acute malnutrition are given precedence over other services along the continuum of care. Although health workers display notable resilience, challenges resulting from insecurity, limited functionality of health facilities, and challenges in importation and distribution of supplies limit the availability and quality of services.

Conclusions:
Challenges to providing quality RMNCAH+N services are formidable. Greater attention to availability, quality and coordination of RMNCAH+N services, coupled with investments in health workforce development and supply management are needed to maintain access to life-saving services and mitigate longer term impacts on maternal and child health. Lessons learned from Yemen as to how to address ongoing primary health care needs during massive epidemics in conflict settings, particularly for women and children, will be important to support other countries faced with similar crises.

Keywords:
Yemen, humanitarian, maternal, newborn, child, reproductive heal

9 - Abstracts (Day 2, November 15th, 2021 Session 1)

Maternal, Child, and Reproductive Health

Abstract Code: 2021-FWBD-613
Presenter Name: Maryam Zahid
Country: Pakistan
Presenter Email: maryamzahid343@gmail.com
Author: Dr. Maryam Zahid

Title: Effect of Artificial Intelligence on Nutritional Status of Children Post Cardiac Surgery: A Randomized Controlled Trial

Background:
Malnutrition is the most common problem in congenital heart diseases patients. Health based mobile applications play an important role in planning and tracking of diet for better nutritional status.

Purpose:
To assess the effect of artificial intelligence on nutritional status of children post cardiac surgery in comparison to usual care group and to assess usefulness of diet related mobile application in comparison to usual care group.

Methodology:
This is a two arm randomized controlled trial that was conducted at a Tertiary Care Hospital, Rawalpindi. The study duration was 6 months from February 2021 till July 2021. Sample size was calculated to be 88. Intervention group was given a diet related mobile application and usual care group was handed a nutritional pamphlet on discharge. Study was registered on clinicaltrial.gov website with trial identity number NCT04782635.

Results:
The mean weight of all participants was 15 ± 5.7 kg at the time of discharge whereas at the end of 8th week the mean weight of the participants in usual care group was 16.5 ± 7.2 kg and intervention group was 17.1 ± 5 kg. Average calories consumed by usual care group was 972 ± 252 kcal and 1000.75 ± 210 kcal by intervention group after 8 weeks of discharge. At the end of intervention preferred diet planning tool for 79% of the participants was mobile application. At 8th week 80% think that the mobile application language was understandable, 79% of the participants think nutritional goal setting is a useful feature in mobile application and 55% of the participants think the recipes in the application were useful.

Conclusions:
The study showed strength for the future of scalable modern technology for self-nutrition monitoring. There was slight increase in the weight and nutritional intake of both groups as interventions period was limited.

Keywords:
Artificial intelligence, diet related mobile application, nutritional status, children post cardiac surgery, randomized control trial.

9 - Abstracts (Day 2, November 15th, 2021 Session 1)

Maternal, Child, and Reproductive Health

Abstract Code: 2021-OTH-620
Presenter Name: Samah T. A. Elamassie
Country: Palestine
Presenter Email: dr.samahtaysier@gmail.com
Author: Dr. Samah T. A. Elamassie

Title: Triggers Factors of Child Marriage in the Gaza Strip: Cross- Sectional Study

Background:
Marriage is about life long relationship it is about commitment, communication and be a good manager to any conflict. So, being married in a healthy status, in proper age, is a worthwhile goal. The prevalence of child marriage in the Gaza Strip was 23.7% . This figure reflects the large huge bulk of early marriage among girls, still many less than African countries.

Purpose:
The study explores the triggers factors for child marriage in the Gaza Strip form their perceptions, in order to help in decreasing this phenomenon.

Methodology:
The study was done in the Gaza Strip by face-to-face interview with 406 participants whose ages at the time of data collection were less than 25 and their ages at time of marriage were less than 18 year. The sample was selected through random -a multistage sampling approach- as cross-sectional manner. A mix of self-constructed and standardized tools were used and the response rate was high (99.5%). The data were analysed using the SPSS.

Results:
Findings showed that the median parents (of the early married) family size was 9, their parents have poor education level, and 73.6% were receiving social assistance. The study showed that the most commonly reported drivers for child marriage were family norms (82.5%), community norms (73.9%), religious beliefs (53%) and consanguinity (52.5%). Less frequently reported triggers included poor economic conditions (36.2%), having many sisters in the family (34.7%), low school achievement (25.1%) and previous exposure to violence (14.3%). We found that the father was the main source of force for their marriage. Also the study found that level of education could protect the female from early marriage, and those who had poor achievement at schools were significantly married earlier than those whose school achievements were better.

Conclusions:
The study concluded that child marriage is driven mainly by cultural and family norms.

Keywords:
Child marriage, triggers, perception.

9 - Abstracts (Day 2, November 15th, 2021 Session 1)

Maternal, Child, and Reproductive Health

Abstract Code: 2021-FWBD-657
Presenter Name: Sami Al-Haidari
Country: Yemen
Presenter Email: sami.alhaidari@hotmail.com
Authors: Dr. Mohammed Al-Amad, Dr. Othman Bahashwan, Dr. Walid Al Murisi, Prof. Abdulsalam Al-Mekhlafi, Prof. Abdulwahed Al Serouri, Prof. Ahmed Thabit, Prof. Hassan Al-Shamahi, Prof. Mohammed Mahdy

Title: Intestinal Schistosomiasis among Schoolchildren in Sana'a Governorate, Yemen, 2018: Prevalence, Associated Factors and its Effect on Nutritional Status and Anemia

Background:
Intestinal schistosomiasis is a neglected tropical disease, causing morbidity and mortality in tropical and subtropical countries. Despite the frequent implementation of mass drug administration with praziquantel, the reinfection with Schistosoma mansoni is still common in Yemen.

Purpose:
To determine prevalence and risk factors of intestinal schistosomiasis and investigate its impact on nutritional status and anemia among schoolchildren in the rural areas of Sana'a Governorate, Yemen.

Methodology:
A cross-sectional study was conducted in 2018. A multistage sampling approach was used for selecting schoolchildren from two districts. A standard questionnaire was used to collect data. Microscopic examination of Kato-Katz fecal smear was used to identification and quantification. Hemoglobin concentration and anthropometric measurements were estimated. Multivariable analysis using binary logistic regression model was used to calculate the adjusted odds ratio (AOR).

Results:
A total of 445 schoolchildren aged 5 – 15 years old was enrolled, with a mean of 10 ± 2.54. The prevalence of S. mansoni was 18.0%. Wasting, stunting, anemia and underweight were observed in 25%, 46%, 32%, and 27% of schoolchildren. Households without tap water (AOR = 3.5, P = 0.009), uneducated mothers (AOR = 3.2, P = 0.038) and Al-Haimah Al-Dakheliah District (AOR = 31.0, P = 0.001) were identified risk factors of S. mansoni. The early adolescent females were significantly more stunted (AOR = 2.4, P = 0.006). The early adolescent schoolchildren from families with low wealth (AOR = 4.1, P = 0.005) and moderate wealth (AOR = 3.0, P = 0.015) and were significantly more wasted.

Conclusions:
Schoolchildren are still at high risk of S. mansoni, which is an independent predictor of stunting. The early adolescent schoolchildren from poor families are at high risk of being wasted. Implementation of control measures to combat schistosomiasis and integrated diseases control program to improve the health status of schoolchildren was recommended.

Keywords:
Schistosomiasis mansoni, , Anemia, National Yemen.

10 - Abstracts (Day 2, November 15th, 2021 Session 2)

COVID-19

Abstract Code: 2021-EOH-702
Presenter Name: Alaa AlJanabi
Country: Iraq
Presenter Email: Alaa.Hussein1300a@comed.uobaghdad.edu.iq
Authors: Dr. Maher Kadhum, Prof. Faris Lami

Title: The Pattern of Comorbidities Among COVID-19 Patients and their Impact on the Outcome in Babel Governorate, Iraq, 2020

Background:
The presence of comorbidity poses a major clinical challenge in the care and treatment of COVID-19 patients.

Purpose:
To evaluate the effects of common comorbidities on the severity, outcome, and length of stay in the hospital of COVID-19 patients, Babel Governorate, Iraq, 2020.

Methodology:
All the laboratory-confirmed cases in the two COVID-19 hospitals in Babel governorates during March through September 2020 were included. A form was developed to compile sociodemographic data, clinical presentation and severity, comorbidities, length of stay at the hospital, and the case fatality rate.

Results:
The total number of patients was 2574; 1581 (61.4%) were males. The mean age was 48.7 (±16.4) years. The severe cases were 1212 (47.1%), and critical cases were 489 (19.0%). The patients with no comorbidity were 1543 (59.9%); 536 (20.9%) had one comorbidity, and 495 (19.2%) had two or more comorbidities. The most prevalent comorbidity was Diabetes Mellitus (25.0%), followed by hypertension (23.4%). The proportion of the severe/critical cases were 84.0% among the patients with comorbidity compared to 54.1% among the patients free from comorbid illnesses (P<0.001). Around 12% of patients with comorbidity had a mean hospitalization time >2 weeks compared to 8.0% among the patients with no comorbidity (P<0.001). The case-fatality ratio was 26.4% among patients with comorbidities compared to 10.6% among the patients free from comorbidity (P<0.001).

Conclusions:
Comorbid illnesses are a significant predictor of serious in-hospital course and fatal outcomes of COVID-19 patients. Those patients must undertake vigilant preventive measures and should have the priority to get the COVID-19 vaccine.

Keywords:
Comorbidities, COVID-19, Iraq, Outcome

10 - Abstracts (Day 2, November 15th, 2021 Session 2)

COVID-19

Abstract Code: 2021-EOH-748
Presenter Name: Asim Al Manji
Country: Saudi Arabia
Presenter Email: asalminji@gmail.com
Authors: Dr. Khalid Algaali

Title: The First Cluster of COVID-19 Cases in the Sultanate of Oman in Muttrah District, 2020

Background:
In Oman, the first cluster of COVID-19 cases was reported in Muttrah District, Muscat Province on 15 March 2020. Mutrah, with a population of 269,507 (76% expatriates), has a major seaport and a crowded traditional market. Both of these represent opportunities for COVID-19 importation and spread.

Purpose:
We describe the epidemiologic and clinical features and interventions for the first COVID-19 cases confirmed in Muttrah between 15th March-15th July 2020.

Methodology:
We identified cases from health centers and outreach teams. Demographic, clinical, and epidemiological data were collected by using specific form and entered into the e-notification system "Tarasud".

Results:
We identified 8,400 cases and 51 fatalities (case fatality rate= 0.61%) from 15 March to 15 July 2020. The mean age was 37 (±12.3) years; 85.6% were males. The most common symptoms were fever (48%), cough (34%), and sore throat (24%). A total of 17,202 tests were performed with 48.8% positivity rate. 77.5% of cases were non-Omani workers in the local market and seaport. These patients had low socioeconomic levels and often lived-in crowded housing. The majority of the patients were Indians (35%) and Bengalis (33%). Community education, lockdowns, mass testing, and isolation of confirmed cases were applied to control the outbreak.

Conclusions:
Most of the early cases occurred among male migrants who worked at the old market or the seaport. They lived and worked in crowded conditions, with contact with travellers, which may have contributed to the spread of COVID-19 in Oman. Initially, they had lower accessibility to health care and awareness messaging due to financial and lingual problems. The large-scale implemented interventions have slowed the disease spread but could have been optimized by tailoring to the highest risk groups.

Keywords:
SARS-CoV-2, COVID-19, pandemics, disease outbreaks, Muscat and Oman

10 - Abstracts (Day 2, November 15th, 2021 Session 2)

COVID-19

Abstract Code: 2021-EOH-747
Presenter Name: Dr. Ghaith Owies
Country: Jordan
Presenter Email: dr_ghaith99@yahoo.com
Authors: Dr. Ghaith Owies, Dr. Ghazi Sharkas, Dr. Ashraf Aqel, Dr. Majed Asad, Dr. Refqi Mahmoud, Zainab Ghafari.

Title: Clinical and Epidemiological Features of COVID-19 Deaths in Jordan

Background:
The uncontrolled spread of the COVID-19 around the world have caused many morbidities and fatalities. Susceptibility to severe outcomes differed among the population, but it was noticed to increase with age, obesity, presence of chronic diseases or during pregnancy.

Purpose:
To describe the demographic and clinical features of COVID-19 deaths in Jordan.

Methodology:
This was a descriptive study that used the national dashboard of COVID-19 available in the Jordanian Ministry of Health. The data was obtained from the multi-sectorial health system in Jordan and included all deaths from November the 11th of 2020 till April the 1st of 2021. Data collected represented both hospitalized and non-hospitalized death (Forensic medicine). Epi Info was used for analysis.

Results:
There were 5449 COVID-19 deaths reported during the period of the study. Findings showed more male deaths (ratio was 1.67 to 1) and more deaths among those 60 years and older (ratio was 3.02 to 1). Most COVID-19 deaths were Jordanian (94.4%) followed by Syrians (2.3%). Moreover, Amman had the highest number of COVID-19 deaths (53%) and Ajloun had the highest rate which was 89 deaths/100000. The mortality rate among was 18.79% hospitalized COVID-19 patients in all sectors and the university sector reported the highest mortality rate among hospitalized COVID-19 patients (26.69%). The mean hospitalization period for COVID-19 deaths was 9 days while the median was 7 days. Most COVID-19 deaths were among people with co – morbidities (n= 4840 or 88.82%). Hypertension presented 3924 deaths or 72% of these co – morbidities, followed by diabetes mellitus (n=3319 or 60.9%), then cardiovascular disease (n=1683 or 30.9%).

Conclusions:
Deaths varied among regions in Jordan and university hospitals reported the highest mortality rate. In addition, patients with co – morbidities should gain special attention because of their higher possibility for severe consequences and death.

Keywords:
Jordan, Fatality, Co – morbidities, Health sectors.

10 - Abstracts (Day 2, November 15th, 2021 Session 2)

COVID-19

Abstract Code: 2021-EOH-647
Presenter Name: Hassan Haridi
Country: Saudi Arabia
Presenter Email: hassankasim@hotmail.com
Authors: Dr. Abbas Al-Makramy, Dr. Ali Sweedan, Dr. Fahd Beharith, Dr. Mashhour Qannnas, Dr. Saied Qenawi, Ms. Ahlam Al-Qahtani

Title: Preparedness, Risk Perception, Concerns and Risk Acceptance Among Hospital Healthcare Workers (HCWs) in Facing the Emerging New Corona Virus (COVID-19) Outbreak in Najran Hospitals, Saudi Arabia at the Early Phase of the Pandemic

Background:
Coronavirus disease 2019 (COVID-19) pandemic extended to reach most countries in the globe during few months. Preparedness of healthcare institutions and healthcare workers are crucial for applying effective prevention and control measures.

Purpose:
This study aimed to assess HCWs and institutional preparedness in facing the new emerging corona virus (COVID-19) infection at the early phase of the pandemic, and to explore HCWs risk perception, concern and risk acceptance.

Methodology:
A cross-sectional survey was conducted among HCWs in Najran main hospitals, at the early phase of the pandemic, during March-April, 2020.

Results:
Overall, 563 HCWs participated in the survey. Among them, 65.8% attended training program/s for COVID-19 infection, 97.4% reported reading the official circulars regarding COVID-19 infection, 97.1% received basic infection control training, 98.9% checked for best fitted size of N95 mask, while 89.4% were influenza vaccinated. Of the participants, 82.6% self-reported having sufficient knowledge about COVID-19 pandemic, 92.9% stated that they understand the risk of COVID-19 infection, 82.0% being confident of protecting themselves and their patients against COVID-19 infection, while, 83.2% accepted the risk of infection being a part of their job. Of the participants, 70.8% agreed that institutional precautionary measures for COVID-19 in the workplace are sufficient and 71.6% reported that all PPE are always available in the workplace. Of the participants, 79.0%, 35.2%, 64.2% felt that they are, their families and Najran community are at high risk of getting COVID-19 infection respectively and 54.7% and 55.1% were concerned about their personal and family health, respectively.

Conclusions:
Findings revealed good knowledge about COVID-19 pandemic among HCWs in Najran hospitals. Participants, appreciated important aspects of institutional preparedness. Experience gained from the previous MERS-CoV outbreak may be explain good knowledge, risk acceptance, self-efficacy and good and rapid institutional preparedness at the early stage of the pandemic.

Keywords:
COVID-19; Knowledge; Concern; Healthcare Workers; Institutional preparedness; Saudi Arabia.

10 - Abstracts (Day 2, November 15th, 2021 Session 2)

COVID-19

Abstract Code: 2021-EOH-876
Presenter Name: Sharmin Chowdhury
Country: Bangladesh
Presenter Email: Sharminchowdhury@cvasu.ac.bd
Author: Dr. Jahan Ara

Title: Seroprevalence and Titer of Anti-SARS-CoV-2 Antibody (IgG) in Health Workers, Non-COVID-19 Patients and Garments Workers of Chattogram City, Bangladesh

Background:
Monitoring the prevalence and titer of anti-SARS-CoV-2 antibody over different phases of the pandemic and human interventions is important to anticipate its trends and design an efficient health response.

Purpose:
The present study was conducted to estimate the seroprevalence of anti-SARS-CoV-2 antibody (IgG) and its association with different explanatory variables. Further, antibody was quantified to assess the increasing or decreasing trend over different time periods.

Methodology:
This cross-sectional study observed health workers - doctor, nurse, hospital staff, in- and outpatients (non-COVID-19) and garments workers of Chattogram city (N=748) from randomly selected four government and private hospitals and two garment factories. Study subjects were included into the study upon written consent followed by fulfilling certain inclusion criteria. Venous blood was collected following standard aseptic method. Qualitative and quantitative ELISA was used for identification and quantification of antibody (IgG) in serum samples. Descriptive, univariable and multivariable statistical analysis was performed using STATA-13.

Results:
Overall seroprevalence was estimated as 66.58% (95% CI: 63%-69%). Seroprevalence among health workers, in- and outpatients and garments workers were 68%, 80% and 50%, respectively. Seroprevalence was 44% in unvaccinated population while it was significantly (p-value <0.001) higher in population received 1st dose (61%) and both (1st and 2nd) doses of vaccine (99%). Mean titer of antibody was estimated as 255 DU/ml and 159 DU/ml in population with 1st and both doses of vaccine, respectively compared to 53 DU/ml of nonvaccinated group. A decreasing trend in the quantity of antibody with increasing time after vaccination was observed.

Conclusions:
Seroprevalence and mean antibody titer varied according to different factors in this study. Second dose of vaccine significantly increased the seroprevalence and titer which decreased to a certain level over time. This study shows the role of vaccine towards antibody production.

Keywords:
Seroprevalence, anti-SARS-CoV-2 antibody, antibody titer, IgG.

10 - Abstracts (Day 2, November 15th, 2021 Session 2)

COVID-19

Abstract Code: 2021-EOH-781
Presenter Name: Sofia Azrib
Country: Morocco
Presenter Email: sofia.azrib@gmail.com
Author: Dr. Sofia Azrib

Title: Descriptive Epidemiology of Coronavirus 2019 in Casablanca-Settat region, Morocco, March 2020 – September 2021

Background:
Coronavirus disease 2019 (COVID-19) has caused unprecedented global morbidity and mortality. Casablanca-Settat region recorded the first case of COVID-19 on 2nd March 2020 and has faced three epidemic "waves" of COVID-19 since then. The Ministry of Health with the support of technical partners responded quickly to contain the outbreak.

Purpose:
The objective of this study is to describe the epidemiology of COVID-19 in Casablanca-Settat Region with a view of generating evidence to enhance planning and response strategies.

Methodology:
A regional surveillance dataset between 2nd March 2020 and 28th September 2021 was retrospectively analysed, with confirmatory testing for COVID-19 done by real-time polymerase chain reaction (RT-PCR). Field investigations were conducted and contact tracing and follow up were done. All confirmed cases were identified, line-listed and analyzed. Various indicators were monitored to improve interpretation of surveillance data.

Results:
A total of 1,913,954 persons had complete records of RT-PCR test across 16 provinces and prefectures, 346180 (18 %) of whom were confirmed COVID-19 cases . The overall cumulative incidence and case fatality were 4673 per 100,000 population and 1.1%, respectively. A total of 339,478 (98%) recovered cases were recorded. The highest proportion of COVID-19 cases and deaths were recorded in persons aged 25–45 years (40.2%) and 65–85 years (41.6%), respectively. Moreover, females accounted for a higher proportion of confirmed cases (54.8%). About 66% of confirmed COVID-19 cases were asymptomatic at diagnosis. The first wave was characterized by detection of cases imported from Europe, followed by notifications of sporadic cases, clusters. The last wave brought considerable morbidity and mortality, surpassing the cumulative case counts and fatalities from the earlier waves.

Conclusions:
Important lessons were learned from each wave and across waves. Surveillance activities such as contact tracing and follow- up, and active case search were useful in early case detection and control of the outbreak.

Keywords:
COVID-19; Epidemiology ; Casablanca Settat Region, ; Morroco

10 - Abstracts (Day 2, November 15th, 2021 Session 2)

Vector Borne Diseases

Abstract Code: 2021-EOH-641
Presenter Name: Khaled Al-Jamrah
Country: Yemen
Presenter Email: khjamrah2010@gmail.com
Authors: Dr. Abdulkareem Nassar, Dr. Mohammed Al Amad, Dr. Areef Shamsan, Dr. Ibraheem Mohathab

Title: Dengue Fever Outbreak in Al-Garrahi District, Al-Hudaydah governorate, Yemen, 2019

Background:
Dengue fever (DF) has reemerged in Yemen with higher frequency during the last years. On Nov 6, 2019, an increased number of suspected DF in Al-Garrahi district was reported. On Nov 7, 2019, team sent to investigate.

Purpose:
To confirm the existence of outbreak, describe the outbreak characteristics, and to recommend suitable intervention for control.

Methodology:
A descriptive study was conducted. WHO case definition was used to identify patients. Active search from house to house along with entomological investigation and health education were conducted. A line-list was used to collect data. Blood specimens were collected and tested by ELISA for dengue IgM. Frequency, percentage and rates were calculated and population from central statistical organization was used.

Results:
A total of 2067 cases met the case definition. Of those, 51% were males and 32% were <10 years old. All patients complained from fever, headache and arthralgia (100%) followed by myalgia and retro orbital pain (67% and 39%) respectively. The first case-patient was in week 41 and reached the peak with 1058 patients in week 46. The overall attack rate was 16/1,000, significantly higher among patients with 10-<50 and ≥ 50-year-old compared with patients <10 years old (17 and 19 /1000 vs 14 /1000, P value < 0.001). Out of 20 tested blood samples, 12 (60%) were IgM positive. House index was 70%, the container index 50%, and the Breteau index was 140. Vector control measures with community participation were intensified in week 46 and patient-cases decreased to 140 in week 48.

Conclusions:
Dengue outbreak was confirmed in Al-Garrahi district. Spread of infection facilitated by storing water and presence of indoor larvae. The findings emphasize the importance of health awareness and community participation for containing DF outbreak.

Keywords:
Dengue fever, Outbreak investigation, Yemen, FETP

10 - Abstracts (Day 2, November 15th, 2021 Session 2)

Vector Borne Diseases

Abstract Code: 2021-EOH-716
Presenter Name: Khalid Alsaadi
Country: Oman
Presenter Email: ka39saadi@gmail.com
Author: Dr. Eman Elsayed

Title: Crimean Congo Hemorrhagic Fever Outbreak in North Part of Oman in August 2019 -A Case Series Study

Background:
Crimean-Congo hemorrhagic fever (CCHF) is a viral zoonotic tick-borne disease with a high fatality rate reported in many countries. The first patient of CCHF in Oman was detected in 1995. Between 17th and 23th August 2019 (Eid Adha festival) Directorate of Disease Surveillance and Control received reports of 4 patients of CCHF from different areas in North Oman Region.

Purpose:
To identify CCHF patients, identify the source for each patient with mode of transmission and recommend preventive measures for future outbreaks.

Methodology:
After meeting with public health team, we prepared for field visit at the same day of notification along with teams from Ministry of Agriculture, Fisheries and Municipality. A case series study was conducted to gather information using semi structured questionnaire between 17th August and 23th August 2019 in North Oman Region. The case definition was implicated according to Omani Ministry of Health guideline.

Results:
The results showed that all patients are males (3 are Omanis) with age ranged from 40 to 55 years. Three patients were working in slaughterhouses while all patients had direct contact with fresh sheeps tissues. The maximum incubation period was 14 days. The most frequent symptoms were fever and gastrointestinal symptoms with case fatality rate of 25%. The patient who passed away presented late with bleeding symptoms and coagulopathy.

Conclusions:
Based on the symptoms, signs, lab investigations and incubation period the causative agent was most likely CCHF Virus and the source of the outbreak was due to infected imported sheeps through direct contact with infected biological tissues. It is mandatory to test and flag all the imported sheeps on the main gate of the 3 ports in the North Region of Oman.

Keywords:
CCHF, outbreak, slaughterhouse, sheeps, Eid, patient.

10 - Abstracts (Day 2, November 15th, 2021 Session 2)

Vector Borne Diseases

Abstract Code: 2021-EOH-770
Presenter Name: Mohamed Gouda
Country: Egypt
Presenter Email: mohamed.n.gouda@gmail.com
Author: Dr. Mohamed Gouda

Title: Mixed Outbreak of Falciparum and Vivax Malaria and Dengue Fever Among the Egyptian Five-a-side Ball Team Returning from Nigeria After Participation in the African Cup of Nations, Egypt July 2021

Background:
On 28 July 2021, Port-Said health directorate was reported of a team member of the Egyptian Five-a-side ball team who was admitted to fever hospital . The patient has recently returned from Nigeria with his teammates after participation in the African Cup of Nations. On asking the patient of other cases he informed of one who started to feel sick in Nigeria and quarantined in Abasia fever hospital and other two who are currently at their homes in Port Said.

Purpose:
To confirm an outbreak, identify possible causes and implement necessary control measures.

Methodology:
All 26 team members were conatcted. Active case finding among them and their contacts was performed using sensitive case definition. Case was defined as anyone from the Egyptian Five-a-side ball team returned from Nigeria or their contacts who complain of sudden onset of fever, malaise and/or respiratory or gastrointestinal symptoms. Symptomatic patients were swabbed for SARS-CoV2 by RT-PCR. Blood samples were collected for malaria film and IgM for dengue testing.

Results:
Out of 26 team member, 7 compalined of symtoms giving attack rate (26.9%). Their mean age was 37.1±8.6, all of them were males. Of the seven cases, 6 (85.7%) were from Portsaid, their symptoms ranged from fever (42.9%) malaise,(100%), headache (57.1%), nausea (42.9%), and one had delirium. Laboratory testing confirmed 4 cases with malaria Faciparam including one positive for COVID-19. The blood film of two patients showed Falciparam and Vivax. One patient positive for SARS-CoV-2 and Dengue. All patients recovered. Patients did not receive malaria prophylaxis before travelling. Vector survey was performed and mosquito spry and larvae destruction near the residential areas.

Conclusions:
Defective malaria preventive measures with no prophylaxis caused outbreak which was controlled through early detection, isolation, treatment, entomological and environmental preventive measures. Ensure that all sportive teams travelling to endemic areas should apply protective measures.

Keywords:
Covid-19 , Falciparum , vivax, mixed outbreak, malaria , dengue fever.

10 - Abstracts (Day 2, November 15th, 2021 Session 2)

Vector Borne Diseases

Abstract Code: 2021-EOH-667
Presenter Name: Prasoon Sheoran
Country: Other
Presenter Email: prasoonsheoran12@gmail.com
Authors: Dr. Chandrakant Moghe, Dr. CS Aggarwal, Dr. Sachin Sharma, Dr. TG Thoma

Title: Malaria Outbreak Investigation in a Tribal Area of Pratapgarh District, Rajasthan, India, 2016

Background:
Although India aims for malaria elimination by 2024, there were 1.1 million malaria cases with 561 deaths in 2014. Thorough outbreak investigations can identify gaps and target local interventions to help achieve elimination goals. On 22 September 2016, a malaria outbreak was reported from Pal, District Pratapgarh in Rajasthan.

Purpose:
We investigated to describe the outbreak and provide recommendations.

Methodology:
We defined a suspect patient of malaria as acute febrile illness in a resident of Pal sub-centre area between 1 September-31 October 2016 and a confirmed patient as smear-positive suspect case. We searched for patients by reviewing outpatient registers of health facilities. We evaluated all suspect patients by peripheral blood smear. We also conducted an entomological survey and environmental assessment.

Results:
We identified 639 patients (441 suspect, 198 confirmed). Among confirmed patients, 45% were female; median age was 12 years (3 month - 75 years). Attack rate was 3% in Pal sub-centre but highest in Balaliya village (9%). Among 198 confirmed cases, 177 (89%) were positive for Plasmodium falciparum. There was 615 mm rainfall in August 2016 (53% above average from 2008-2015). Water accumulation was found around Jakham river bank and hand-pumps. Among 12 sites surveyed, three were positive for adult Anopheles culicifacies. There was no residual spray in last three years, and Pal sub-centre health facility was vacant for six months before the outbreak.

Conclusions:
This was a malaria outbreak of mostly P.falciparum with limited health resources, lack of spraying and heavy rainfall likely contributing. We recommend providing a permanent staff at the sub-centre health facility, indoor residual spray and co-ordination with village leaders to prevent water accumulation around hand-pumps while using larvicides at other water accumulation sites.

Keywords:
Malaria, Pal, Anopheles culicifacies, P falciparum.

Seventh EMPHNET Regional Conference
Amman, Jordan

10 - Abstracts (Day 2, November 15th, 2021 Session 2)

Vector Borne Diseases

Abstract Code: 2021-EOH-759
Presenter Name: Saba Ajam
Country: Pakistan
Presenter Email: fvopeshawar@hotmail.com
Author: Dr. Adnan Tariq

Title: Risk Factors Associated with Dengue Fever in District Khyber, Khyber Pakhtunkhwa, Pakistan from 2nd September to 16th November 2019: A Case Control Study

Background:
Dengue is a vector-borne disease which is endemic in Pakistan and Khyber Pakhtunkhwa as well. Total 300 confirmed dengue cases were reported in Khyber district in 2019, where prompt response was initiated by Disease Surveillance and Response Unit (DSRU). Subsequently, a study was planned to identify the risk factors associated with dengue fever and propose recommendation for contaminant of disease.

Purpose:
To assess the risk factors associated with dengue fever in district Khyber, give input to further strengthen the existing system, andsuggest recommendation for Dengue prevention in district Khyber.

Methodology:
A case control study was conducted in Khyber district from 2nd September to 20th November 2019. Cases were enrolled from health care facilities on a pre-defined cases definition where clinical signs and symptoms as well as laboratory confirmed NS1 results were included. Controls were taken from the community with a ratio of 1:2. Data was collected on a pre-tested questionnaire by face to face interviews.

Results:
A total of 300 cases were enrolled. Among all cases, 87% (n=263/300) were males. Non usage of bed nets 45% (n=269), OR 15 (CI 9.9 to 24.07, P <0.001), non-usage of mosquito repellant 39% (n=236/601) OR 1.7 (CI 1.1 to 2.4, P<0.001), presence of positive patients in neighbors 39% (n=233), OR 16.6 (CI 11.11 to 24.93, P<0.001), subjects infected with dengue fever in a combine family 35% (n=213), OR 1.47 (CI 1.04 to 2.00, P <0.001), and sleeping outdoors 40% (n=241), OR of 3.32 (CI 2.3 to 4.7, P<0.001), were found to be significantly associated with dengue fever.

Conclusions:
Dengue is a preventable disease and can be controlled by proper usage of bed nets, mosquito repellants, modifying sleeping habits, and improving family structure. Training of the health staff on dengue management and community awareness is recommended.

Keywords:
Bed nets, Dengue, District Khyber, Disease Surveillance & Response Unit, Risk factor.

10 - Abstracts (Day 2, November 15th, 2021 Session 2)

Vector Borne Diseases

Abstract Code: 2021-EOH-634
Presenter Name: Uzma Hafeez
Country: Pakistan
Presenter Email: druzmahafeez@gmail.com
Author: Dr. Syed Nadeem Ur Rehman

Title: Outbreak of Dengue Fever in Chattar Muzaffarabad: A Risk Factors Analysis - 2019

Background:
Dengue fever is an epidemic-prone disease which has become a global public health issue in recent decades. The Provincial Disease Surveillance & Response Unit reported an upsurge of Dengue fever cases in Chattar, Muzaffarabad since 7th September 2019.

Purpose:
An outbreak investigation was carried out to assess the magnitude of the problem, identify associated risk factors, and to suggest preventive and control measures.

Methodology:
A case was defined as "any resident of Chattar presenting with fever and any two of the symptoms: headache, retro-orbital pain, myalgia, arthralgia, nausea, vomiting, abdominal pain, bleeding, with positive Immune-chromatographic IgM/IgG rapid test or NS1Ag test between 1st September - 30th October 2019". For the case control study, equal number of age and sex matched controls were taken from neighborhood-households. A structured questionnaire was administered to collect information about demography and risk factors. Descriptive statistics and univariate analysis were carried out.

Results:
A total of 197 cases were identified with active home search in which 113 (57.45%) were males. The median age of cases was 24 years (range 9-67 years).The overall attack rate was 32.8/10,000 population. Age group 20-29 years was the most affected group (31.9%).The Epi-curve showed multiple peaks. The presence of larvae in and around houses and open over head water tanks for storage of water were associated with the illness, OR 4.8 (CI 3.1-7.4, P < 0.001) and OR 3.1 (CI 2.0-4.7, P < 0.001), respectively,whereas use of indoor residual spray, screening of doors and windows of houses,and use of mosquito repellents by the incumbents were found protective to dengue disease.

Conclusions:
Uncovered fresh water in and around houses were thepotential Aedes aegypti larvae breeding sites that caused the outbreak. Community awareness about dengue, appropriate vector control measures and effective dengue surveillance were taken successfully to control the outbreak and prevent future outbreaks.

Keywords:
dengue outbreak,uncovered water tanks, AJK

11 - Abstracts (Day 2, November 15th, 2021 Session 3)

Surveillance

Abstract Code: 2021-EOH-678
Presenter Name: Abass Jouny
Country: Lebanon
Presenter Email: jounyabass@gmail.com
Author: Mrs. Hawraa Swaidan

Title: COVID-19 Case Based Reporting Using DHIS2 Tool, Lebanon, 2020-2021

Background:
On February 21st, 2020, Lebanon reported the first COVID-19 case. Responding to that and to ending transmission, containment approach was adopted till July 2020. Thereafter, health strategy moved to mitigation. Online surveillance platforms, such as DHIS2, were important for decision makers and health professionals, for timely monitoring of the transmission level, and availability of epidemiological findings

Purpose:
DHIS2 platform offers an electronic platform for case reporting, analyzing data collected, and sharing data with stakeholders, for a real-time response.

Methodology:
DHIS2, the world's largest health information system, is used for COVID-19 cases in Lebanon. To avoid under-reporting, a program was designed for online reporting (android application for offline reporting), ensuring reachability at anytime. The program was prepared with training material, credentials for end-users and on-site training were delivered upon request. Once the health facility reports its cases' demographic information, the surveillance team accesses the case reported to complete stage 2 with the information gathered and proceed to stage 3 if fatal outcome. An excel import application was available as another way to import data from diverse health systems.

Results:
328 accounts were created for facilities performing PCR (188), adding to that surveillance teams (120), and decision makers (20). 2,770 accounts were created for municipalities for instant access to cases reported in parallel with IMPACT platform integrated with DHIS2 to read daily cases. Till September 2021, 621,155 cases were reported out of which 1.36% were travel related, with 8.7% duplicate rate, fetched using an SQL script. And 8,267 death cases were reported. Dashboards are created accordingly, with graphs generated using connections established between DHIS2 and R.

Conclusions:
In response to COVID-19, the need for online reporting surges, for timely monitoring of transmission indicators, sharing data with professionals and public, and building trust in the health system through transparency.

Keywords:
COVID-19, Lebanon, outbreak, surveillance, DHIS2

11 - Abstracts (Day 2, November 15th, 2021 Session 3)

Surveillance

Abstract Code: 2021-HIV/STI-729
Presenter Name: Abdul Shakour Karimi
Country: Afghanistan
Presenter Email: karimi.afetp@gmail.com
Author: Dr. Mir Islam Saeed

Title: Evaluation of Routine Immunization Coverage in 12-23 Months Children in Sarepol Province 2018: a Descriptive Cross-Sectional Study

Background:
Afghanistan is one of three countries in which polio is still endemic. AFP surveillance was adopted globally as a key strategy for monitoring the progress of the polio eradication initiative.

Purpose:
This study aims to evaluate different attributes of AFP surveillance systems in Sarepol province, identify strengths and weaknesess and formulate recommendations for its improvement.

Methodology:
Using CDC updated guidelines for evaluation of public health surveillance system, we conducted a desk review and approached health workers at public and private facilities as well as clinical, public health and managerial positions to evaluate the AFP surveillance system in Sarepol province in March 2019. This evaluation particularly focused on main attributes including simplicity, completeness, sensitivity, stability, and timeliness of the surveillance system.

Results:
The AFP surveillance system in Sarepol province detected 42 AFP cases in 2018 from all districts. It shows high sensitivity. As a whole 52% of the AFP cases were reported by medical doctors, 29% by nurses, 19% by community health workers and 3% by community. Timeliness requires improvement as delays in reporting were observed at regional level, as out of the 42 cases, 34 were delayed between 1-2 days (80. 95%), 3 cases stayed between 2-3 (7.14%), and 2 cases stayed 3-4 days (14.76%). All respondents asked reported they need training on AFP surveillance. Information generated by AFP surveillance were used at provincial level, and no cases are missing by the system which shows high level of sensitivity. No polio cases were confirmed after lab exams of AFP cases in Pakistan.

Conclusions:
The evaluation showed that simplicity, stability, acceptability, and knowledge about the system were good. AFP surveillance system is functional and working as a good strategy to eradicate polio in Afghanistan. The MoPH should focus on timeliness of the system. It could be done by engaging the community.

Keywords:
AFP, Surveillance System, Sarepul

Seventh EMPHNET Regional Conference
Amman, Jordan

11 - Abstracts (Day 2, November 15th, 2021 Session 3)

Surveillance

Abstract Code: 2021-OTH-694
Presenter Name: Abdulqawi Qaserh
Country: Yemen
Presenter Email: aabuhosam33@gmail.com
Author: Dr. Labiba Anam

Title: Role of RRT in Response to Outbreaks, Yemen, 2020

Background:
Yemen has been increasingly reporting public health emergencies e.g. Cholera. The Ministry of Public Health and population (MoPH&P) has put in place the Rapid Response Teams (RRTs) mechanism from national to district level to investigate and initiate response to public health emergencies. A RRT is a technical, multi-disciplinary team that is readily available for quick mobilization and deployment in case of emergencies.

Purpose:
This analysis aims to summarize the role of RRT in response to outbreaks, Yemen, 2020

Methodology:
Data was obtained from the electronic Diseases Early Warning System (eDEWS) in excel format covering the period from January- December 2020. Examples of data variables include governorates, districts, and number and place of health education sessions. Data was cleaned and analyzed using Excel 2013. Percent was used for qualitative data. Data was presented using tables, graphs, and maps.

Results:
A total of 39,451 field descent were done. Nearly half of the activities 18,565 (47%) were for outbreak investigation of many infectious disease: Cholera 9,030 (49%), SARI 1,949 (11%), Diphtheria 1,532 (8%), Measles 1,328 (7%), Malaria 1,012 (5%), Dengue Fever 1,008 (5%), Pertussis 803 (4%), Mumps 676 (4%), Chicken pox 583 (3%), AFP 482 (3%), Meningitis 162 (1%). About 1,747 (4%) supervision visits were implemented. Regarding health education, around 19,139 (49%) health education sessions were executed: Number of sessions at the health facilities was 3,419 (18%) and 157,20 (82%) were outside the health facilities e.g. schools and outdoors. Number of people who attended the health education sessions was 559,805.

Conclusions:
RRTs activities support the MOPH & P in reducing or 'slowing down' disease transmission as quickly as possible through many activities such as outbreak investigations and health educations. Therefore, there is a high need to continue supporting the RRTs financially and logistically by donors. In addition, governmental financial support to the RRTs is highly recommended to ensure the sustainability of the program.

Keywords:
RRT, Roles, activities, Yemen, 2020

11 - Abstracts (Day 2, November 15th, 2021 Session 3)

Surveillance

Abstract Code: 2021-OTH-624
Presenter Name: Ahmed AlMashaykhi
Country: Oman
Presenter Email: drahmed929@Gmail.com
Authors: Dr. Abdulaziz Saad ALMutari, Dr. Randa M. Nooh, Dr. Sami Said Almudarra, Dr. Khalid Masoud AlGhamdi, Dr. Naif Saud ALBudayri, Dr. Rabee Khalfan

Title: Pattern of Referral of Sick Omani Pilgrims from Omani Medical Mission During Hajj 2019

Background:
Annually, in the month of Dhul hijjah, over 2 million Muslims travel to Saudi Arabia to perform Hajj. Hajj is the biggest mass gathering globally, which creates a significant influence on Hajjes' health. The Omani medical mission is the official delegation from the Omani government to Saudi Arabia to serve the Omani hajjees regarding their health issues.

Purpose:
This study investigates the referral rate and pattern of diseases among hajjees referred by the Omani medical mission during Hajj 1440 H.

Methodology:
We conducted a cross-sectional study at the Omani Medical missions in Makkah, Madinah, Mina, and Arafat. Data was collected via a predesigned form. All Omani pilgrims presenting to the mission who were referred to local hospitals were included.

Results:
The total number of cases was 5000, of which 106 (2.1%) were referred to local hospitals (21.2 per 1000 hajjees). The most common causes of referral were cardiovascular diseases (23.6%), followed by gastrointestinal disease (17.9%) and trauma (16.9%). Males comprised 60.1%. Their mean age was 47.3 years (SD ±11.27), with the highest referrals in the 51-60 years age group (30%). Over half (55.7%) had co-morbidities. Patients' mean time to reach the clinic was 8.87 min (SD ±6.41), with 65% arriving in 5 min or less. The mean time needed to reach the hospital by ambulance was 11.39 min (SD ±6.6), with 36% arriving within 5 min. Of the referrals, 42% were admitted into hospital. Hospitalization was significantly higher among patients with chest pain (P-value < 0.0057), diabetics (P-value < 0.0001), and patients with Heart Disease (P-value = 0.013).

Conclusions:
The most common causes for referral of Hajjees from the Omani Medical Mission were cardiovascular diseases, gastrointestinal disease, and trauma. This information should assist the Omani government in planning their medical services in hajj season in future years.

Keywords:
hajj, Oman, referral, pilgrims

Seventh EMPHNET Regional Conference
Amman, Jordan

11 - Abstracts (Day 2, November 15th, 2021 Session 3)

Surveillance

Abstract Code: 2021-HIV/STI-761
Presenter Name: Elham Zeehrah
Country: Yemen
Presenter Email: drelhamabdullah@gmail.com
Authors: Dr. Ahmed Al-Sharaji, Mrs. Ghadah Al-Habob, Mr. Abdul Wahed Al-Serouri

Title: The Immunization Data Quality Assessment, Sana'a Capital, 2021

Background:
The Expanded Program of Immunization (EPI) aims to increase immunization coverage however, this cannot be achieved without efficient data management system and ensuring data quality.

Purpose:
Assess the quality of immunization data at Sana'a capital.

Methodology:
The WHO Data Quality Self-assessment (DQS) tools were used. Three randomly urban and the only rural districts (Bani-Al Hairth) at Sana'a capital were selected. From each district, one-third of the public health facilities (HF) that are providing EPI services were randomly selected. Accuracy Ratio (AR), Discrepancy Level (DL), completeness, and timeliness were calculated from tally sheets and reports for Bacillus Calmette Guerin (BCG), third dose of pentavalent (Penta-3), and the first dose of Measles and Rubella (MR-1) vaccines. The Quality Index (QI) was assessed for the five components (i.e. recording and reporting; archiving, demographics information, core output/analysis, and using data for action) through a pre-structured questionnaire.

Results:
While the overall AR and DL for BCG, Penta-3, and MR-1 showed an over-reporting at HF level, there was an over-reporting for BCG and Penta-3 but under-reporting for MR-1 at the district level. Regarding the QI, while recording and reporting achieved the highest overall score (90% and 96%), using data for action had the lowest score (61% and 78%) at the HF and district level respectively. While completeness and timeliness were 100% at all HF, both were inadequate at Al-Sabain and Bani-Al Hairth districts: 93% and 99%; and 75% and 83%, respectively.

Conclusions:
Findings showed that the quality of immunization data in Sana'a capital's HF and districts was inadequate with weaknesses in using data for action. Furthermore, completeness and timeliness found to be unsatisfactory at the rural and one of the urban districts. Ensuring data quality through strengthening EPI data management system should be prioritized. Larger scale and regular assessments of EPI data management system were recommended.

Keywords:
Accuracy ratio, Data Quality Self-assessment, Quality Index, health facilities

11 - Abstracts (Day 2, November 15th, 2021 Session 3)

Surveillance

Abstract Code: 2021-HIV/STI-836
Presenter Name: Omar Sharif Khan
Country: Pakistan
Presenter Email: dromarsharifkhan@gmail.com
Author: Dr. Syed Wasif Javed

Title: An Evaluation of Dengue Surveillance System in Khyber Pakhtunkhwa (KP) Province, Pakistan, 2020

Background:
To detect early outbreaks; installation and actualization of disease surveillance system is prerequisite. Prompt response is possible when robust surveillance system is in place.

Purpose:
This study aimed to document the current dengue surveillance system, gauge its performance, and provide recommendations to the stakeholders for its actualization and strengthening.

Methodology:
A cross sectional study was conducted. The evaluation was guided by updated Centre of Disease Control public health surveillance system guidelines for the year 2019. A structured questionnaire was designed and piloted to estimate simplicity, flexibility, acceptability and stability. Total sample size was 45 surveillance staff at provincial and district level. Dengue fever provincial data was analyzed to evaluate completeness, data quality, predictive positive value, sensitivity and representativeness. Field visits to districts were carried out to assess data flow and timeliness. Data analysis done by Epi-Info7.

Results:
Reporting rate ranged from 12/100,000 in 2017 to 21/100,000 in 2019, with a total number of 7641 reported cases in the province. The mean time of reporting cases was one day; ranging from zero to two days. Dengue surveillance system simplicity was 90% regarding structure and dataflow. Stability of the system was at 84% due to data backup. The system flexibility was 81% allowing addition and modification of variables. The average completeness of selected variables was 65%. About 59% of the staff interviewed considered the system acceptable. Data quality was suboptimal at 48%. The system is mainly representative of secondary and tertiary health care hospitals which was 40%, particularly inpatients. The system predictive positive value of dengue fever was 15% and sensitivity was 14% which were below par.

Conclusions:
An immediate, collaborative, multisector and transdisciplinary plan is needed to enhance reporting from all health facilities. Adequate government funding to improve data quality and ensure monitoring mechanism at all levels for prompt functionality of the surveillance system.

Keywords:
Evaluation; Dengue; Surveillance; Khyber Pakhtunkhwa

12 - Abstracts (Day 3, November 16th, 2021 Session 1)

COVID-19

Abstract Code: 2021-EOH-859
Presenter Name: Emna Mziou
Country: Tunisia
Presenter Email: dremnamziou@gmail.com
Authors: Dr. Aicha Hchaichi, Dr. Hajer Letaief, Dr. Leila Bouabid, Dr. Molka Osman, Dr. Mouna Safer, Dr. Sondes Darouiche, Dr. Sonia Dhaouadi, Dr. Souha Bougatef, Dr. Hamida BenSalah, Dr. Nawel ElMili, Dr. Nissaf Ben Alaya

Title: Covid-19 Epidemic Profile on the Tunisian Pediatric Population

Background:
After 18 months since the appearance of Covid-19 in Tunisia, it has been established that Covid does not spare the pediatric population. However, the first confirmed pediatric case of Covid-19 in Tunisia was reported in March 2020.

Purpose:
The objective of this study was to describe the epidemic profile of COVID-19 on the pediatric population in Tunisia.

Methodology:
We conducted a national prospective descriptive study spread over the period from March 02, 2020 to August 28, 2021. We included all pediatric cases, confirmed by RT-PCR or by antigenic-test reported to the Tunisian National Observatory of New and Emerging Diseases.

Results:
Up to August 28, 2021, Tunisia recorded a total of 38,652 pediatric cases confirmed by RT-PCR or by antigenic-test (7.7% of the total positive cases). The age group mostly affected by COVID-19 was 15 to 18 years with a sex ratio of about 1. The daily number of confirmed pediatric cases at the beginning of the epidemic did not exceed 10 cases per day until August 2020. The curve then showed, in the period from August to December 2020, a platter phase, corresponding to 60 pediatric cases per day. From January 2021, an upward trend was recorded with a peak of 150 pediatric cases confirmed on January 8, 2021, corresponding to the 2nd outbreak of the 1st wave that the country experienced due mainly to the wild variant of the virus. The curve decreased slowly until March 2021 and then recorded a continuous recrudescence from May 2021 with a peak of 616 pediatric cases per day, confirmed on July 8, 2021, corresponding to 7.2% of confirmed COVIDd-19 cases on the same day.

Conclusions:
Relatively spared during the first wave, the pediatric population has been significantly affected following the emergence of the COVID-19 mutant viruses. Should we reconsider COVID-vaccination of the pediatric population?

Keywords:
COVID-19; pediatric; Tunisia;

12 - Abstracts (Day 3, November 16th, 2021 Session 1)

COVID-19

Abstract Code: 2021-EOH-721
Presenter Name: Nameer Ali
Country: Iraq
Presenter Email: Nameer.kareem1300a@comed.uobaghdad.edu.iq
Author: Dr. Nameer Ali

Title: Sex-based Variations in Clinical Manifestations, Co-morbidities, and Outcome of COVID-19 Patients in Baghdad, Iraq, 2020

Background:
A higher incidence of COVID-19 in males has been widely reported. However, whether clinical manifestations, comorbidities, severity, and outcome differed between males and females remains an area of active investigation.

Purpose:
To compare the clinical features, comorbidities, severity, and outcome between male and female COVID-19 patients, Baghdad, Iraq, 2020.

Methodology:
We performed a records-based cross-sectional study by extracting sociodemographic, clinical manifestations, severity, and outcome data from the records of COVID- 19 patients who tested positive for SARS-CoV-2 using the RT-PCR test and admitted to two COVID-19 hospitals (AlKarkh and AlFurat General Hospitals) in Baghdad, Iraq between June and August 2020.

Results:
We reviewed a total of 2111 patient records with a history of COVID-19, and 1175 patients (55.7%) of them were males. We found that the following manifestations were significantly more common in females than males: respiratory symptoms (90.5% vs 87.6%; p=0.034), sore throat (14.0% vs 10.7%, P=0.023), and gastrointestinal manifestations (11.5% vs 7.2%; p=0.002). No significant difference was noted for fever, nasal congestion, conjunctival congestion, headache, and musculoskeletal manifestations. Generally, female patients had a significantly higher proportion of comorbidities than males (42.7% vs 36.0%; P=0.002). The proportion of severe and critical cases was not significantly different between males and females (P>0.05). The mean time from diagnosis to the outcome was significantly longer in females (P=.034), but the duration of the hospital stay showed no significant difference between males and females (P>0.05). Finally, the case fatality ratio was higher in males (16.1%) than females (13.2%) (P=0.022).

Conclusions:
Patients' sex affects the clinical course and outcome of COVID-19 patients. Male patients may need more attention considering the higher case fatality ratio.

Keywords:
COVID-19; Iraq; Sex, case fatality ratio, comorbidity

12 - Abstracts (Day 3, November 16th, 2021 Session 1)

COVID-19

Abstract Code: 2021-OTH-768
Presenter Name: Walaa Attia
Country: Egypt
Presenter Email: walaa_alim111@hotmail.com
Authors: Dr. Alaa Eid, Dr. Hanaa Abu Elsood, Dr. Sahar Samy, Dr. Salma Afifi

Title: Indicator-Based Surveillance and Event-Based Surveillance Complementary Role in Early Detection and Control of COVID-19 Outbreak in a Hypermarket – Egypt, April 2020

Background:
Indicator-based surveillance (IBS) and event-based surveillance (EBS) are complementary sources of information, and both contribute to the early warning for prompt response. Egypt National Surveillance (NEDSS) established in 2002 is an IBS that provides timely electronic-based clinical and epidemiologic data of patients with 41 infectious diseases including COVID-19, presenting to all governmental hospitals. While EBS, established in 2015, provides data of events with public health concern.

Purpose:
To describe the complementary role of both systems in early detection and response to cluster of COVID-19 patients. To give recommendations for improving this role.

Methodology:
Signal was first detected by EBS, it was filtered and verified using IBS database and communications with hypermarket management. Active case finding immediately started using sensitive case definition, data collected using line list, patients' and control measures started. All employees were swabbed and tested for SARS-CoV-2 by PCR and work and family contacts traced and followed up for 14 days, while supermarket clients were followed up through social media announcement. Descriptive analysis performed to identify size and extent of the problem.

Results:
Among 130 employees, 5 (3.8%) were confirmed as COVID-19, and 0% were asymptomatic. Symptoms included fever 100%, cough 80%, myalgia 65%, and diarrhea 30%. All patients had mild symptoms, no one was hospitalized or died out of the disease and no secondary cases reported. The hypermarket was closed for 14 days to control spread of the disease in the area.

Conclusions:
IBS and EBS both contributed to the early detection and control of a COVID-19 outbreak which erupted in one of the largest hypermarkets in Cairo. The outbreak effectively controlled through early case detection, isolation, and closure of source of infection. IBS and IBS should work in a complementary way to ensure effective and timely data collection and analysis for appropriate implementation of preventive and control measures.

Keywords:
COVID-19, EBS, IBS, Egypt, surveillance.

12 - Abstracts (Day 3, November 16th, 2021 Session 1)

COVID-19

Abstract Code: 2021-EOH-825
Presenter Name: Dr. Tareq Aldamen
Country: Jordan
Presenter Email: tareq1840@gmail.com
Authors: Dr. Tareq Aldamen, Dr. Ashraf Aqel, Dr. Mahmoud Yaqoub, Dr. Majed Asad, Mr. Mohammad M.Asad, Dr. Mohammad Alhawarat

Title: The Effectiveness of Vaccination: Hospitals Admissions and Length of Stay

Background:
COVID-19 vaccinations were first met with public hesitancy. There are some debates about the magnitude of vaccines effectiveness in reducing hospital admissions or length of stay.

Purpose:
To compare the effectiveness of different vaccines regarding hospital admissions and length of stay.

Methodology:
Information related hospital admissions, length of stay, and the need for Intensive Care Unit, and vaccinations data were obtained from the Jordanian Ministry of Health.

Results:
A total of 17,182 hospital admissions were reported since 02-02-2021. The mean age of patients was 53 years. Unvaccinated individuals reported 93.7% of admissions, comprising the highest percentage of admissions. The average hospital stays were 6.9 days and8.3 days for older and younger age groups, respectively. In addition, unvaccinated admittance to the intensive care unit were 91.2% and 93.3% for both groups. Fully vaccinated reported 3.4% admittance to the intensive care unit with varying number of length of stay, which was based on the type of vaccine as per the following: 1)Pfizer comprising 115 admissions and a length of stay of 4.9-6.1 days 2)Astrazeneca comprising 26 with 5.1-10.8 days 3)Sinopharm comprising 440 with 5.3-6.7 days and 4)Sputnik comprising 4 with 2-4 days. Regarding patients who only had the 1st dose, they reported 2.5% admittance to the intensive care unit and the following length of stay in the hospital: 1) Pfizer comprising 133 admissions with 7.05-7.25 days for the length of stay 2)Astrazeneca comprising 109 with 7.73-7.53 days 3) Sinopharm comprising 253 with 6.5-7.9 days and 4)Sputnik comprising 1 with 4 days. Moreover, the time between the vaccination and admission was noticeably longer after the second dose for each vaccine compared to one dose only, except for Astrazeneca.

Conclusions:
This study showed a lower admission and shorter stay at the hospital for those who are vaccinated, indicating the ability of vaccines to reduce the burden on the healthcare system.

Keywords:
Jordan, COVID-19, hospital admission, ICU, vaccination, length of stay

Seventh EMPHNET Regional Conference
Amman, Jordan

12 - Abstracts (Day 3, November 16th, 2021 Session 1)

COVID-19

Abstract Code: 2021-EOH-718
Presenter Name: Raheem Hussein
Country: Iraq
Presenter Email: raheem.Hussein1300a@comed.uobaghdad.edu.iq
Author: Dr. Raheem Hussein

Title: Seroprevalence of COVID-19 Among Healthcare Workers (HCWs) in Primary Healthcare Centers (PHCs) in Al-Sader City, Baghdad, Iraq, 2020

Background:
The SARS-CoV-2 infection produces a detectable immune response in most cases reported to date. A serological test can capture previous asymptomatic infections and help to assess the immune status of a subject. Iraqi HCWs are highly vulnerable to COVID-19 infection mainly because of the shortage of personal protective equipment (PPE). Al-Sader city is an overcrowded neighborhood in Baghdad which makes the HCWs serving there even more vulnerable to COVID-19.

Purpose:
To determinate the seroprevalence of COVID-19 infection among HCWs working in PHCs in Al-Sader City, Baghdad, Iraq, 2020.

Methodology:
A cross-sectional study was conducted in nine PHCs selected by a cluster random sampling technique during November and December 2020. We developed a questionnaire to gather demographic variables, history and determinants of contracting COVID-19 infection, and training and use of the PPEs. All healthcare workers had COVID-19 rapid antibody test (IgM-IgG Rapid test).

Results:
A total of 470 participants were enrolled in the study. We found that 125 (26.6%) participants had positive rapid tests, 104 (83.2%) of them had positive IgG, 5 (4%) had positive IgM, and 16 (12.8%) had positive IgG and IgM. Also, 101 (21.5%) had a history of COVID-9 infection and 75.2% were diagnosed by real-time polymerase reaction chain (RT-PCR). There was a significant association between positive rapid test and history of COVID-19 infection ($P<0.001$), and positive PCR test ($P<0.001$). The sensitivity and specificity of the rapid test as compared to PCR testing were 56.6% and 79.2%, respectively. Rapid test results and history of COVID-19 infection were significantly associated with smoking, comorbidity, training, and use of personal protective equipment, and households' infection ($P<0.05$).

Conclusions:
COVID-19 infection was common among HCWs working in the PHCs. Strong measures are needed to strengthen infection prevention and control activities including further training and enhanced use of PPEs. HCWs should be the priority group to have the COVID-19 vaccine.

Keywords:
COVID-19, Primary Healthcare Centers, Rapid Test, Iraq

12 - Abstracts (Day 3, November 16th, 2021 Session 1)

Zoonotic Diseases

Abstract Code: 2021-OTH-882
Presenter Name: Nisreen Al-Hmoud
Country: Jordan
Presenter Email: Nesreen.Alhmoud@rss.jo
Authors: Nisreen AL-Hmoud, Mumen Alrwasheh, Ghadeer Alzghoul & Amany Abiden, Kevin Olival, Kendra Phelps, Luke Hamel, and William Karesh

Title: Understanding the Risk of Bat-Borne Zoonotic Disease Emergence in Western Asia

Background:
There is a limited scientific knowledge of the distribution and ecology of bats, their pathogen diversity, and potential interfaces for transmission to humans and other species in Western Asia. This project has been initiated through a collaboration of the EcoHealth Alliance/USA, the Royal Scientific Society/Jordan, and the R. Lugar Center for Public Health Research/Georgia and supported by the Defense Threat Reduction Agency/USA.

Purpose:
The project aims to characterize the diversity of coronaviruses (CoVs) and test key hypotheses about bat-borne zoonotic virus emergence risk in Western Asia in order to reduce the threat of infectious diseases.

Methodology:
Demographic data and diagnostic samples were collected from 540 bat individuals from 6 different bat species at 8 sites in the north, middle and south of Jordan. The specimens were rectal and oral swabs, blood, and wing punch. Basic measurements such as forearm and tail lengths, sex, and reproductive status were also taken to help in identifying the bat species. All samples were tested for the presence of Coronaviruses using molecular techniques; in addition to molecular characterization of selected bat species in order to associate the presence of infectious diseases to the bat species and study their biodiversity within the region.

Results:
Using PCR and sequencing techniques, 37 bat samples were characterized with positive coronavirus strains. The majority of these strains were bat coronaviruses strains with low zoonotic potential. More analyses should be conducted to study the similarities and the relativeness of these strains to other coronaviruses strains with zoonotic potential. In addition, 94 selected bat species were characterized using DNA barcoding assay to the Cyt-b gene in order to identify the bat species and study the genetic diversity of these species in the region.

Conclusions:
This project has succeeded in promoting zoonotic disease awareness and risk reduction for bat-borne viruses.

Keywords:
Bats, Coronaviruses, RT-PCR, WAB-Net, Zoonotic Diseases

12 - Abstracts (Day 3, November 16th, 2021 Session 1)

Vaccine Preventable Diseases

Abstract Code: 2021-EOH-746
Presenter Name: Adel Alotaibi
Country: Saudi Arabia
Presenter Email: alotaibiadel22@gmail.com
Author: Dr. Sami Almudarra

Title: Adherence to Vaccine Requirements Among Hajj Pilgrims in Saudi Arabia, 2017-2019

Background:
Hajj pilgrims are required to have certain vaccines for pilgrimage; these requirements aim to reduce spread of infectious disease.

Purpose:
This study analyzed the trends in vaccine adherence for meningitis, yellow fever, and polio from January 2017- December 2019.

Methodology:
We analyzed data using descriptive statistics for Hajj pilgrims and Hajj seasonal workers arriving in Saudi Arabia over three years (2017–2019). Health Control Centers (HCCs) collected data at points-of-entry (PoE) and entered it into Saudi Arabia's Health Electronic Surveillance Network (HESN). We reviewed HESN data to collect information on total passengers arriving per country, and number of passengers vaccinated for: meningococcal meningitis, poliomyelitis, and yellow fever. We compared data to identify difference in vaccination by region. We used chi-square tests to assess differences in compliance rate among these travelers by year and country of origin.

Results:
Meningitis vaccine coverage increased by 5% from 2017–2019. Asia had the lowest overall adherence rate (83%). Yellow fever adherence decreased significantly ($p<0.01$). Polio vaccination adherence declined by 5%, but this was not significant ($p=0.08$). Adherence to polio vaccine requirements decreased over time in Asian and African countries.

Conclusions:
Less than 100% vaccine adherence among Hajj travelers creates the potential for spread of infectious disease. Proof of vaccination should be required in submitted visa applications. Countries of origin and Saudi Arabia must work together to ensure that all Hajjis are adequately vaccinated before departure.

Keywords:
Hajj, meningitis, yellow fever, polio, vaccination

12 - Abstracts (Day 3, November 16th, 2021 Session 1)

Vaccine Preventable Diseases

Abstract Code: 2021-EOH-824
Presenter Name: Fawad Khurshid
Country: Pakistan
Presenter Email: fawad_khurshid@yahoo.com
Authors: Dr. Muhammad Khalil Ahmad

Title: Epidemiological Determinants for Mortality from Neonatal Tetanus in Province Punjab, Pakistan - 2020

Background:
Neonatal Tetanus (NNT) is a vaccine preventable disease that occurs at higher incidence in resource-poor countries, presumably because of low maternal immunization rates and unhygienic cord care practices. Neonatal Tetanus (NNT) remains an important cause of infant mortality in rural areas of Punjab Province.

Purpose:
To evaluate and determine the risk factors for mortality in NNT cases and to make the recommendations for future strategies.

Methodology:
A descriptive study was conducted from July 6th to 14th 2021 at DG Health Office Lahore. Surveillance data set for the year of 2020 and clinical notes were reviewed and analyzed. Demographic information, clinical presentation progression and outcome was evaluated from all investigated cases and comparison analysis was done between those who survived and those who died.

Results:
Out of total of 176 reported cases, 145 (82.3%) cases were notified from rural areas of Punjab. Mean age was 9 days, 37% cases were females and 67% were males. The overall mortality was 77 (43.75%) while 31 (17.6%) maternal deliveries were conducted by untrained birth attendants. 119 (67.6%) women received zero TT shot in their life. Clinical notes revealed the group who survived had a significantly greater mean body weight on admission, later onset of disease, was hospitalized early and received Tetanus Immunoglobulin (TIG). The children who could not survive had significantly common clinical feature like generalized rigidity, fever, and respiratory arrest.

Conclusions:
Number of increased mortality in rural and tribal areas is suggestive of poor TT immunization coverage. Low literacy, poor socio-economic status of families, lack of awareness regarding antenatal care and poor hygienic deliveries conducted by untrained persons remained main risk factors. Improvement in TT coverage, deployment of trained community midwives and awareness sessions regarding TT vaccination in hard to reach areas is recommended.

Keywords:
Epidemiological Determinants, Neonatal Tetanus, Punjab, TT Immunization

12 - Abstracts (Day 3, November 16th, 2021 Session 1)

Vaccine Preventable Diseases

Abstract Code: 2021-EOH-740
Presenter Name: Hanan Al-Marbouai
Country: Oman
Presenter Email: hananh_19@hotmail.com
Authors: Dr. Ahmed Al-Buloshi, Dr. Mohammed Muqeet Ullah, Dr. Randa Nooh

Title: Imported Measles Outbreak at Al-Buraimi Governorate, Oman, April 2020

Background:
On April 16, 2020, the communicable disease department of Al-Buraimi Governorate, Oman, was notified of three cases of measles. On laboratory confirmation on 19 April, further field investigation was conducted. Oman has experienced few cases of measles since 1995, however, Al Buraimi has experienced imported measles cases in recent years..

Purpose:
We conducted this study to investigate the epidemiology of imported measles cases at Al Buraimi, Oman, in April 2020.

Methodology:
This case series retrospectively investigated reported measles cases. Epidemiological investigation began by meeting the families of the affected children. Obtained data included clinical symptoms, exposure information, travel history, immunization, and history of contact with others.

Results:
From the number of positive cases, we found that 75% were girls while 25% were boys. Also, six patients were Afghani nationals, while two were Pakistani. A detailed investigation that included virus isolation and genotyping identified all the cases were Measles B3 genotype and traced the virus back to Pakistan as the country of origin. However, despite Pakistan being the virus's origin, most cases were reported from Afghani's (75%) nationals due to low vaccination coverage. Also, we established that most of the children affected were from 10-19 years (75%). All children who did not have vaccination records or were unvaccinated, whether they had fallen ill or not, were given MMR vaccine. This action was done to avoid future outbreaks and increase measles vaccination coverage.

Conclusions:
This study demonstrated that the greatest challenge of measles elimination in Oman is non-Omanis expatriates and unvaccinated children. It should therefore be a priority to vaccinate all expatriates' children. When everyone is vaccinated in Oman, nationals or foreigners, only then can the goal of a measles-free country be realized.

Keywords:
imported measles, Al Buraimi, Oman, Children, Afganistan, Pakistan, vaccinated, unvaccinated

12 - Abstracts (Day 3, November 16th, 2021 Session 1)

Vaccine Preventable Diseases

Abstract Code: 2021-HIV/STI-869
Presenter Name: Homeira Nishat
Country: Afghanistan
Presenter Email: Homeiragulzar@gmail.com
Author: Dr. Khawja Mir Islam Saeed

Title: Descriptive Epidemiology of Acute Flaccid Paralysis Cases in Afghanistan, 2015 - 2018

Background:
Polio is on the verge of eradication, while Afghanistan and Pakistan are the only endemic countries remaining where polio is still prevalent. Surveillance for Acute Flaccid Paralysis (AFP) is one of the four cornerstone strategies of the Polio Eradication Initiative.

Purpose:
This study aims to describe the epidemiology of AFP cases in terms of time, place and person.

Methodology:
It is a descriptive study whereby we analyzed the secondary data reported by AFP surveillance in Afghanistan. We accessed and used line-lists from 2015-2018 to describe the epidemiological status of AFP cases in the country. With the use of Epi Info 7 and Microsoft Excel, we calculated descriptive measures including frequencies, mean, median, standard deviation, generated proportions, tables, and graphs.

Results:
Overall, 11,513 cases were reported in the last four years (2015-2018) by AFP surveillance, ministry of public health. Majority of the cases (29%) were reported in 2018 while 2088 (18%) cases were reported in 2015. The trend of OPV vaccination has increased from 2015 to 2018 (57%, 64%, 63% and 68%, respectively). Most of the cases were reported from southern and western regions, 57% of which was comprised of male cases. The highest (38%) proportions of cases were in individuals less than 30 months of age. Guillain–Barre syndrome (GBS) was 38% of all categories. The samples were collected using appropriate procedures. However, the numbers of confirmed cases increased from 13 in 2016 to 14 in 2017, 20 in 2018 and 22 in 2019.

Conclusions:
The AFP surveillance system is well-established in the country. Nevertheless, with the increase in the trend of OPV coverage there is also increase in number of confirmed polio cases. Hence, the system should be sustained and strategies should be strengthened to focus on southern region as being the main engine of polio in the country.

Keywords:
Epidemiology, Acute Flaccid Paralysis, Poliomyelitis, Afghanistan

12 - Abstracts (Day 3, November 16th, 2021 Session 1)

Vaccine Preventable Diseases

Abstract Code: 2021-EOH-816
Presenter Name: Dr. Mohammad AlFauri
Country: Jordan
Presenter Email: malfauri@gmail.com
Authors: Dr. Mohammad AlFauri, Dr. Adnan Ishaq, Dr. Asharf Aqel, Dr. Majed Asad

Title: Chickenpox in Jordan Between 2013 – 2020

Background:
Chickenpox is the primary infection of Varicella-Zoster Virus (VZV) that is highly contagious causing itchy, blister-like rash that could reach up to 500 itchy blisters. This disease is highly prevalent among school children and the Jordanian health surveillance system reported a rise in the number of cases among school children in 2019.

Purpose:
To examine the rates of chickenpox among different seasons of the year during 2013 – 2020. In addition, reasons of outbreaks or a decrease in the number of cases were explored.

Methodology:
This is a descriptive epidemiological study that included school children younger than 15 years old. Chickenpox cases in Jordan are reported to the Ministry of Health from all kinds of health care sectors on a weekly basis.

Results:
The total number of cases reported was 40,988 between 2013 and 2020. There was an obvious rise by 19.24% in 2014 accompanying the huge influx of Syrian refugees during that year. On the other hand, the lowest rate was reported in 2020 during the pandemic of Covid-19 that included a quarantine period with schools' closure resulting in a 2.44% reduction. Most cases were among the 5-9 age group (43.34%). Although the total number of chickenpox varied from 2013 to 2020, all had shown the same seasonal distribution, being highest in spring, especially in May, reaching 16.12%. Geographic distribution showed that the highest number of cases were reported in Amman (18.15%) and Al-Zarqa (12.81%). In Al-Mafrak city, where more Syrian refugees reside, high rates of infection among children younger than 4 years old was noticed. Overall, boys rates were higher than girls (54.5% vs. 45.5%).

Conclusions:
The high rates of chickenpox among young children could be explained by their lower immunity and higher interaction with each other compared to older ages. Although the health surveillance system for chickenpox in Jordan in effective, outbreaks caused by conflicts, such as the Syrian war, could suddenly increase the rates. This should be considered and properly handled whenever a rise in cases is expected.

Keywords:
Chickenpox, Seasons, Jordan, Syrian refugees, Children.

12 - Abstracts (Day 3, November 16th, 2021 Session 1)

Vaccine Preventable Diseases

Abstract Code: 2021-EOH-708
Presenter Name: Nora Ali
Country: Iraq
Presenter Email: nora.mohammed1300a@comed.uobaghdad.edu.iq
Authors: Dr. Nora Ali

Title: Impact of COVID-19 Pandemic on Immunization Services Performance, Iraq, 2020

Background:
The COVID-19 pandemic impedes the health systems' capacity to deliver curative and preventive services. Disruption of immunization services can increase the probability of Vaccine-Preventable Diseases outbreaks.

Purpose:
To evaluate the impact of the pandemic on the coverage rate of the essential vaccines (BCG, DTP1, DTP3, and Measles) administered to <1-year old children in Iraq and identify the most affected governorates.

Methodology:
A descriptive desk review of the vaccination coverage administrative data of the essential vaccines (BCG, DTP1, DTP3, and Measles) included in the routine immunization schedule for the first six months of 2020 and compared it to the same period of 2019. The coverage rate difference was calculated as (2019 coverage rate - 2020 coverage rate) /2019 coverage rate *100%.

Results:
The coverage rate of the BCG, DTP1, DTP3, and Measles was reduced in the 1st half of 2020 by 4.28%, 11.08%, 20.48%, and 13.67%, respectively, compared to the 1st half of 2019. Mid and southern governorates and Sulaymaniyah (that reported the highest number of COVID-19 cases) showed the highest impact on vaccine coverage. For the BCG vaccine, apart from Duhok, Salahuddin, and Anbar, all other governorates witnessed a decline in vaccine coverage in 2020 compared to 2019. The highest decline was seen in Baghdad Resafa and Ninewa (-9%). For the DTP1 and DTP3, only Duhok, and Anbar demonstrated an increase in the coverage rate. The highest decline in DTP1 was in Diwaniya (-31%) and Najaf (-28%). Also, for DTP3, Diwaniya and Najaf reported the highest decline (-38% and -37%, respectively). For the measles vaccine, only Duhok showed an increase in vaccine coverage (7%) in 2020 compared to 2019. Baghdad Resafa and Najaf reported the highest decline (-28%).

Conclusions:
The pandemic had negatively affected the vaccination coverage of the main vaccines. An intensive response to close the immunization gap is recommended particularly for poorly performing governorates.

Keywords:
Vaccination, COVID-19, Impact, Iraq

Seventh EMPHNET Regional Conference
Amman, Jordan

12 - Abstracts (Day 3, November 16th, 2021 Session 1)

Vaccine Preventable Diseases

Abstract Code: 2021-OTH-883
Presenter Name: Amine Amriche
Country: Dubai, UAE
Presenter Email: amine.amiche@sanofi.com
Authors: Soudani S, Mafi A, Mayahi Z , Al Balushi S , Dbaibo G , Al Awaidy S, Amiche A

Title: A Systematic Review of Influenza Epidemiology and Surveillance Activities in the Eastern Mediterranean and North African Region

Background:
Seasonal influenza causes a significant disease burden in the Eastern Mediterranean and North African (EMNA) region.

Purpose:
We aimed to describe the epidemiology of influenza in the EMNA region and assess the landscape for influenza epidemiological research.

Methodology:
We conducted a systematic literature review leveraging online databases from 1 January 1998 to 31 January 2020 covering EMNA countries. Grey literature was screened to identify missed articles. Titles, abstracts, and full-texts screening and selection were sequentially performed. Relevant data from the included articles were summarized using a standardized data extraction form.

Results:
A total of 10,585 studies were initially identified and 112 studies were retained. The resultant studies were carried out in 18 EMNA countries. Turkey, Iran and Saudi Arabia had the highest number of articles with 25, 24 and 15 respectively. Most studies were conducted among hospitalized patients (89 studies, 79%). The median study duration was 24 months. Study populations were predominantly identified as the general population (69 studies, 62%). Various case definitions for influenza have been used, yet influenza-like illness (ILI) was the most common one (48 studies, 43%). Most studies (86, 77%) employed polymerase chain reaction to detect influenza virus. Twenty-seven articles reported data on A strain circulation only, 1 on B strain only, and 83 on A and B. Overall, 275,717 laboratory-confirmed influenza cases were identified in the studies. In studies reporting both influenza A and B circulation, the median proportion of dominant virus were 67.6% and 27.0% for Influenza A and B respectively. Subtyping of influenza A (H1N1, H3N2) and/or B (Yamagata, Victoria) was conducted in 58 studies. Other viruses were assessed in 48 studies.

Conclusions:
Gaps in influenza epidemiology and surveillance are still significant in the EMNA region. Strengthening regional research might be needed to improve seasonal influenza prevention and pandemic response.

Keywords:
Influenza, Epidemiology, Eastern Mediterranean and North African region, Surveillance

Funding Statement:
This study was funded by Sanofi Pasteur.

13 - Abstracts (Day 3, November 16th, 2021 Session 2)

Surveillance

Abstract Code: 2021-HIV/STI-695
Presenter Name: Maeen Abduljalil
Country: Yemen
Presenter Email: maeenabdugalil28@gmail.com
Authors: Dr. Methaq Al-Sada, Dr. Moamer Badi, Dr. Yaser Ghalab

Title: Evaluation of Malaria Surveillance System in Hodeidah City, Yemen, 2021

Background:
Despite continuing control and elimination efforts, malaria continues to represent a major public health problem. Evaluation of Malaria Surveillance System (MSS) is critical to get credible data which is used for providing information. Hodeidah city is worthy to conduct an evaluation for the MSS because of the greatest malaria burden.

Purpose:
To determine the usefulness of MSS and assess the performance in terms of qualitative and quantitative attributes.

Methodology:
Updated CDC guideline was used to evaluate MSS in Hodeidah city. After desk reviews and in-depth interviews conducted; self-administered questionnaires with five point Likert scale and (Yes /No) questions were used to collect data from stakeholders at four levels. The indicator's score percent was interpreted according to the following criteria: Excellent: ≥ 90%, good: 80-<90%, average: 60-< 80%, poor: 40-<60%, and very poor: < 40%. The EPI info version 7.2 was used to enter and analyze the data.

Results:
Thirty-one stakeholders participated; out of the total 55% of responders were males. The system was found to be useful (88%) to portray the trend of malaria, guide policy and intervention, with excellent timeliness and completeness (100%). The overall simplicity, representativeness, acceptability and stability scores were 78%, 66%, 62%, and 61%, respectively with average rank. However, it scored only 40% for flexibility and 5.5% for sensitivity. The overall performance scores of MSS were average 62%, good 82%, and average 73% in central, governorate, district and health facilities, respectively.

Conclusions:
Although the MSS was found to be useful, stable, data quality, and timeliness were excellent; flexibility and sensitivity were still poor. To ensure the MSS sustainability, there is a need for gradual replacement of donors' fund with governmental fund. Furthermore, enhancing laboratory diagnosis and proper training of the health workers should be adopted for improving flexibility and sensitivity.

Keywords:
Malaria, Evaluation, Surveillance, Yemen

Seventh EMPHNET Regional Conference
Amman, Jordan

13 - Abstracts (Day 3, November 16th, 2021 Session 2)

Surveillance

Abstract Code: 2021-HIV/STI-640
Presenter Name: Magdi Aldaari
Country: Yemen
Presenter Email: Magdid14@gmail.com
Authors: Dr. Labiba Annam, Dr. Sami Alhaidari

Title: Evaluation of Leishmania Surveillance System, Yemen, 2021

Background:
Control of preventive chemotherapy-targeted neglected tropical diseases (PC-NTDs) depends on strengthened health systems. Efficient health information systems provide stimulus to reaching the sustainable development goal aimed at ending PC-NTD epidemics. However, there is a limited assessment of surveillance system functions linked to PC-NTDs and hinged on optimal performance of surveillance system attributes.

Purpose:
The study aimed to assess the usefulness and performance of the system, estimate the strength, and weakness points of the National Leishmania Control Program.

Methodology:
We followed the updated six steps of the Center for Diseases Control and Prevention guidelines (CDC) for evaluating public health surveillance systems. Data were collected using in-depth interviews with relevant stakeholders at the central level and semi-structural questionnaire format at the peripheral level. We used questions (Yes, No) to assess the usefulness, and the 5-Likert scale to measure the attributes. The final score interpreted as poor (<60), average (60-80), and good (>80).

Results:
NLCP seemed to be useful (86%); some of its objectives were met. The system has average performance in flexibility (78%), simplicity (64%), acceptability (80%), and data quality (65%). Stability and timeliness had a poor performance (33%, and8%, respectively). The overall performance of NLCP was poor (55%). Continuation of the system was the strongest point while the lack of governmental and agency fund was the weak point.

Conclusions:
The NLCP was found to be useful regarding the attributes, although simplicity, flexibility, acceptability, and data quality were average, and stability and timeliness were poor. Governmental financial support to the program is highly recommended. In addition, create a database for staff at the peripheral level, expand the number of health facilities that serve as Leishmania units are prerequisites.

Keywords:
Evaluation, Surveillance System, Leishmania, Yemen.

13 - Abstracts (Day 3, November 16th, 2021 Session 2)

Surveillance

Abstract Code: 2021-HIV/STI-697
Presenter Name: Monia Benblal
Country: Morocco
Presenter Email: benblalmonia@gmail.com
Author: Dr. Monia Benblal

Title: Meningitis Database Analysis Report at the Prefecture of Salé, Morocco, 2015 - 2019

Background:
Globally, more than 1.2 million cases of bacterial meningitis occur each year, with a higher incidence in developing countries. In Morocco, meningitis is a public health problem. The meningitis epidemiological surveillance system is continuous, comprehensive and passive. It is based on mandatory reporting of all forms of meningitis. At the same time, the alert system ensures the monitoring and early detection of possible epidemics at local and regional levels.

Purpose:
Our study aimed to describe the epidemiological profile of meningitis patients in the province of Salé in Morocco, in order to suggest improvement measures.

Methodology:
We carried out a descriptive study, with a purely quantitative approach. EPI-INFO 7 software and Microsoft Excel 10 were used for the analysis and production of the graphs.

Results:
The total number of registered meningitis cases was 132 cases. The median age was 6.5 years, and the modal age group was 5-9 years with 24.24% of cases. The sex ratio M / F was 1.53, and 80.3% belonged to urban areas. The main source of the statement was the children's hospital in Rabat with 78% of the cases reported. Between 2015 and 2019, MA was only confirmed for (5.3%), while MBP and MMP were attributed respectively for 16.66% and 21.96% of notified cases. The notion of purpura concerns 26.51% of notified cases of meningitis. The five-year average case fatality rate was 10.6%. 30.3 % of the results of the CSF laboratory analysis did not exist, and 37.87% were not complete, these percentages obtained from the analysis of the epidemiological situation were less relevant at the prefectural level.

Conclusions:
We therefore recommend creating a bacteriology laboratory for meningitis at the prefectural level, to strengthen the diagnosis, to work and to obtain the communication of the laboratory results in order to improve the performance of the surveillance.

Keywords:
Epidemiology, Surveillance System, Meningitis, Salé, Morocco

13 - Abstracts (Day 3, November 16th, 2021 Session 2)

Surveillance

Abstract Code: 2021-HIV/STI-769
Presenter Name: Neven Girgis
Country: Egypt
Presenter Email: nevengirgis72@gmail.com
Authors: Dr. Alaa Eid, Dr. Hala Bahaa Eldin, Dr. Hanaa Abu Elsood, Dr. Sahar Samy, Dr. Salma Afifi, Dr. Wessam Elnahry

Title: Evaluation of the National Electronic Disease Surveillance System (NEDSS) Amid COVID-19 Pandemic, Elsahel District, Cairo Governorate - Egypt, 2020

Background:
Egypt National Disease Surveillance (NEDSS) is a routine system established in 2002. The system electronically reports 41 infectious diseases, including COVID-19. Reporting sites include all Egyptian governorates, districts, governmental infectious diseases hospitals, and primary health units. Surveillance is essential during pandemics to detect cases early, describe the epidemiology of health problems, guide priority-setting, and plan and evaluate public health policy and strategies.

Purpose:
Evaluation of surveillance during the pandemic is necessary to assess its effectiveness in achieving these objectives, find and fill the gaps.

Methodology:
The evaluation was performed using CDC guidelines. A structured questionnaire was used to evaluate the qualitative attributes including simplicity, flexibility, and acceptability through interviewing surveillance teams at the central level, health directorate, and Sahel district. While quantitative attributes including completeness, timeliness, and positive predictive value were performed using COVID-19 surveillance data of Sahel district March-December 2020. Data were assessed for completeness and accuracy. The usefulness of surveillance was assessed in terms of achieving its objectives and utilization of data.

Results:
Out of 33 respondents, 90% think that the system is simple, 77% acceptable, work overload reduced the acceptability rate. The system is funded by MoHP and operational 53% of the time due to connectivity problems. The system was flexible when adapting to include COVID-19 in a short time with minimal cost. It is quite representative as it covers 60% of the population. Completeness of the system was 82%, positive value predictive 58%, and data validity 86%. The median duration between patient admissions and reporting was 2.7 days.

Conclusions:
Evaluation of Egypt's COVID-19 surveillance system indicated that the system partly achieved its objectives in the area of simplicity, flexibility with adequate data quality. There is a need to improve acceptability and timeliness through increasing workforce and enhancing stability through effective connectivity. Expansion of the system to cover all of Egypt's population is recommended to improve representativeness.

Keywords:
NEDSS, Evaluation, Egypt, surveillance, COVID-19

13 - Abstracts (Day 3, November 16th, 2021 Session 2)

Surveillance

Abstract Code: 2021-HIV/STI-756
Presenter Name: Samreen Qureshi
Country: Pakistan
Presenter Email: qsamreen@yahoo.com
Author: Dr. Saeed Ahmed Bhurt

Title: Surveillance System Evaluation1 for COVID-19 Vaccine Associated Adverse Events Following Immunization (AEFI), Sindh Pakistan 2021

Background:
In February 2021, a mass vaccination campaign commenced in Sindh province in response to the COVID-19 epidemic. An adverse-events following immunization surveillance system (AEFI-SS) was established to monitor the adverse event following vaccination.

Purpose:
We evaluated the AEFI-Surveillance System to identify the strengths and weaknesses and suggest recommendations.

Methodology:
In May-June 2021, a Descriptive evaluation study was done in Sindh-Pakistan. CDC's updated guidelines for evaluation of surveillance system-2001 were followed to measure the qualitative, quantitative, and utility attributes of the AEFI-SS. Key stakeholders were identified based on their involvement in AEFI-SS and were interviewed. Case investigation proformas for AEFI were randomly reviewed for data quality, timeliness, and completeness. Sensitivity was calculated. Each attribute was rated as good, fair, and poor, based on a scoring legend.

Results:
The Surveillance system was helpful in identifying n=7147 cases of AEFI effectively. Timeliness of all AEFI cases was good and was found 100% as all cases were reported within 24 hours. WHO-approved case definition was being used to identify AEFI cases and had a simple flow of information. AEFI-SS was good in data quality and completeness (100%), trained medical officers filled data collection tools. Sensitivity was 100%, positive predictive value (PVP) was not calculated due to the absence of a laboratory component. There is a good representativeness as >80% of the population is covered by 1004 vaccination centers. System was found stable as resources of the health department government of Sindh were being used. AEFI-SS was paper-based and deficient in the feedback mechanism.

Conclusions:
The Sindh Province has an appropriate surveillance mechanism for AEFI detection and management for the ongoing COVID-19 vaccination surveillance system. The representativeness can be increased by the involvement of the private health sector. The establishment of feedback mechanism and digital data transformation and integration of the AEFI system with EPI is recommended.

Keywords:
COVID-19, Surveillance System Evaluation, AEFI, Province Sindh

Seventh EMPHNET Regional Conference
Amman, Jordan

13 - Abstracts (Day 3, November 16th, 2021 Session 2)

Surveillance

Abstract Code: 2021-HIV/STI-714
Presenter Name: Manal Mahsoon
Country: Yemen
Presenter Email: manalmahsoon@gmail.com
Authors: Dr. Mohammed Al Amad, Dr. Riham Al-dubaiee

Title: Surveillance Evaluation for Severe Acute Respiratory Infection, Sana'a City, Yemen, 2021

Background:
Due to the war and limited access to health facilities, surveillance of severe acute respiratory infection (SARI) has been expanded to include all hospitals since 2017.

Purpose:
To assess the usefulness of SARI surveillance in Sana'a city and to assess its performance in terms of attributes.

Methodology:
The CDC's updated guideline was used for evaluating surveillance systems. Four qualitative attributes, including; stability, simplicity, flexibility, acceptability, and data quality as quantitative attributes, were assessed. In-depth interviews with stakeholders at central level and self-administered questionnaires with five Likert scale and register review at peripheral level were used for collecting data. Scoring for indicators was used to calculate the total gained scores for each attributer and percentage for ranking as poor < 60 %, average 60 - <80%, good 80% - < 90%, and excellent ≥ 90%.

Results:
The SARI surveillance was useful and accounted for 94% gained scores. The overall performance for the five attributes was average (64%). It was good (82%) at the central level, where flexibility was excellent (93%), and stability was average (72%). The performance at the peripheral level was poor (51%), while simplicity (61%), and acceptability (74%) were average, and the data quality was poor (20%).

Conclusions:
Expanding SARI surveillance with lack of staff training, central communication, and supervision might be the main reason for weak performance at the peripheral level. Supporting SARI program activities, selection of SARI reporting sites, and a surveillance team based on WHO criteria are highly recommended.

Keywords:
Evaluation, National Influenza Control Program, CDC guidelines, Yemen

13 - Abstracts (Day 3, November 16th, 2021 Session 2)

Zoonotic Diseases

Abstract Code: 2021-EOH-731
Presenter Name: Aman Ullah
Country: Pakistan
Presenter Email: amanvet@gmail.com
Authors: Dr. Aitzaz Ahsan, Dr. Siddra Akhtar

Title: Serological Evidence of SARS-CoV-2 Infection in Companion Animals in Pakistan

Background:
A novel coronavirus, SARS-CoV-2, is associated with the COVID-19 pandemic, a zoonosis with its origins from live animal markets in Wuhan, China. It can transmit from humans to other animals and within animal species both naturally and experimentally. Being a novel virus, studies are needed to understand how it affects animals, possible spread to humans, and other potential roles in the epidemiology of COVID-19 following a one health approach.

Purpose:
Therefore, we conducted a cross-sectional serosurvey among companion animals (dogs and cats) in twin cities of Islamabad and Rawalpindi, Pakistan to determine the seroprevalence of COVID-19 among these animals.

Methodology:
The study was conducted from November 2020 to April 2021, coinciding with the end of the 2nd wave and peak of the 3rd wave of COVID-19 in Pakistan. We collected serum samples from 83 animals (68 dogs and 15 cats) from 10 small animal clinics in twin cities. The data were collected on the species, age, sex, clinical history, travel history, and confirmation of COVID-19 among the owners or their families. the cases were presented with various clinical histories. The samples were tested by ID Screen® SARS-CoV-2 double antigen multi-species ELISA developed by ID. Vet, France.

Results:
A combined seroprevalence of COVID-19 among these companion animals of 7.23% (6/83, 95%CI; 2.7%-15.07%) was found. Further, the seroprevalence of COVID-19 among dogs was 7.35% (5/68, 95%CI; 2.43%-16.33%) and 6.67% (1/15, 95%CI; 0.17%-31.95%). Both species were found similarly susceptible to COVID-19 (OR=1.11, 95%CI; 0.12-10.27, p=0.92). Animals in families with a history of COVID-19 among owners were more likely to be seropositive for SARS-CoV-2 (OR= 11.8, 95%CI; 1.93-71.89, p=0.002).

Conclusions:
The results suggest a possible transmission of SARS-CoV-2 from pet owners to their companion animals. However, further studies may be needed to evaluate this hypothesis and the role of pets as potential reservoirs for SARS-CoV-2 infection for humans and other animals.

Keywords:
COVID-19, pets, Pakistan

Seventh EMPHNET Regional Conference
Amman, Jordan

13 - Abstracts (Day 3, November 16th, 2021 Session 2)

Zoonotic Diseases

Abstract Code: 2021-OTH-691
Presenter Name: Javaria Alam
Country: Pakistan
Presenter Email: jaweria.alam@gmail.com
Author: Ms. Syed Nadeem Ur Rehman

Title: Assessment of Farmers' Knowledge, Attitude and Practices on Milk-Borne Zoonosis in District Muzaffarabad, Azad Jammu & Kashmir

Background:
Milk-borne zoonotic disease can be acquired by the consumption of non-pasteurized and infected dairy products. Zoonotic infections present a serious public health concern that is responsible for approximately 2.7 billion deaths annually worldwide. However, little is known about the attitudes and knowledge of the farmers regarding milk-borne zoonosis.

Purpose:
This study was performed to assess the knowledge, attitude, and practices of the farmers regarding milk-borne zoonosis.

Methodology:
The cross-sectional KAP study was conducted in districts Muzaffarabad, Azad Jammu, and Kashmir between September 1st to October 30th ,2019. A pretested semi-structured questionnaire was used to collect the information from respondents regarding different aspects of milk-borne zoonosis. All the small dairy farms (n=56) with more than five animals in district Muzaffarabad were included in this study. Data were collected from respondents (n=100) with inclusion criteria of having a dairy experience of more than six months.

Results:
The findings show that almost 86% of the farmers could not name any milk-borne zoonotic disease. About 45.5% of the farmers were unaware of the fact that milk can be a potential source of disease transmission. None of the respondents had any idea about the pasteurization method, and 50% of them had no habit of checking milk quality. However, 81% of the respondents prefer to use boiled milk. Almost 28% of the farmers with high-level education were able to name at least one milk-borne zoonotic disease. The majority of the respondents (99%) did not receive any formal training about zoonotic diseases.

Conclusions:
According to the study, the overall knowledge of farmers regarding milk-borne zoonosis was not adequate. Awareness about the importance of zoonotic disease and their source of transmission should be created, and to deal with zoonotic infections, a One Health approach should be adopted.

Keywords:
Attitude, Knowledge, Milk borne zoonosis, Muzaffarabad, Practices, AJK

13 - Abstracts (Day 3, November 16th, 2021 Session 2)

Zoonotic Diseases

Abstract Code: 2021-HIV/STI-839
Presenter Name: Kazi Tahmina Karim
Country: Bangladesh
Presenter Email: kazitahmina22@gmail.com
Authors: Dr. Eric Brum, Dr. Mallick Masum Billah, Dr. Meerjady Sabrina Flora, Dr. T.A.B.M Mozaffar Osmani

Title: Antibiotic Usage Pattern in Sub-District Chicken Farms of Bangladesh

Background:
Irrational use of antibiotics in chicken can lead to emergence of antimicrobial resistant pathogen in environment and human. In Bangladesh, veterinarian and other persons prescribe antibiotics in chicken where other person includes dealer, pharmacist, sales representative. To know antibiotic usage pattern in chicken farms, livestock department of Bangladesh conduct surveillance through the collection of information on antibiotic use from poultry farms.

Purpose:
To find out the antibiotic usage pattern in chicken farms by the different prescribers.

Methodology:
Descriptive analysis was performed on type of antibiotics from the Department of Livestock surveillance dataset where antibiotics were categorized as per World Health Organization (WHO) into access, watch, and reserve groups. Access antibiotics were available and had low resistance potential; watch group had highest priority agent and higher resistance potentials, and reserve group was highly specific to the patient and last resort of treatment. The antibiotic usage pattern by different prescribers was compared.

Results:
Of 472 chicken farms from 78 Upazilas assessed where 91% farms used antibiotics, and 1,243 antibiotic usage events were reported. Most farms commonly use three antibiotics. Poultry dealers were found to be the most common source of antibiotics (62%) followed by the pharmacist (24%), sales representatives (11%), veterinarians (4%). Dealers, pharmacists, sales representatives, and veterinarians were often found distributing multiple antibiotics to the same farm (93%, 87%, 84%, and 84%, respectively). The distribution of WHO Watch and Reserve antibiotics by dealers, sales representatives, pharmacists, and veterinarians were similar, with the majority of antibiotics being reported from the WHO Access category (56%) followed by Watch (33%) and Reserve (12%).

Conclusions:
We found farmers are using multiple types of antibiotics in the farm commonly suggested by the dealers. Therefore, we recommended training the all-possible prescriber in prescribing watch and reserve drug and the study need to explore the reasons of farmer's frequent involvement with dealer.

Keywords:
AMR, Antibiotic usage pattern, Access, Watch and Reserve antibiotic

Seventh EMPHNET Regional Conference
Amman, Jordan

13 - Abstracts (Day 3, November 16th, 2021 Session 2)

Zoonotic Diseases

Abstract Code: 2021-EOH-833
Presenter Name: Dr. Maisarah Alfreihat
Country: Jordan
Presenter Email: Maisarah.m.alfreihat@gmail.com
Authors: Dr. Basil Abuhdeeb, Dr. Maisarah Alfreihat, Dr. Ashraf Aqel, Dr. Yousef Al-Gaud

Title: The Incidence of Rickettsia Conorii in Jordan During 2015-2017: A Descriptive Study

Background:
Rickettsia conorii is a gram-negative bacterium causing Mediterranean Spotted Fever (MSF). MSF is endemic in Mediterranean countries and different areas in Asia and Africa, with a mortality rate of up to 32%.

Purpose:
To determine the incidence and epidemiology of Rickettsia conorii in Jordan during 2015-2017.

Methodology:
This was a descriptive study that included all Jordanian patients who were suspected of having rickettsia from 2015 to 2017. Data of 260 suspected cases were obtained from the Jordanian Ministry of health. The data included socio-demographic factors (age, sex, residency) and laboratory results (confirmed cases were IgM positive).

Results:
Based on the laboratory results, 145 (55.8%) cases were negative, 78 (30.0%) were positive, and 37 (14.2%) were equivocal. The confirmed cases were 78 (44 males and 34 females) with a mean age of 5.9 (+4.0). The male to female ratio was 1.29. Two-thirds (66%) of cases were younger than 15 years. The majority of cases were from the middle region (70.5%), followed by the south region (28.2%), and the rest were from the North. In addition, most cases were diagnosed during July, followed by August, September, and October. The incidence rate of Rickettsia conorii was 0.2/100000 in both 2015 and 2016 and it was 0.3/100000 in 2017. The highest incidence rate of Rickettsia conorii was among people below 5 years (0.71/100,000 in 2015, 0.27/100,000 in 2016, 0.87 /100,000 in 2017).

Conclusions:
Two-thirds of Rickettsia conorii infections were found among individuals younger than 15 years old, with a peak occurrence of the disease in summer, when the dog tick is very aggressive.

Keywords:
Jordan, Rickettsia conorii, young, summer, infection

13 - Abstracts (Day 3, November 16th, 2021 Session 2)

Zoonotic Diseases

Abstract Code: 2021-EOH-638
Presenter Name: Randa Bazzi
Country: Jordan
Presenter Email: randaalali12@outlook.com
Authors: Prof. Akram Alaboudi, Prof. Racs Gabor

Title: Evaluating the Veterinarian Role in One Health Approach at Antimicrobial Resistance Perspectives, Jordan

Background:
Antimicrobials, including antibiotics, antivirals, antifungals, and antiparasitic drugs, are medicines used to prevent and treat infections in humans, animals, and plants. Microbes will become antimicrobial-resistant because of ineffective or prolonged antimicrobial treatment (AMR).

Purpose:
This study aims to evaluate the role of the Jordanian veterinarian's knowledge, attitudes, and practices in tackling anti-microbial resistance(AMR), as well as it included the registered veterinary pharmaceutical products between 2017-2020.

Methodology:
The data of the descriptive study was collected by a standardized questionnaire that focus on the knowledge, attitudes, and practices of the Jordanian veterinarians.

Results:
The results were analyzed descriptively and showed that the average knowledge of the participant who had agreed with the statement that refers to the AMR definition was 84%. The majority (95.65%) considered AMR as a challenge for the veterinary sector in Jordan and should be prioritized beside other zoonotic diseases. A 68.70% of the participants believe that the misuse and overuse of antimicrobial by quacks, fraudulent and unauthorized practitioners are the main contributor to the AMR challenge, and the most common practices among them were recommending the customers (farmers, owner, etc..) to practice good animal husbandries (80.00%). The study also indicated that there is a significant difference (p= 0.015) between 98 veterinarians who had participated in AMR continuous education and their vocational sector (private, public and academic sector) .

Conclusions:
This study revealed the importance of implementing antimicrobial resistance continual educational program in order to improve the veterinarian knowledge in all aspects of AMR; to enhance their advisory skills. They are also obligating legislations that ensure the veterinarian to prescribe the correct antimicrobials and improve the surveillance system to monitor the antimicrobial consumption in the veterinary field.

Keywords:
antimicrobial resistance, veterinarians, knowledge, practices, attitudes , Jordan

13 - Abstracts (Day 3, November 16th, 2021 Session 2)

Zoonotic Diseases

Abstract Code: 2021-EOH-853
Presenter Name: Subrota Malakar
Country: Bangladesh
Presenter Email: malakar07@gmail.com
Authors: Dr. Ahmed Nawsher Alam, Dr. Mahbubur Rahman, Dr. Mallick Masum Billah, Dr, Md.Omar Qayum, Dr. Meerjady Sabrina Flora, Dr. Mohammad Ferdous Rahman Sarker

Title: Anthrax Vaccination and Associated Factors with Vaccination Coverage in Two Anthrax-Prone Districts During 2019 in Bangladesh

Background:
Anthrax, caused by Bacillus anthracis, is endemic in some districts of Bangladesh and is considered a significant public health threat due to repeated outbreaks in humans and animals in recent years. Department of Livestock Services (DLS) has been implementing to enhance real-time surveillance and response since 2019 in anthrax-prone districts to reduce the incidence.

Purpose:
The objective of the study was to determine the rate of anthrax vaccination and find out the factors associated with vaccination coverage in anthrax-prone districts (Sirajganj and Meherpur).

Methodology:
We analyzed the baseline survey data collected by the Preventing Anthrax and Rabies in Bangladesh by Enhancing Surveillance and Response (PARB) project from July 14 to 21, 2019. We calculated the anthrax vaccination coverage by the proportion of participants who vaccinated their cattle and expressed by percentage. We calculated the odds ratio (OR) to find out the factors associated with vaccination. We used a multivariable logistic regression (MLR) model and calculated Adjusted Odds Ratio (AOR), 95% confidence interval (CI) for statistically significant factors found in bivariate analysis.

Results:
Out of total of 881 participants, 59% (395/674) vaccinated their cattle against anthrax. Of them,48% had no formal education, and 74% lived in agriculture. In bivariate analysis, we found participants did not have basic knowledge on animal anthrax (OR:17.9, 95% CI:11-29) or human anthrax (OR:5.4, 95% CI:3.54-8.15), commonly slaughter their sick animals (OR:11.9, 95% CI:7.35-19.37) and throw animal carcasses in rivers (OR:4.4, 95% CI:0.47-1.51) are associated with low vaccination. However, in MLR, the absence of basic knowledge on animal anthrax (AOR:5.8; 95% CI:2.3-14.2), farmer slaughter their sick animals (AOR:3.7; 95% CI:1.3-10.0) and throw the carcass in the river (AOR:2.6; 95% CI:1.2-5.6) were significantly associated with low vaccination.

Conclusions:
The findings reveal that poor knowledge and practices related to the sick animal are associated with low vaccine coverage in these areas. We recommend a complete evaluation of anthrax vaccination among farmers in anthrax-prone districts to find out the challenges regarding vaccination.

Keywords:
Anthrax, anthrax vaccination, anthrax prone districts, PARB, DLS

13 - Abstracts (Day 3, November 16th, 2021 Session 2)

Zoonotic Diseases

Abstract Code: 2021-HIV/STI-856
Presenter Name: Tahira Kamal
Country: Pakistan
Presenter Email: tahirakhurram3@gmail.com
Authors: Dr. Ghulam Muhammad Ali, Dr. Naeem Riaz, Dr. S.M Andrabi, Dr. Zahida Fatima, Ms. Shan-e- Zahra, Ms. Zunera Fatima

Title: Detection of blaNDM-1 gene in Multidrug Resistant E.coli Isolated from Beef Samples Collected from Local Market of Islamabad

Background:
E.coli is one of the most prevalent foodborne pathogens, which showed resistance to commonly used antibiotics in recent studies. Carbapenem like imipenem and meropenem antibiotics are key antibiotics to treat multidrug-resistant bacteria. This study aimed to detect the blaNDM-1 gene in the E.coli strains isolated from beef samples collected from the local market of Islamabad.

Purpose:
Molecular identification of drug-resistant gene (blaNDM-1) in-ground beef samples

Methodology:
A total of 200 samples were collected from the ground beef samples. The phenotypic antimicrobial susceptibility assay against commonly used antibiotics including ciprofloxacin, levofloxacin, imipenem, meropenem, colistins, amoxycycline, clarithromycin, and imipenem EDTA, was performed through disk diffusion assay in isolated E.coli strains. The phenotypic detection of Extended-spectrum β lactamase in E.coli was checked through a double-disk synergy test (DDST). The blaNDM-1 was detected in the isolated E.coli strains through PCR.

Results:
25 out of 200 samples were found resistant to at least one drug in the antimicrobial class and considered multidrug-resistant (MDR).14 out of 25 were found phenotypically resistant to imipenem. Eight isolates were phenotypically positive for ESBL production, which were confirmed through a double-disk synergy test. The blaNDM-1 gene was also present in these eight E.coli isolates.

Conclusions:
The detection of the blaNDM-1 gene in the E.coli isolated from ground beef samples in the Islamabad region is a major threat to public health. There should be strict monitoring in the use of antibiotics in veterinary practices to avoid the emergence and dissemination of antibiotic resistance against key antibiotics, including Carbapenems, which might be a future threat to humans as one health concern.

Keywords:
E.coli, blaNDM-1, multidrug-resistant bacteria, beef samples, One-health

14 - Abstracts (Day 3, November 16th, 2021 Session 3)

TB & HIV

Abstract Code: 2021-OTH-881
Presenter: Rana Al Hamawi
Country: Jordan
Presenter email: ralhamawi@globalhealthdev.org
Authors: EMPHNET and IOM Research Group

Title: TB and HIV- Related Knowledge, Attitude, Practices, and Perceived Stigma Among Healthcare Workers

Background:
Jordan is labeled as a country with low prevalence of Tuberculosis and HIV.

Purpose:
To understand the knowledge, attitude, and practices of healthcare workers (HCWs) providing care to patients with HIV and TB in Jordan.

Methodology:
A concurrent embedded mixed quantitative/qualitative methods study has been conducted among HCWs who provide services to HIV and TB patients. HCWs' characteristics and number of patients seen, as well as questions relating to training, knowledge, attitudes, practices and stigma were assessed.

Results:
There is a clear deficit in knowledge of HCWs in areas related to both diseases. As for attitude, most HCWs had a positive attitude. The study showed suboptimal practices and stigma. Training provision to HCWs is inadequate. The HCWs' practices were fair and poor in 29.0% and 32.3% of HCWs, respectively. Almost one fifth of HCWs (4.5% of physicians, 25.9% of nurses, and 29.5% of other HCWs) reported that they have ever felt stigmatized because of their work around interacting with TB and HIV patients.

Conclusions:
HCWs should receive high quality, tailored, and regular training. Proper training on the clinical guidelines and on the existing infection prevention and control guidelines is of a paramount importance. Follow up on-job supportive supervisory visits will help to ensure improvement of practices.

Keywords:
HIV, Tuberculosis, Knowledge, Attitude, Practices

14 - Abstracts (Day 3, November 16th, 2021 Session 3)

TB & HIV

Abstract Code: 2021-OTH-879
Presenter: Majd Al-Soukhni
Country: Jordan
Presenter email: msoukhni@globalhealthdev.org
Authors: EMPHNET and IOM Research Group

Title: Evaluation of TB Surveillance System in Jordan

Background:
The Tuberculosis (TB) Surveillance System in Jordan has never been evaluated systematically and comprehensively.

Purpose:
To determine the gaps in the TB surveillance system and assess its usefulness and functionality.

Methodology:
A concurrent embedded mixed quantitative/qualitative methods study has been conducted to assess the TB Surveillance System in Jordan. A semi-structured questionnaire was developed based on the Updated CDC Guideline for Evaluating Public Health Surveillance System.

Results:
TB Surveillance System faces challenges in terms of deficiencies in human, equipment, and training. Not all TB surveillance System objectives were met in the past. The main gap in data quality is the use of different registration date formats. The performed analysis of collected data is neither thorough nor checked for outliers or data quality. The data that the system provides is not well utilized or analyzed thoroughly; accordingly, there were no actions taken as a result of the analysis and interpretation of the data from the TB Surveillance System. There is a delay in TB cases notification received from the parties involved in the TB Surveillance System, because it may have reached via mail or fax after a month of sending. The data providers' participation rate in the system is moderate. The TB patients do not sign a consent form before giving the data, and in many cases the patient does not know that they should give their consent prior obtaining the data from them.

Conclusions:
Based on identified gaps, the main recommendations include developing an electronic surveillance system software for data collection, notification and reporting, building the capacities of the TB healthcare provider regarding the TB diagnosis management, and properly applying the ethical considerations for the TB patients.

Keywords:
Tuberculosis, Surveillance, Evaluation

14 - Abstracts (Day 3, November 16th, 2021 Session 3)

TB & HIV

Abstract Code: 2021-OTH-880
Presenter: Adna Maiteh
Country: Jordan
Presenter email: amaiteh@globalhealthdev.org
Authors: EMPHNET and IOM Research Group

Title: Evaluation of HIV/AIDS Surveillance System in Jordan

Background:
The HIV/AIDS Surveillance Systems in Jordan has never been evaluated systematically and comprehensively.

Purpose:
To determine the gaps in the HIV/AIDS surveillance system and assess its usefulness and functionality.

Methodology:
A concurrent embedded mixed quantitative/qualitative methods study has been conducted to assess the HIV/AIDS Surveillance System in Jordan. A semi-structured questionnaire was developed based on the Updated CDC Guideline for Evaluating Public Health Surveillance System.

Results:
The usefulness of the system was rated as low. HIV/AIDS key informants are not aware if the system data were used to determine priorities, identify those at risk, determine HIV/AIDS risk factors, and evaluate prevention and control measures. Although, the HIV case definition is documented in the Epidemiological Surveillance Guideline in Jordan, some HIV/AIDS staff are not aware of the case definition or the guideline. The quality of the data is low. There is an inconsistency between the numbers of cases for some years; in addition, there are mistakes and errors in some calculations, and some missing data (blank) have been observed.

Conclusions:
The HIV/AIDS Surveillance System in Jordan is not well structured. There is a major need to improve many issues related to the National AIDS Program itself, and the surveillance system including stakeholders' involvement, purpose and operation, resources, usefulness, attributes, and ethical considerations.

Keywords:
HIV, AIDS, Surveillance, Evaluation

14 - Abstracts (Day 3, November 16th, 2021 Session 3)

TB & HIV

Abstract Code: 2021-EOH-703
Presenter Name: Noureddine Sakhri
Country: Morocco
Presenter Email: sakhriph@gmail.com
Author: Dr. Noureddine Sakhri

Title: Cascade of HIV Prevention, Care and Treatment Services in Morocco in 2019

Background:
The HIV care cascade is a way to show the proportion of people living with HIV (PLHIV) engaged at each stage of HIV care. Analyzing an HIV testing, care, and treatment cascade provides a framework for evaluating and improving service delivery.

Purpose:
The objective of this study is to analyze the continuums of HIV prevention, care and treatment services at the national level in Morocco for the period from 2015 to 2019.

Methodology:
This is a retrospective study, which concerned the reference centers for HIV care in Morocco carried out in 2019. Three types of cascade of HIV prevention and care were studied: a transversal cascade at the national level in 2019, a longitudinal cascade for newly diagnosed PLHIV between 2015 and 2017, and a cascade of prevention of mother-to-child transmission of HIV (PMTCT) in 2016-2017. The study process included collecting the data needed to construct the cascade.

Results:
For the transversal cascade of the year 2019, the objectives of the three 90s were achieved except for the 1st 90 with a difference of 12%. For the longitudinal cascade after the start of treatment between 2015-2017: retention under treatment at 48 months was 83.3%, 83.8% at 36 months, and 91.0% at 24 months. The 48-month loss to follow-up rate was 12.5%; 7.0% at 36 months and 4.0% at 24 months. More than 90% of PLHIV started antiretroviral within three months of diagnosis. The 2016-2017 PMTCT cascade conducted on 13 pregnant women according to available data indicated the absence of transmission of HIV from mothers to their children.

Conclusions:
Our cascade study demonstrated a number of successes. Achievement of the three 90s except for the first objective of people who know their status, good retention of PLHIV in long-term treatment, and success of PMTCT especially since Morocco aims to validate the elimination of MTCT

Keywords:
Cascade, HIV, treatment, Morocco, 2019, PMTCT

Seventh EMPHNET Regional Conference
Amman, Jordan

14 - Abstracts (Day 3, November 16th, 2021 Session 3)

TB & HIV

Abstract Code: 2021-EOH-677
Presenter Name: Suaad Moghalles
Country: Yemen
Presenter Email: smughalles@gmail.com
Author: Dr. Abdualwahed Alsurory

Title: Prevalence and Factors Associated with Transfusion-Transmitted Infections Among Multi–Transfused Patients, Sana'a City-Yemen, 2019

Background:
Multi–transfused patients (MTPs) are at higher risk of transfusion-transmitted infections (TTIs) due to their frequent needs for blood transfusion. Nevertheless, little is known about the prevalence of TTIs among MTP and its associated factors in Yemen.

Purpose:
Determine prevalence of hepatitis B virus (HBV), hepatitis C virus (HCV), and human immunodeficiency virus (HIV) and its associated factors among MTPs.

Methodology:
A Cross sectional study was conducted at the Yemeni Society for Thalassemia and at Pediatric Leukemia Unit in Sana'a City. The 357 calculated sample size was increased to 405 to overcome any non-response. Using Probability Proportional to Size sampling, 80 Thalassemia patients (TPs), 240 Sickle cell anemia patients (SCAPs), and 85 Leukemia patients (LPs) were randomly selected. Data was collected through face-to-face interview with patients or their caretakers using a predesigned questionnaire covers demographic, socioeconomic characteristics and TTIs associated factors. Blood samples were drawn and tested for HBsAg, Anti HCV, HIV I & II using electrochemiluminescence immunoassay.

Results:
The overall prevalence of TTIs among MTPs was 13.1% and significantly highest (37.3%) among LPs. HBV (16.2%) and HCV (27.5%) prevalence also highest among LPs. Only two patients (0.04%) were found to be HIV positive among SCAPs. Co-infection with HBV and HCV was only found in five LPs. There was a significant association between TTIs and receiving > 30 blood units. Only 35% of MTPs found to be vaccinated against HBV

Conclusions:
Findings raise the alarm for the existence of the high risk of TTIs among MTPs, especially among LPs and with an increasing number of transfusions. Using advanced technology in blood screening and strict infection prevention during transfusion should be adopted. Rational use of blood/blood substitutes and ensuring MTPs vaccination against HBV was recommended.

Keywords:
Prevalence, Transfusion-transmitted infections, Multi–transfused Patients, Yemen

14 - Abstracts (Day 3, November 16th, 2021 Session 3)

TB & HIV

Abstract Code: 2021-EOH-612
Presenter Name: Yasser Ghaleb
Country: Yemen
Presenter Email: ysrghaleb@gmail.com
Authors: Dr. Aymn Alshahari, Dr. Majdi Ali, Dr. Mohammed Al Amad, Prof. Abdulwahed Al Serouri

Title: Transfusion Transmitted Infections: Prevalence and associated factors, National Blood Transfusion and Research Center, Sana'a Capital, Yemen

Background:
Yemen faces major challenges in ensuring the safety and availability of blood transfusion to meet the increased demand for blood transfusion due to the protracted conflict. However, Transfusion-transmissible infectious (TTIs) agents such as hepatitis B virus (HBV), hepatitis C virus (HCV), human immunodeficiency virus (HIV), syphilis, and malaria remain the greatest threats for blood transfusion safety in such fragile, conflict-affected and vulnerable settings.

Purpose:
To determine the magnitude of transfusion-transmitted infections (TTIs) among blood donors and its associated factors.

Methodology:
A cross-sectional study was conducted on 340 blood donors at the National Blood Transfusion and Research Center during November and December 2017. Data was collected through face-to-face interviews using a predesigned questionnaire that covered socio-demographic characteristics and associated factors with TTIs. Blood samples were drawn and tested for HBV surface antigen (HBsAg), HCV antibodies, HIV I&II by using electrochemiluminescence immunoassay technique and rapid immunochromatographic for screened syphilis and malaria antibodies. Multivariable logistic analysis was used for identifying associated factors.

Results:
The overall prevalence of TTIs was 8.8% where 2.5 %, 1.2%, 0.3%, 1.2%, 3.2% have HBV, HCV, HIV, syphilis and malaria respectively. HBV was significantly associated with a history of jaundice and cupping. Furthermore, urethero-vaginal excretion was significantly associated with syphilis, while malaria was significantly higher among donors from malaria-endemic areas. Nearly three-fourths of donations were replacement donors who had significantly higher TTIs prevalence than the voluntary donors: 10.4% vs. 3.3%, OR 3.4 (CI: 1.1-11.6).

Conclusions:
Although the prevalence of TTIs is low, it remains an important problem in blood transfusion. Therefore, the establishment of TTIs surveillance system, improving donor recruitment procedures, and increasing the proportion of regular and voluntary donations with using more sensitive screening methods is recommended.

Keywords:
Transfusion transmitted infections, Blood donors, HIV, Syphilis, Malaria, Conflict

14 - Abstracts (Day 3, November 16th, 2021 Session 3)

Non-communicable Diseases

Abstract Code: 2021-FWBD-829
Presenter Name: Dr. Ansam AlHadidi
Country: Jordan
Presenter Email: ansamhadidi22@gmail.com
Authors: Dr. Ansam AlHadidi, Dr. Ashraf Aqel, Dr. Yousef Al-Gaud

Title: Prevalence of Obesity Among Adults in Jordan: A National Survey

Background:
Obesity is a national and global public health problem regarding morbidity, mortality, and economic burden. In 2014, 5% of the deaths worldwide were attributable to obesity, with an estimated economic impact of 2.8% of the global gross domestic product. A survey in 2008 showed a high prevalence of overweight and obesity among adults in Jordan.

Purpose:
To determine the prevalence of obesity, assess its trends, and determine its associated factors and associated comorbidities.

Methodology:
A multipurpose national household survey was conducted among Jordanian adults over four months in 2017. Data were collected using a structured validated questionnaire. Obesity was defined according to the International Diabetes Federation (IDF) criteria.

Results:
This study included a total of 4,056 persons (1193 men and 2863 women). Their age ranged from 18 to 90 years, with a mean of 43.8 (+14.2) years. According to the IDF criteria, the age-standardized prevalence of obesity was 60.4% among men and 75.6% among women. After adjusting for age, the odds of obesity in 2017 were twice that odds in 2008 in men (OR= 1.98) and women (OR = 1.96). In the multivariate analysis, age, place of living, and marital status were significantly associated with obesity among men and women. Obesity was significantly associated with increased odds of diabetes mellitus (OR = 2.1 for men and OR = 2.9 for women), hypertension (OR = 2.4 for men and OR = 2.5 for women), elevated triglyceride levels (OR = 2.5 for men and OR = 4.2 for women), and low High-Density Lipoprotein (OR = 2.2 for men and OR = 2.1 for women) after adjusting for age.

Conclusions:
Obesity in Jordan is high and is associated with other metabolic abnormalities. Well-defined programs to manage, control, and prevent obesity and intersectoral action are urgently required to reverse current trends in Jordan.

Keywords:
Obesity; survey; waist circumference; metabolic abnormality

14 - Abstracts (Day 3, November 16th, 2021 Session 3)

Non-communicable Diseases

Abstract Code : 2021-FWBD-822
Presenter Name: Dr. Bayan Sarsour
Country: Jordan
Presenter Email: bayan_sarsour@yahoo.com
Authors: Dr. Bayan Sarsour, Dr. Ashraf Aqel, Dr. Yousef Khader

Title: Behavioral and Emotional Problems Among Jordanian and Syrian Refugee Children in Non-Camp Settings

Background:
Refugee children have an increased risk of physical and psychological illness Data on behavioral and emotional problems among Jordanian and Syrian refugee children in non-camp settings are scarce.

Purpose:
This study aimed to assess the behavioral and emotional problems among Syrian schoolchildren refugees living outside camps in Jordan and their Jordanian counterparts.

Methodology:
A cross-sectional study was conducted among Syrian and Jordanian school children, aged 12-17 years, studying in the same schools in four Jordanian cities with the highest density of Syrian refugees. A self-reported questionnaire was used to collect information about the socio-demographic characteristics of children. Strengths and Difficulties Questionnaire (SDQ) was used to measure behavioral and emotional problems among these children.

Results:
This study included a total of 1878 Jordanian adolescents (45.6% males and 54.4% females) and 1773 Syrian refugee adolescents (43.9% males and 56.1% females). The parents of Syrian adolescents were significantly less educated and had significantly lower total family income than Jordanian parents. More than half of Jordanian and Syrian adolescents had peer relation problems (53.6% and 55.5% respectively), 36.9% of Jordanian and 35.5% of Syrian adolescents had hyperactivity/inattention problems, 44.8% of Jordanian and 47.6% of Syrian adolescents had conduct problems, and 30.8% of Jordanian and 32.0% of Syrian adolescents had emotional symptoms. On the other hand, 43.0% of Jordanian and 42.5% of Syrian adolescents had prosocial behaviors. In the multivariate analysis, Jordanian and Syrian children differed significantly in emotional symptoms and peer relationship problems. Compared to Jordanian children, Syrian children were less likely to experience emotional problems (OR = 0.81; p = 0.042) and peer relationship problems (OR = 0.80; p = 0.025).

Conclusions:
Emotional and behavioral problems are common among Syrian refugee schoolchildren as well as Jordanian schoolchildren. They are all in need of urgent psychosocial support.

Keywords:
emotional problems, behavioral problems, schoolchildren, Syrian refugees, Jordanian

14 - Abstracts (Day 3, November 16th, 2021 Session 3)

Non-communicable Diseases

Abstract Code: 2021-FWBD-610
Presenter Name: Ghamdan Gamal Alkholidy
Country: Yemen
Presenter Email: ghamdangamal@gmail.com
Authors: Dr. Labiba Anam, Dr. Abdulwahed Al Serouri, Dr. Chaoyang Li

Title: Non-Communicable Diseases Household Survey Data Analysis, Sana'a City, 2017

Background:
Non-Communicable Diseases (NCDs) kill 41 million people each year, equivalent to 71% of deaths globally. The burden of NCDs is rising faster in developing countries of the Middle East. The morbidity and mortality of NCDs are still not well-studied in Yemen.

Purpose:
To describe the epidemiology of NCDs in Sana'a City, 2017.

Methodology:
Raw data of house-to-house survey that was conducted by the Ministry of Public Health and Population in 2017 were analyzed. Data were collected from household heads who were asked if any household member had one of the following five NCDs: Hypertension (HTN), Diabetes (DM), Bronchial Asthma (BA), Mental Disorders (MD), and Epilepsy. Data was entered and analyzed using Epi info 7.2. For calculations of prevalence, 2017 projections from the 2004 census were used.

Results:
Total households surveyed were 241,310 (1,592,646 household members) of them 59,061 households (24.5 %) had 70,178 patients who had at least one of NCDs. Overall prevalence NCDs was 4.4%. Disease specific prevalence was: HTN (2.3%), DM (2.2%), BA (0.4%), MD (0.27), and Epilepsy (0. 19%). The overall NCD prevalence was significantly higher among females than males: 5.1% vs. 3.8 %, OR 1.35 (95% CI:1.33-1.35) as well as for HTN 3.1% vs 1.6%, OR 1.94 (CI:1.90-1.98); DM 2.3% vs. 2.1%, OR 1.11 (95% CI:1.09-1.13) and BA 0.5% vs. 0.3%, OR 1.56 (CI:1.49-1.65). In contrast, prevalence of MD was significantly higher among males than females: 0.35% vs. 0.16%, OR 2.2 (CI: 2.06-2.31) . For all NCDs, prevalence was progressively increasing with age. Nearly 18% of patients had more than one of NCDs, 35.2% of HTN patients had also DM.

Conclusions:
The reported data reflect only the tip of the iceberg as findings are based on self-reported diagnosed cases rather than standardized measures. For obtaining high-reliability data, NCDs Stepwise Survey and establishing NCDs surveillance system are recommended.

Keywords:
NCDs, Survey, Yemen

14 - Abstracts (Day 3, November 16th , 2021 Session 3)

Non-communicable Diseases

Abstract Code: 2021-FWBD-715
Presenter Name: Mohammed Sameer
Country: Iraq
Presenter Email: Mohammed.Samir1300a@comed.uobaghdad.edu.iq
Author: Dr. Mohammed Sameer

Title: Impact of COVID-19 on the Non-communicable Diseases (NCDs) Programs Provided in the Primary Healthcare Centers (PHCs) in Baghdad, 2020

Background:
NCDs have globally shown an increasing impact on health status with disproportionately higher rates in developing countries. During the COVID-19 pandemic, healthcare workers, equipment, and facilities have been reallocated to address the influx of patients with the disease.

Purpose:
To determine the impact of COVID-19 on the performance of NCDs programs implemented in the PHCs in Baghdad 2020 compared to 2019 and identify the potential causes of the changes.

Methodology:
This desk review study was conducted on a systematic sample of 20 PHCs having NCDs units in Baghdad from April to June 2021 by comparing certain NCDs performance indicators of 2019 and 2020.

Results:
The 20 PHCs served around 736,536 inhabitants. There were 92 medical doctors and 212 nurses and paramedics working in NCDs units in these centers. All provided services were affected in 2020. The screening and primary care for the chronic respiratory disease were the most affected. The number of patients had decreased to zero during 2020, followed by ophthalmic health services with a -80.6% percent change. Around 34% of the working staff was fully devoted to the pandemic services, 61% provided mixed services, and only 5% continued providing NCDs services. All training services had completely stopped in 2020. Provision of NCDs educational materials had decreased by 45%-100% for various services. Similarly, the awareness campaigns were reduced by 20-100% for various services. The main reasons for the services decline were the closure of outpatient services as per government directive, closure of outpatient disease-specific consultation clinics, and a decrease in outpatient volume due to patients' reluctance to attend the centers. Only a few centers adopted forms of telemedicine.

Conclusions:
COVID-19 pandemic had drastically affected the NCD services in Iraq. Having contingency plans and the adoption of telemedicine can mitigate collateral damage associated with the pandemic.

Keywords:
Keyword: Iraq, Non-Communicable Diseases, COVID-19, Primary Healthcare Center

14 - Abstracts (Day 3, November 16th, 2021 Session 3)

Non-communicable Diseases

Abstract Code: 2021-NCD-648
Presenter Name: Noor Yaseen
Country: Palestine
Presenter Email: noorzahi-2015@hotmail.com
Author: Dr. Noor Yaseen

Title: The Prevalence and Predictors of Depression Among the Palestinian Community During COVID-19

Background:
Aggressive quarantine and lockdown measures were implemented as protective public health actions during the coronavirus disease 2019 (COVID-19) pandemic. Assessing the psychological effects associated with these measures is an important attempt to inform local policymakers in an early stage. Yet little is known about these effects, specifically depression, among the Palestinians.

Purpose:
Assess the prevalence and predictors of depression among the Palestinian community during this pandemic.

Methodology:
A cross-sectional web-based survey throughout social media using an online questionnaire. The validated and standardized depression, anxiety, and stress scale (DASS) used to measure depression severity. A snowball technique recruiting the general public living in Palestine was conducted. A multinomial logistic regression model was developed to predict depression severity.

Results:
2,819 respondents filled out the questionnaire. The mean age was 29.47 (SD=10.97), 72.6% were female, 83.5% live in the West Bank. Depression prevalence was (57.5%; n = 1,621). Significant associations were found between depression severity and each of age, sex, social status, residency, geographic area, educational level, monthly income, smoking, and the presence of a high-risk individual (P-value <0.05) Depression severity was negatively associated with age {mild/moderate degree [OR (95% CI) = 0.98 (0.97–0.99)] and severe/extremely severe [OR (95% CI) = 0.96 (0.94–0.97)]}. Males were less likely to have higher depression than females {mild/moderate degree [OR (95% CI) = 0.69 (0.57–0.85)] and severe/extremely severe [OR (95% CI) = 0.52 (0.40–0.86)]}.However, those who reported having inadequate food supply,lesser monthly incomes and single persons were more likely to have a higher degree of depression.

Conclusions:
Depression prevalence was found to be 57.5%. Whereas in Italy it was 17.3%, in Spain it was 19%. in the UK it was 22.12%. Age, high-risk individual, and monthly income showed an inverse relationship with depression. Those results are similar in studies in Italy, Spain, and the UK. Implementing comprehensive interventions considering socioeconomic disparities, vulnerability, and inequities, is crucial to emerge from this crisis in Palestine.

Keywords:
COVID-19, depression, Palestine, quarantine, lockdown COVID-19, depression, Palestine, quarantine, lockdown

14 - Abstracts (Day 3, November 16th, 2021 Session 3)

Non-communicable Diseases

Abstract Code: 2021-FWBD-683
Presenter Name: Sara Abu Khudair
Country: Jordan
Presenter Email:sara.a.khudair@gmail.com
Authors: Dr. Hana Morrissey, Dr. Janos Sandor, Dr. Ziad El-Khatib, Prof. Yousef S Khader

Title: Factors Associated with Suboptimal Adherence to Hypertensive Medications Among Syrian Refugees – Cross-Sectional Study at the Zaatari Camp, Jordan

Background:
The growing caseload of chronic conditions requiring long-term medication use, such as hypertension, among Syrian refugees in Jordan raises concerns about the extent to which patients are taking their medications as prescribed. The lack of evidence on this issue represents a missed opportunity to reduce preventable hypertension-related complications, mortalities, and healthcare costs.

Purpose:
To assess the level of medication adherence and associated factors among Syrian hypertensive patients in Jordan.

Methodology:
This cross-sectional study included 180 randomly selected Syrian refugees diagnosed with hypertension residing in Zaatari camp, Jordan. The Adherence to Refills and Medications Scale (ARMS) was used to assess adherence to antihypertensive medications. Additional data were collected on sociodemographics, therapy-related factors, patients' behaviors and knowledge of hypertension disease and therapy, and health system-related factors. Multivariate linear regression was used to assess the association between adherence scores and other variables.

Results:
The mean (SD) of ARMS scores was 15.7 (2.9). Based on ARMS scores, 22.8% were adherents, and 77.2% were non-adherents. Multivariate analysis showed that newly initiated therapy (≤ 2 years) and illiteracy were strong predictors of lower adherence with $p < 0.001$ and $p = 0.012$, respectively. Other variables that were significantly associated with lower adherence included discontinuation of antihypertensive medications due to side effects ($p = 0.032$), irregular availability of free antihypertensive medications dispensed by non-governmental organizations (NGOs) ($p = 0.024$), and dissatisfaction with health services ($p = 0.022$).

Conclusions:
Suboptimal adherence to antihypertensive medications remains a substantial unmet need among Syrian refugees in Jordan. As illiteracy appears to impact adherence levels negatively, educational interventions that promote favorable health behaviors through auditory and visual aids are needed to better engage people with limited literacy skills. Strengthening the pharmaceutical supply chain at various levels is strongly recommended to respond quickly to changes in demand and prevent an indirect negative impact on adherence levels.

Keywords:
Syrian refugees, medication adherence, hypertension, illiteracy, medications availability, Zaatari camp

14 - Abstracts (Day 3, November 16th, 2021 Session 3)

Non-communicable Diseases

Abstract Code: 2021-FWBD-668
Presenter Name: Zeina Al Sadder
Country: Jordan
Presenter Email: z.alsadder@JBCP.jo
Author: Dr. Reem Alajlouni

Title: Impact of Educational Sessions on Breast Cancer Awareness and Knowledge in Jordan

Background:
In Jordan, breast cancer is the most common cancer overall accounting for 20.7% of new cancer cases with a mortaility rate of 12.7% . To respond to the increasing incidence, Jordan Breast Cancer Program (JBCP) was founded to lead and improve national early detection and screening efforts by ensuring availabity and access to quality early detection and screening services and increasing awareness about breast cancer. Several approaches have been considered, particularly community based approaches including one to one teaching sessions and one to group. JBCP delivers educational lectures throughout Jordan through its well-prepared community health educators in order to raise awareness, change attitudes, and imbed practices of breast cancer screening and early detection.

Purpose:
To assess the effectiveness of the breast cancer lectures approach in Jordan

Methodology:
Data was collected from pre- and post-educational sessions evalautions for the years of 2011-2015 in order to assess the improvement of communities' knowledge. A total of 11,873 questionnaires were reviewed and the data were entered, coded, and analyzed by the SPSS program.

Results:
Results have shown that after the educational session, 68% of participants knew how to perform self-breast examination and 83% had planned to visit a healthcare provider to undergo clinical breast examination, while 66% indicated that they will undergo mammogram. The knowledge diference was assessed and there was 41% increase in the partcipant's knowledge of breast cancer and its screening and early detection methods.

Conclusions:
There is an improvement in the participant's knowledge as a result of the lectures conducted by JBCP.While it's improtant to continue our efforts in delivering lectures and raising awareness, it's equally important to alter behaviours and transform that knowledge into practice.

Keywords:
breast cancer, NCDs, community awareness, outreach.

15 - Abstracts (Day 4, November 17th, 2021 Session 1)

COVID-19

Abstract Code: 2021-EOH-679
Presenter Name: Marha Kamoona
Country: Iraq
Presenter Email: kdr.marha@yahoo.com
Author: Dr. Deepak Kumar

Title: Assessment of Preparedness for COVID-19 Pandemic in Schools / Iraq, Baghdad Al-Rusafa Health Directorate/Al-Rusafa District/ 2021

Background:
Following the international spread of novel coronavirus (SARS-COV-2) or COVID-19 pandemic, Iraqi government took several steps to prevent community transmission, including indefinite closure of schools as a measure to safeguard schoolchildren from COVID-19.
The key rationales behind this decision were the insufficient preparedness level within schools to prevent infection and lack of appropriate vaccines for children.

Purpose:
Researchers assessed COVID-19 preparedness levels in schools in Al-Rusafa district, Baghdad to support the Iraqi government through recommendations on preparedness improvement in schools in Iraq before opening.

Methodology:
An observational study design was conducted to assess schools. Stratified sampling was performed to make the sample more representative. We did stratifying the schools into three categories based on sex, level (primary, secondary), and administration (public, private). Study population comprised of all students and teachers in selected sample. The assessment was done retrospectively for 3 months from 31/5/2021. Data was collected through face to face interviews and analyzed using Microsoft Excel; tables and pie charts were used to display results.

Results:
Assessment was completed in 40 schools: 20 primary schools (50%), 10 high schools (25%), 6 intermediate schools (15%), and 4 secondary schools (10%). The assessment concluded that all schools did not have a robust screening system to record students infected with COVID-19. Another key finding was that only 10% of face masks users inside the schools reflected low-level school participation in preparing schools against COVID-19 pandemic. Further, referral system to transport any sick person to appropriate health facility is not present or not functioning in 63% of schools. Only 35% of schools record student absenteeism due to any reason.

Conclusions:
The research discusses several actions and requirements that should be reviewed and put in place to prevent COVID-19 in schools and into community & recommends establishing a robust screening system to record details of students infected with COVID-19.

Keywords:
COVID-19, Al-Rusafa District, spread of Covid-19,schools

15 - Abstracts (Day 4, November 17th, 2021 Session 1)

COVID-19

Abstract Code: 2021-EOH-705
Presenter Name: Firas Adnan
Country: Iraq
Presenter Email: Ferras.Adnan1300a@comed.uobaghdad.edu.iq
Authors: Dr. Amer Baqir, Prof. Faris Lami

Title: Seroprevalence of COVID-19 Among Healthcare Workers in Ibn-Albytar Center for Cardiac Diseases, Baghdad, Iraq, 2020

Background:
The prevalence of asymptomatic infections in the community is important to estimate the actual infection, fatality rate, and the extent of achieving herd immunity. Healthcare workers are at increased risk of contracting COVID-19. Estimating the prevalence of previous asymptomatic infection among healthcare workers will guide shaping policies to better protect this group and control the pandemic.

Purpose:
To estimate the seroprevalence of COVID-19 among healthcare workers at Ibn-Albytar Center for Cardiac Diseases, Baghdad, Iraq, 2020.

Methodology:
A cross-sectional study was conducted in a hospital setting, Ibn-Albytar Center for Cardiac Diseases, not directly involved in managing COVID-19 patients during November and December 2020. A questionnaire was developed to gather demographic variables, history, determinants of contracting COVID-19 infection, and training and use of the personal protective equipment that was filled through direct interview. All healthcare workers had COVID-19 rapid antibody test (IgM-IgG Rapid test).

Results:
A total of 423 participants were enrolled in the study. Rapid test was positive in 125 (29.6%) of the participant; 86 (68.8%) of them had IgG, 1 (0.8%) had IgM, and 38 (30.4%) had both IgG and IgM. There were 115 (27.2%) healthcare workers who had a history of COVID-19 infection (PCR was used in 73.0% of them). The rapid test had a sensitivity of 72.6% and a specificity of 80.5%. A positive rapid test was significantly associated with a positive PCR test ($p<0.001$), having a history of COVID-19 infection ($P<0.001$), and contact with a confirmed case ($P<0.001$). A significant association was also found between COVID-19 infection and having contact with a confirmed case, smoking, household infection, and coworker infection ($P<0.05$).

Conclusions:
COVID-19 infection was common among hospital healthcare workers. Providing personal protective equipment and scheduling healthcare workers for vaccination once available is highly recommended.

Keywords:
COVID-19, seroprevalence, Iraq, tertiary center

15 - Abstracts (Day 4, November 17th, 2021 Session 1)

COVID-19

Abstract Code: 2021-EOH-644
Presenter Name: Mohamad Said Almasri
Country: Jordan
Presenter Email: mihammedsaidalmasri@gmail.com
Author: Dr. Mohamad Said Almasri

Title: Risk Factors and Characterization of Post-COVID-19 Syndrome in Jordan

Background:
Controversial information exist about the sequelae of COVID-19 after recovery or post-COVID-19 syndrome (PCS). Whereas considerable studies have been done on COVID-19, proportionally, there is scarcity of literature addressing the PCS, particularly the risk factors causing this syndrome. Determining the prevalence, most common manifestations of PCS, and the possible related risk factors is an important issue.

Purpose:
The aim of this study was to detect the prevalence and the risk factors for the development of the post COVID-19 syndrome (PCS) as well to identify the symptoms and their relation to the socio-demographic and medical characteristics of patients who survive.

Methodology:
A cross-sectional, online questionnaire-based study was conducted. This questionnaire was posted to the Association of "My experience with COVID-19" in Jordan. Socio-demographic, as well as COVID 19 illness information was collected, from 657 COVID-19 recovered patients at least three months after illness started.

Results:
PCS prevalence was 71.9%, where the patient experienced, at least one PCS symptom. Most common symptoms including dyspnoea, fatigue, taste and smell impairment, cough, and depression. Six factors were found to be significantly increasing the risk of PCS (using OR, 95% CI); female, aging ≥30, DM, hypertension, respiratory disease, and neuro-psychological disturbance during illness. Those patients showed also a significantly higher rate of post-COVID-19 syndrome than their counter groups.

Conclusions:
The PCS prevalence is high in Jordan. Particularly among certain populations like females, age ≥30 years, having neuropsychological disturbance during illness and comorbidity; DM, hypertension, and respiratory diseases. In other words, those populations should be considered as a risk group for the PCS occurrence. Therefore, the COVID-19 infection treatment is not only during the episode but has to continue several months after the recovery of the patient.

Keywords:
COVID-19, Sequelae, epidemiology, risk factors, symptoms, Jordan

15 - Abstracts (Day 4, November 17th, 2021 Session 1)

COVID-19

Abstract Code: 2021-EOH-645
Presenter Name: Wehad Elsafi
Country: Sudan
Presenter Email: wehadel18@gmail.com
Author: Dr. Wehad Elsafi

Title: Analysis of COVID-19 Pandemic Data in Omdurman Locality, Khartoum State between July 2020 to March 2021

Background:
The first case of COVID-19 in Khartoum State was detected on 12th of March 2020, in Omdurman about 1,764 cases were reported and immediately field investigations were conducted by the Ministry of Health to identify and confirm additional cases.

Purpose:
As the outbreak of coronavirus COVID-19 progress, the analyses of epidemiological data are needed to increase situational awareness and to inform public health interventions.

Methodology:
Secondary data analysis of line-list of COVID-19 cases was conducted. Data was collected through the field epidemiology investigation coordinated by the Emergency and Epidemiology Department in Omdurman locality. COVID-19 suspected cases were reported through the sentinel sites and the call center at the Federal Ministry of Health. All the reported cases were entered in DHIS2 program and investigated by Rapid Response Teams (RRTs) at the locality. Information of each case investigated was documented using the COVID-19 case investigation form and COVID-19 line-list which captured socio-demographic characteristics, history of exposure to COVID-19, presenting symptoms, travel history and sample collection details, and contact tracing, data was analyzed using a Microsoft Excel.

Results:
A total of 1,764 suspected cases were reported and tested for COVID-19 of which 709 COVID-19 cases were laboratory-confirmed. The mean age of the confirmed cases was 42.9 years and the highest proportion of COVID-19 cases and deaths occurred in the age group >60 years (8%) and 50-60 years (3.4%), respectively. More females (57.9%) were affected than males (42.1%). Most of the cases were symptomatic 607 (85.6%) and presented with key symptoms such as fever (450/709; 63.4%), cough (436/709; 61.5%), sore throat (330/709; 46.5%), and difficulty in breathing (261/709; 36.8%). The case fatality rate (CFR) is 3.4% and most of the confirmed cases 677 (81.3%) reside in urban places. The epidemic curve shows a propagated pattern of transmission with the outbreak reaching its peak on 13 December 2020 with 27 confirmed cases reported in a day. There are asymptomatic confirmed cases (102/709; 14.4%). A high mortality rate was reported in cases age >60 years (high-risk group) at 26.2% and among adults, 21-30 years is 20%.

Conclusions:
Although the linelist provide very important information about the outbreak, however, it was difficult to obtain at the beginning of an outbreak due to the lack of information management system for COVID-19 data. The field investigation by RRTs has found high transmission of COVID-19 infection among urban residents with high mortality among females in the older age group in the locality. Interventions included intensified risk communication and use of community structures to ensure compliance with public health COVID-19 preventive protocols, including enforcing infection prevention and control compliance, particularly among high-risk groups are needed.

Keywords:
COVID-19, HIGH RISK

15 - Abstracts (Day 4, November 17th, 2021 Session 1)

COVID-19

Abstract Code: 2021-EOH-699
Presenter Name: Abdullah AlSayafi
Country: Saudi Arabia
Presenter Email: hiiabady@gmail.com
Author: Dr. Abdullah Alsayafi

Title: Outbreak of COVID-19 Among Healthcare Workers at a Diabetes Center, Al Ahsa, Saudi Arabia - April 2020

Background:
On 2nd of April 2020, the Field Epidemiology Training Program (FETP) was asked by a Diabetes Mellitus Center, Facility A, in Al-Ahsa to investigate a confirmed case of COVID-19 in a health care worker. Diabetes patients are at increased risk for serious complications from COVID-19.

Purpose:
We sought to identify any additional cases, the source of infection and mode of transmission, and implement mitigation measures to prevent further transmission. At the time of the investigation, few COVID-19 cases had been identified in the region.

Methodology:
We reviewed medical charts and other available data on cases in the infection control department, the public health department, and COVID-19 center. We interviewed cases about their health status and possible sources of infection. We analyzed data using descriptive statistics.

Results:
All staff (45) at Facility A were tested for COVID-19; 2 (4%) tested positive. The first case was a nurse that reported attending a nursing conference in Riyadh several days prior to symptom onset. She was the first recognized case of COVID-19 in Facility A, so we classified her as the index case. We identified one more case that reported using shared cupboard clothes, the same coffee machine, and chatting with the index case in a breakroom without using personal protective equipment (PPE). Both cases reported wearing PPE during patient care. Both cases survived.

Conclusions:
We found evidence of person-to-person transmission between cases while socializing at work and no evidence of transmission from health care workers to patients. We identified potentially risky practices in Facility A, although none were related to patient care. We helped Facility A develop additional policies to reduce the risk of COVID-19 transmission among staff, even when not providing direct patient care. Facility A was closed during the investigation and reopened after applying all prevention measures. No additional cases occurred.

Keywords:
COVID-19, SARS-Cov2, Diabetes, Outbreak, Al-Ahsa, Saudi Arabia

16 - Abstracts (Day 4, November 17th, 2021 Session 2)

COVID-19

Abstract Code: 2021-EOH-872
Presenter Name: Ahmad Amirnajad
Country: Afghanistan
Presenter Email: dews.westregion@gmail.com
Authors: Dr. Khawja Mir Islam Saeed, Dr. Mohammad Hafiz Rasooly

Title: Accuracy of Rapid Diagnostic Antigen Test Among Asymptomatic Close Contacts with Confirmed SARS-COV 2 Infections in Herat Province, Afghanistan, in 2021: A cross-sectional Study

Background:
Early detection and isolation are the key strategies for the containment of the COVID 19 pandemic in low resources settings including Afghanistan where access to the vaccine is limited. These strategies could reduce the burden on the health care system which is already weak due to conflicts and war. The first COVID 19 case was detected in the country in Herat province close to Iran. Currently, rapid antigen tests and RT-PCR have been used for detections of COVID 19 cases in Herat province.

Purpose:
To assess the accuracy of the rapid antigen test among asymptomatic close contacts of individuals confirmed for covid 19 in Herat province.

Methodology:
This was a cross-sectional study conducted by the surveillance contact tracing teams in Herat province. The teams line listed 200 asymptomatic close contacts, and two separate nasopharyngeal specimens were collected. We used the rapid antigen test (Biosensor) on the 4th-7th days of the contact, and the 2nd specimen was sent to the reference lab for RT-PCR testing. Descriptive statistics were conducted. The sensitivity and specificity of the rapid antigen tests were compared with RT-PCR test results.

Results:
The median age of the contacts was 35 (ranged 11-90), and 138 (70%) of the contacts were female. Out of 196 contacts tested with the RT-PCR, 105 (53%) were confirmed for SARS-COV-2 infection, while only 30(15%) were confirmed by rapid antigen test. It indicates a sensitivity of 20.1%. However, the specificity of the rapid antigen test was high (90%).

Conclusions:
The sensitivity of the rapid antigen tests was quite low to confirm covid 19 cases among asymptomatic close contacts. Therefore, if resources allow, RT-PCR is still the best choice with a high sensitivity rate for early detection of the Covid 19 asymptomatic close contacts. Further study with a large sample size should be considered.

Keywords:
Rapid, Antigen, test, SARS-COV 2, Diagnostics, close contacts

16 - Abstracts (Day 4, November 17th, 2021 Session 2)

COVID-19

Abstract Code: 2021-EOH-622
Presenter Name: Aqeel Alobaidi
Country: Iraq
Presenter Email: dr.akeelismaeel79@gmail.com
Authors: Dr. Hanan Khaleel, Prof. Faris Lami

Title: COVID-19 Rapid Tests in Iraq: How Valid Were They?

Background:
Rapid SARS-CoV-2 Ab tests were widely distributed and used at the beginning of the COVID-19 pandemic. However, in Iraq, no study evaluated the field accuracy of the rapid antibody test.

Purpose:
To assess the validity of the rapid test (Dutch-made BIOZEK) used in Iraq through calculating the sensitivity, specificity, positive and negative predictive values on a sample of suspected COVID-19 cases.

Methodology:
A descriptive cross-sectional study. We included 1,000 individuals who were either symptomatic or had had contact with a confirmed COVID-19 case. A structured questionnaire was filled in through direct interviews with the participants. Both rapid antibody tests and PCR tests were done simultaneously. The sensitivity, specificity, and positive and negative predictive values were calculated.

Results:
The sensitivity was 14% for IgG, 3.2% for IgM, and 5.7% for both. The specificity was 74.5% for IgG, 95.1% for IgM, and 91.3% for both. The positive predictive value was 32.8% for IgG, 36.6% for IgM, and 37% for both. The negative predictive value was 49.3% for IgG, 52.5% for IgM, and 52.1% for both.

Conclusions:
SARS-CoV-2 antibody test should not be used for diagnosing COVID-19 patients or in screening programs. However, it can be used for epidemiological surveys purposes.

Keywords:
COVID-19, Rapid antibody test, Screening, Iraq

16 - Abstracts (Day 4, November 17th, 2021 Session 2)

COVID-19

Abstract Code: 2021-OTH-617
Presenter Name: Shamaila Usman
Country: Pakistan
Presenter Email: alamshamaila15@gmail.com
Author: Dr. Nadia Noreen

Title: Descriptive Analysis of Health Screening for Covid19 at Points of Entry of Pakistan According to CDC Guidelines from February 20 - March 21

Background:
Points of Entry of Pakistan serve as key conduits for international travel, transport and trade. It is responsible to control the international spread of diseases at points of entry which include 10 Internationals Airports, 03 Seaports and 06 Ground Crossings. In alignment with National Action Plan 2020 for Covid-19, the department has taken concrete steps in short span of time to cope up the pandemic.

Purpose:
To reduce the International spread of Covid 19 by strengthening Points of Entry.

Methodology:
A descriptive study on CDC (Centre for Disease Control and Prevention) Guidelines for Health Screening was conducted at Directorate of Central Health Establishments from March 2021 to May 2021. The CDC Guidelines are based on 11 attributes, to be implemented for Covid 19 health screening at POE. It includes legal and regulatory bodies to detain as suspect, Isolation and coordination at POEs, funds for screening, quarantine facilities with provision of necessities and communication channels to quarantined travelers, referral healthcare facilities for POEs, protocols for primary and secondary screening, capacity building at POEs, supply of PPE and screening tools, isolation areas, and provision of basic facilities at POEs. Data was collected using both Qualitative and Quantitative methods. Questionaries were filled online, and in-depth interviews of Incharges and Quarantine assistants of Points of Entry were done. Analysis was done of traveler's surveillance management information system of Central Health Establishment.

Results:
Attributes of Health screening as per CDC guidelines had been addressed and well implemented at POEs by Central health Establishment under flagship of MONHRS&C. Total Health screening of Inbound travelers was 4,088,119 . With 0.6% positivity rate, 415 suspects referred to Hospitals . Total international flights screened were 19,130.

Conclusions:
Preparedness and Response of Points of Entry for Covid 19 is in line with National Action Plan of Government of Pakistan and IHR 2005.

Keywords:
Points of Entry. Health screening, National action Plan, Isolation, Quarantine

16 - Abstracts (Day 4, November 17th, 2021 Session 2)

COVID-19

Abstract Code: 2021-OTH-841
Presenter Name: Salma Afifi
Country: Egypt
Presenter Email: afifisalma1@gmail.com
Authors: Dr. Abdelaziz Barkia, Dr. Alaa Eid, Dr. Falah Abdul-kader Saaed, Dr. Hajer Letaief, Dr. Hana Ahmad Taha, Dr. Hanaa Abu El Sood, Dr. Hashaam Akhtar, Dr. Hiba Abdulrahman Rashak, Dr. Khwaja Mir Islam Saeed, Dr. Maisa Elfadul, Dr. Mohamed Hassany, Dr. Mohammed Sameer Hlaiwa, Dr. Mohannad Al Nsour, Dr. Nissaf Ben Alaya, Dr. Reema Adam, Dr. Salma Afifi, Dr. Sami S. Almudarra, Prof. Yasser Ghaleb, Prof. Aamer Ikram, Prof. Faris Lami, Prof. Fazal Ul-Ulrahman, Prof. Yousef S. Khader

Title: Public Health Workers' Knowledge, Attitude, and Practice Regarding COVID-19: The Impact of Field Epidemiology Training Program in the Eastern Mediterranean Region

Background:
Globally, there is growing need for public health professionals skilled in preventing and responding to the surge of emerging and re-emerging infectious diseases. This is particularly important to the Eastern Mediterranean countries that are facing emergencies in addition to the increased public health risks of unprecedented scale during COVID-19 pandemic. Public health professionals are instrumental in responding to COVID-19 pandemic in terms of detecting and monitoring new cases, conducting investigations, tracing contacts, ensuring patients are being tested, applying isolation and quarantine protocols, providing up-to date information, educating community, and producing statistics and models to track disease progression.

Purpose:
This study aimed to compare knowledge, attitude, and practice (KAP) regarding COVID-19 between public health workers (PHWs) who attended Field Epidemiology Training Program (FETP-trained) and those who did not attend FETP (non-FETP trained).

Methodology:
Multi-country cross-sectional survey was conducted among PHWs who participated in COVID-19 pandemic in ten countries in the EMR. Online questionnaire that included demographic information, knowledge, attitude, and practices regarding COVID-19 pandemic was distributed among HCWs. Scoring system was used to quantify the answers; bivariate and multivariate analysis were performed to compare FETP-trained with non-FETP trained PHWs.

Results:
Overall, 1,337 PHWs participated, with 835 (62.4%) <40 years of age, and 851 (63.6%) males. Of them, 423 (31.6%) had FETP, including 189 (44.7%) had advanced level, 155 (36.6%) intermediate and 79(18.7%) basic level training. Compared to non-FETP trained, FETP trained were older, having higher knowledge, attitude, and practice scores. FETP participation was low in infection control, and PH laboratories. KAP mean scores for intermediate level attendees are comparable to advanced level.

Conclusions:
FETP-trained are having better KAP than non-FETP PHWs. Expanding the intermediate level, maintaining the Rapid Response training, and introducing the laboratory component are recommended to maximize the benefit from FETP. Infection control, antimicrobial resistance, and coordination are areas where training should include.

Keywords:
knowledge, attitude, practice, COVID-19, Field Epidemiology Training Program

17 - Abstracts (Day 4, November 17th, 2021 Session 3)

COVID-19

Abstract Code: 2021-OTH-712
Presenter Name: Hanan Khaleel
Country: Iraq
Presenter Email: hanan_azawy2000@yahoo.com
Authors: Dr. Asaad Asaad, Dr. Asmaa Kannas, Dr. Magid Algunaid, Dr. Mohannad Al Nsoor, Dr. Nameer Ali, Dr. Riyadh Hilfi, Dr. Sinan Ghazi

Title: Iraq's Arbaeenia Mass Gathering Surveillance During the COVID-19 Pandemic Year, 2020

Background:
The Arbaeenia Mass Gathering in Iraq is one of the largest mass gatherings attracting millions of people from all over the world.

Purpose:
To describe the frequency and distribution of different health events encountered during the Arbaeenia mass gathering in Iraq, 2020.

Methodology:
Real-time surveillance was conducted in 152 temporary health outlets established on the roads in 11 governorates towards Karbala city (where the mass gathering takes place). It involved 306 surveillance officers specifically trained to use a questionnaire uploaded on a KoBo Toolbox. Besides basic demographic data, the questionnaire included information on acute communicable diseases, chronic diseases, and injuries.

Results:
A total of 330,607 visitors attended the health outlets; 99% were Iraqis. Sex distribution was similar across all age groups except in the group over 60 years old where males were more frequent than females. About 50% of the visitors had acute conditions, with flu-like illness constituting the majority (32.0%). Fever and sore throat were reported by 7,726 (4.7%), while fever and fatigue were reported by 3,748 (2.3%) of attendants to the health outlets. Around 58% had chronic conditions, and chronic joint pain constituted 69% of attendants with chronic complaints. No statistically significant difference was noted between the sexes for both acute and chronic conditions (p>0.05). In contrast, injuries were more common among males (58%) than females (42%) (p<0.05). Blisters following walking for long distances was the most encountered injury and reported by 69,476, constituted 94% of total injuries. Wound (n=1,751, 2.4%), lacerations (1,567, 2.1%), and burns (316, 0.4%) were also encountered in this event. Almost 50% of the injuries occurred in those aged 20-39 years.

Conclusions:
In 2020, the Arbaeenia Mass Gathering was considerably smaller than the events that occurred before the COVID-19 pandemic. It is important to organize the shrines in a way that keeps physical distancing and facilitates safe rituals.

Keywords:
Mass gathering, Iraq, Arbaeenia, COVID-19

17 - Abstracts (Day 4, November 17th, 2021 Session 3)

COVID-19

Abstract Code: 2021-EOH-665
Presenter Name: Manal Fahim
Country: Egypt
Presenter Email: fahimmanal@yahoo.com
Authors: Dr. Alaa Eid, Dr. Amel Naguib, Dr. Amira Mohsen, Dr. Hanaa Abu El Sood, Dr. Mohamad AbdelFatah, Dr. Mohamed Hassany, Dr. Nancy Elguindy, Dr. Salma Afifi, Dr. Wael H. Roshdy

Title: Co-infection with SARS-CoV-2 and Influenza A/H1 in a Patient Seen at Influenza Like-Illness Surveillance Site in Egypt: Case-report

Background:
Influenza like Illness (ILI) sentinel surveillance in Egypt started in year 2000 in 8 sentinel sites geographically distributed all over the country. In response to the COVID-19 pandemic, SARS-CoV-2 was added to the viral testing panel by PCR for the first two patients with ILI seen at one of the sentinel sites. We report the first SARS-CoV-2 and influenza A/H1 virus co-infection with mild symptoms detected through routine influenza like-illness surveillance (ILI) in Egypt.

Purpose:
This report aims at describing how the case was identified and the patient's demographic and clinical characteristics and outcomes.

Methodology:
The case was identified by the central public health laboratory staff who contacted the ILI sentinel surveillance officer at Ministry of Health. The case was contacted through phone call. Detailed information about patient clinical picture, course of disease and outcome. Patient's contacts were investigated for acute respiratory symptoms, disease confirmation and outcome.

Results:
Among 510 specimens collected from patients with ILI symptoms from October 2019 to August 2020, 61 (12.0%) were positive for COVID-19 and 29 (5.7%) were positive for influenza, including 15 (51.7%) A/H1, 11 (38.0%) A/H3, and 3 (10.3%) Flu-B. A 21-year-old female was confirmed as SARS-CoV-2 and influenza A/H1 virus co-infection. She had high fever of 40.2°C and mild respiratory symptoms that resolved within two days with symptomatic treatment. All her five family contacts had mild respiratory symptoms 2-3 days after exposure to the confirmed case and symptoms resolved without treatment or investigations.

Conclusions:
The case highlights the possible occurrence of co-infection in younger and healthy people who might resolve the infection rapidly. We emphasize the usefulness of surveillance system for detection of viral causative agent(s) of ILI and recommend the broadening of testing panel especially if it could guide case management.

Keywords:
Surveillance, influenza, SARS-CoV-2

17 - Abstracts (Day 4, November 17th, 2021 Session 3)

COVID-19

Abstract Code: 2021-EOH-682
Presenter Name: Amna Khairy
Country: Sudan
Presenter Email: Amnakh22@gmail.com
Authors: Dr. Ayman Ahmed, Dr. Dalya Eltayeb, Dr. Esra Mahgoob, Dr. Mawahib Jubara, Dr. Mohammad Nimir, Dr. Mohammed Ahmed

Title: Acceptability of COVID-19 Vaccination among Healthcare Workers in Sudan: A Cross-Sectional Survey

Background:
Following emergency approval of COVID-19 vaccines, several studies have investigated COVID-19 vaccine acceptance and hesitancy especially among healthcare workers (HCWs). Nevertheless, the acceptability of the COVID-19 vaccine by HCWs in Sudan remains unclear.

Purpose:
This study aims to investigate the acceptability of the COVID-19 vaccine and it's determinants among the HCWs.

Methodology:
A web-based cross-sectional study design was used to study COVID-19 vaccine hesitancy and its associated determinants. The semi-structured questionnaire was distributed electronically; data collection took place from March-April 2021. SPSS version 25 was used for analysis. Frequency tables was done for categorical variables. Means (M) and standard deviations (SD) were estimated for the continuous variables. Bivariate analysis and multivariable logistic regression analysis was performed to test for determinants of acceptance of COVID-19 vaccination.

Results:
A total of 576 HCWs have responded to the survey with mean age of 35 years. The majority were females (53.3%), medical doctors (55.4%), and located in the capital state, Khartoum (76.0%). The absolute refusal of COVID-19 vaccine was expressed by 16% of the respondents while 57% were willing to get vaccinated. Males were twice to four times more likely to accept the vaccine. Lower acceptability of COVID-19 vaccine was significantly associated with the nursing profession (OR= 0.35, 95% CI:0.15-0.82, P<0.00), increased perceived harm from the vaccine (OR= 0.11, 95% CI: 0.05-0.23, P <0.00), lack of confidence in the source of vaccine (OR= 0.16, 95% CI:0.08-0.31, P=0.00), organizations and government supervising COVID-19 vaccination process (OR=0.31, 95% CI:0.17-0.58 P=0.00).

Conclusions:
This study highlights a moderate level of COVID-19 vaccine acceptability by the HCWs in Sudan. Effective communication of correct, regular, up-to-date evidence on the safety and effectiveness of vaccines is crucial to building trust in vaccines. Special consideration should be in place to address vaccine hesitancy among female HCWs and the nursing profession.

Keywords:
COVID-19; vaccine acceptability; Healthcare workers; Sudan

17 - Abstracts (Day 4, November 17th, 2021 Session 3)

COVID-19

Abstract Code: 2021-EOH-828
Presenter Name: Dr. Areej Shoubaki
Country: Jordan
Presenter Email: ajshowbaky@gmail.com
Authors: Dr. Areej Shoubaki, Dr. Ashraf Aqel, Dr. Ibrahim Ablan, Dr. Mahmoud Yacoub, Dr. Majed Asad, Mr. Mohammad M. Asad , Dr. Mohammad Alhawarat

Title: Reported Adverse Events Following Pfizer-BioNTech Covid-19 Vaccine in Jordan

Background:
The expediency in producing and approving vaccines for the COVID-19 pandemic is unprecedented. Regrettably, this fomented public distrust and misinformation about vaccination. The Pfizer-BioNTech mRNA-based vaccine was the first to be approved for mass use after phase 3 clinical trials affirmed its safety and efficacy. Vaccine safety monitoring is ongoing.

Purpose:
To describe reported adverse events following immunization with the Pfizer-BioNTech vaccine among first recipients in Jordan.

Methodology:
A retrospective descriptive study was conducted on data extracted from the Ministry of Health's database for the National Vaccine Adverse Event Reporting System from January to May 2021. Frequency of adverse events was compared between the first and second doses and across age groups, gender, and comorbidities. Chi-square test was used to compare categorical variables.

Results:
In total, 1,874 individuals who received both doses of the vaccine were studied. The mean age was 68 years with a 2:1 male to female ratio and 73% with comorbidities. About one third (32%) of recipients reported side effects after the first dose, 45.7% after the second, and 24.4% after both. Most common side effects were pain at injection site (32%), fatigue (16%), and headache (8%), followed by fever, myalgia, and arthralgia. Overall reported side effects increased 1.4 times after the second dose mostly for systemic side effects. Significantly more females reported side effects than males, while those with comorbidities reported comparatively less side effects (52.2%) than the medically free (59%). Further, 72.5% of recipients 55 years old and younger reported side effects compared to 50% of older recipients. No anaphylactic reactions were reported. Severe side effects were rare and self-limiting, including tachycardia (n=4), shortness of breath (n=28), lower limb paraesthesia (n=6).

Conclusions:
The Pfizer-BioNTech vaccine has proven to be safe and well-tolerated among vulnerable recipients with comorbidities. Continuous adverse event surveillance and follow-up is recommended.

Keywords:
COVID-19, Jordan, Adverse events, side effects, vaccine

17 - Abstracts (Day 4, November 17th, 2021 Session 3)

COVID-19

Abstract Code: 2021-EOH-717
Presenter Name: Falah Abdul Kader
Country: Iraq
Presenter Email: Falaah.Abd1300a@comed.uobaghdad.edu.iq
Author: Dr. Falah Abdul Kader

Title: Estimation the Out-of-pocket Expenditure on COVID-19 Treatment Among Patients Managed at Home, Iraq, 2020

Background:
The socio-economic impact of the COVID-19 has a devastating effect on health programs, health insurance, and healthcare systems. Also, the losses of jobs and rising prices that have accompanied the pandemic are causing growing poverty. Due to the exhausted Iraqi healthcare system and the lack of trust in its efficiency, many COVID-19 patients are choosing to be treated at home which exerts even more financial pressure on Iraqi families.

Purpose:
To estimate the out-of-pocket expenditure spent on the management of COVID-19 patients exclusively treated at home, Iraq, 2020.

Methodology:
This is a cross-sectional study that involved patients who were diagnosed by the physician as a COVID-19 based on clinical manifestations, Real-time PCR, CT of the lung, and who were exclusively managed at home. A snowball sampling technique was used to enroll COVID-19 patients during November and December 2020. A questionnaire was developed to collect data on basic demographics, clinical manifestation, severity, disease duration, and the estimated total expenditure on consultation fees, radiological and laboratory investigations, medicines, disinfectants, personal protective equipment, oxygen device, and other direct costs.

Results:
The total number of participants was 589; 328 (55.7%) were women. The average total expenditures calculated in Iraqi Dinars was 643,304 (range: 505,096-5,595,000). The highest average expenditure was for medicine cost (191,138), followed by the consultation fees (117,951), CT scan (102,084) laboratory investigations (70,000), medical devices (58,285), personal protective equipment (50,388), chest x-ray (mean=39,437), and disinfectants (37,849). The average expenditure was significantly higher among governmental employees ($P=0.024$), married patients ($P<0.001$), and patients who reported inadequate monthly income ($P<0.001$). Patients' gender, residence, and having comorbidities did not affect the average expenditure ($P>0.05$).

Conclusions:
The management of COVID-19 patients at home imposed a significant financial burden on them and the government should consider measures that mitigate this burden like financial support.

Keywords:
Out-of-pocket expenditure, COVID-19, Iraq, Impact

18 - Abstracts (Day 5, November 18th, 2021 Session 1)

Surveillance

Abstract Code: 2021-HIV/STI-662
Presenter Name: Salwa Al-Eryani
Country: Yemen
Presenter Email: salwaalaryani@gmail.com
Authors: Dr. Ghamdan Altahish, Dr. Labibah Saeed

Title: Evaluation of Dengue Surveillance System, Yemen, Hodeiadah, 2021

Background:
The number of dengue cases reported to World Health Organization (WHO) increased over 8 fold over the last two decades, from 2.4 million in 2010 to 4.2 million in 2019. In Yemen, from January to December 2019, 59,486 suspected dengue cases and 219 deaths with a case fatality rate (CFR) 0.4% were reported. The dengue surveillance system (DSS) provides necessary information for outbreak response. As there was an increase in the number of dengue outbreaks especially in Hodeida last year, therefore it's worthy to evaluate the DSS between January and March 2021.

Purpose:
To assess its usefulness, performance& attributes of the system and identify its strengths and weaknesses.

Methodology:
We used the Centers for Disease Control and Prevention (CDC) Updated Guidelines for Evaluation of Surveillance systems. For data collection, desk review and interviews with stakeholders at a central level were conducted; semi-structured questionnaires were distributed for the sentinel site's coordinators. In addition, registers review was used.
Indicators were developed to evaluate the usefulness and the eight attributes, including: flexibility, stability, simplicity, acceptability, sensitivity. Score percent was calculated and interpreted as poor <60%, average: 60 - <80%, and good: ≥ 80%

Results:
the DSS found to be useful (i.e. using the data for detecting changes in trends of morbidity and mortality). Regarding system attributes: flexibility (22.7%), stability (33.3%), sensitivity (76%), and data quality (31%) were poor, while simplicity (79%), acceptability (76%), and representativeness (65%) were average. The overall DSS performance was poor (47%).

Conclusions:
The DSS was useful. Although acceptability and representativeness were average, flexibility stability, sensitivity, and data quality were poor. Strengthening the DSS through providing basic infrastructure, ensuring sustainability, improving supplement, supervising laboratory testing for dengue fever, and expanding DSS coverage to include private health care facilities are necessary. For data quality, supervision and training is recommended.

Keywords:
Dengue surveillance system evaluation, CDC guidelines, YFETP.

18 - Abstracts (Day 5, November 18th, 2021 Session 1)

Surveillance

Abstract Code: 2021-HIV/STI-663
Presenter Name: Samar Nasher
Country: Yemen
Presenter Email: drsammas8@gmail.com
Authors: Dr. Abdulqawi Qassera, Dr. Abdulwakeel Alsamey, Dr. Rima Alusfi, Dr. Yasser Ghaleb

Title: Electronic Integrated Disease Early Warning System Surveillance Evaluation in Sana'a Capital, Yemen 2021: Surveillance Evaluation

Background:
electronic Integrated Disease Early Warning System (eIDEWS) is an essential system, which provides a better prevention and management of epidemics. Through the data collection, countries are able to determine the priorities to develop suitable interventions that save communities lives. Regardless of conflict in Yemen, eIDEWS is still functioning and expanding to be the effective epidemiological surveillance program.

Purpose:
To determine the usefulness of eIDEWS, assess its performance, and identify the strengths and weaknesses of its implementation.

Methodology:
The usefulness and performance attributes of eIDEWS was evaluated using CDC's updated guidelines for evaluating public health surveillance system. The evaluation was carried out in Sana'a capital from January to March, 2021, by interviewing 25 stakeholders in three levels: central, governorate, and health district, using a semi-structured questionnaire. Attributes of system were ranked as poor (<60), average (60- <80), good (80- <90) and excellent (≥90) on the basis of indicators to calculate the final scores.

Results:
The eIDEWS overall usefulness and performance score was (90%) with an excellent rank. The mean of system attributes was 100% for acceptability, completeness, and timeliness. The flexibility was good (83%) since the change in reporting method is applied difficultly. The system is depending completely on foreign fund, thus system's stability was average (75%). However, eIDEWS was expanded recently to add new health facilities, the representativeness was average (76%).

Conclusions:
The system is working effectively at evaluated sites. The overall system performance was excellent, however, flexibility and stability were good due to the negative adaptation of the system with the reporting method and the absence of other fund resources. Therefore, evaluating the new upgraded system, strengthening the stability by finding other supporting resources, further expanding coverage to include all public and private healthcare facilities are recommended.

Keywords:
Field Epidemiology Training Program, eIDEWS, surveillance, evaluation, Yemen

18 - Abstracts (Day 5, November 18th, 2021 Session 1)

Surveillance

Abstract Code: 2021-HIV/STI-730
Presenter Name: Sara Saeed
Country: Pakistan
Presenter Email: sarasaeeddr@gmail.com
Authors: Dr. Ambreen Chaudhry, Dr. Amjad Mahmood, Dr. Fawad Khalid, Dr. Wasif Malik

Title: Evaluation of Dengue Surveillance System in Islamabad - 2019

Background:
Dengue is a major public health threat since 2005 in Pakistan. Because of their rapid expansion and long duration, dengue epidemics reduce the productive capacity and economic development of many sections of society. Evaluation is very important step of planning cycle to improve the utilization of resources.

Purpose:
The overall objective of the study was to assess how quickly the system can detect epidemics and to measure the capacity of the system to monitor trends of its geographical distribution over time.

Methodology:
A cross sectional study was conducted from July to September 2019 in Islamabad. Quantitative and qualitative assessment of system attributes was carried out according to Updated CDC Guidelines for evaluating public health Surveillance Systems, 2001. Stakeholders were identified and approached. 4 different types of semi structured questionnaires were prepared for each level of stakeholders.

Results:
Simplicity is very good; case definition is uniform and easily understandable. Flexibility is poor, the system is not capable of incorporating changes. Timeliness is excellent in terms of case reporting as well as case response by relevant stakeholders. Data entry operators were less in number but expert in their work, however, quality of data remained a challenge as 40 % forms were deficient in demographic and clinical information. Acceptability by the workers as well as population is very good. Sensitivity is high 87 %. PVP is excellent 76%. Stability is good in terms of finances and logistics. Whereas representativeness is insufficient only 30%.

Conclusions:
The overall performance of the surveillance system for dengue in Islamabad was excellent in terms of sensitivity and PVP. Timeliness is excellent and acceptability is very good. Whereas representativeness is poor. Coverage of the system needs to be extended and private set ups and laboratories to be included. Feedback being an important aspect of planning cycle need improvement.

Keywords:
Surveillance, PVP, Sensitivity, Specificity

18 - Abstracts (Day 5, November 18th, 2021 Session 1)

Surveillance

Abstract Code: 2021-HIV/STI-745
Presenter Name: Dr. Suhaib Abu Failat
Country: Jordan
Presenter Email: s.abufailat@gmail.com
Authors: Dr. Suhaib Abu Failat, Dr. Ashraf Aqel, Dr. Ibrahim Iblan, Dr. Majed Asad, Dr. Nabil Qasem

Title: Evaluation of the Acute Flaccid Paralysis Surveillance System During 2016 - 2020: A Retrospective Study

Background:
Acute flaccid paralysis surveillance played a major role in the global eradication of polio. The World Health Organization adopted this method to monitor the progress towards poliomyelitis eradication. The Expanded Program of Immunization in Jordan has routinely collected acute flaccid paralysis data since 1999, which then attained a polio-free certification. Yet, because of wars in neighboring countries, such as Syria and Iraq, there is a risk of polio outbreaks to occur.

Purpose:
To evaluate the Acute flaccid paralysis surveillance system in Jordan from 2016 to 2020 and identify areas for improvement.

Methodology:
This was a retrospective descriptive study that used data from the Acute flaccid paralysis surveillance system in Jordan between January of 2016 and December of 2020. The World Health Organization standard indicators were used to evaluate the performance of the surveillance system.

Results:
A total of 483 cases of non-polio acute flaccid paralysis were reported. Most of them (n= 478 or 99%) were below 15 years of age, and among those, 55.6% were below 5 years old, and 58% were male. At the national level, the surveillance achieved all the World Health Organization indicators throughout the evaluating period, except for two indicators: the proportion of stool specimens from which non-polio enterovirus was isolated in 2016 and 2017 and the non-polio acute flaccid paralysis detection rate per 100,000 of the population under 15 years of age in 2020. At a sub-national level (governorates level), the proportion of stool specimens from which non-polio enterovirus was isolated, as an indicator, was not achieved most of the time. This was frequently observed in each of all evaluation years of 2016 to 2020. Moreover, most indicators were not achieved at the governorate level in 2020.

Conclusions:
There are some gaps that need improvement in the Acute flaccid paralysis surveillance system in Jordan, especially at the governorate level. The lower performance during 2020 could be referred to the COVID-19 crisis and the lockdown during the pandemic. Similar challenges are possible in the future and proper preparation is required.

Keywords:
Acute Flaccid Paralysis, Jordan, Surveillance system, Polio, Eradication.

18 - Abstracts (Day 5, November 18th, 2021 Session 1)

Surveillance

Abstract Code: 2021-HIV/STI-639
Presenter Name: Sumia AlTurki
Country: Yemen
Presenter Email: sumia.alturki@yahoo.com
Authors: Dr. Abdulfattah Al-Mahdi, Dr. Nosiba Al-Sharafy, Mr. Yasser Ghaleb

Title: Evaluation of the Nutrition Surveillance System, Sana'a city - Yemen 2021

Background:
Malnutrition remains one of the most common causes of morbidity and mortality among children in low- and middle-income countries. It is one of the important problems that showed an increasing incidence in Yemen. The Nutrition Surveillance System (NSS) started in 2018 as a pilot in five governorates to ensure that difficulties of public health importance are monitored efficiently.

Purpose:
To assess its usefulness, and performance of the system attributes, and identify strengths and weaknesses to make recommendations for improvement.

Methodology:
CDC updated guidelines of the evaluation of public health surveillance were used to evaluate the NSS in Sana'a city. Qualitative and quantitative attributes were measured through desk review and in-depth interviews with stakeholders from different levels by used semi-structured questionnaire for collected data. The percent mean of total scores was used for the final rank of the performance as: very poor (<40%), poor (40%<60%), average (60%<80%), good (80%<90%) and excellent (≥90%). The Epi info version 7.2 was used for data entry and analysis.

Results:
The NSS was found to be useful and flexible with overall score 100% and 80%, respectively, and overall system performance was average 76%. The highest attribute score was 83% for simplicity and the lowest score was 67% for stability. Although simplicity and acceptability in the governorate and district levels were good but in health facilities level were average. Timeliness of report, completeness of forms and data were 100 and 95% respectively. The main NSS strength was continuous expansion in opening new health facilities, and quality of data was strong with updated databases.

Conclusions:
NSS in Sana'a city was found to be useful and met its main objective. Overall levels of system performance were average. Regular training for health staff at the health facilities and gradual replacement of donor's with government's funds are recommended.

Keywords:
Evaluation, Nutrition Surveillance System, Yemen, CDC guideline

Seventh EMPHNET Regional Conference
Amman, Jordan

18 - Abstracts (Day 5, November 18th, 2021 Session 1)

Surveillance

Abstract Code: 2021-EOH-738
Presenter Name: Taghreed Hawsawi
Country: Saudi Arabia
Presenter Email: daybyday3030@hotmail.com
Authors: Dr. Shady Kamel, Dr. Tahani Hawsawi

Title: Outbreak Investigation of the Middle East Respiratory Syndrome in Khames Mushait Saudi Arabia, January 2020

Background:
Middle East Respiratory Syndrome (MERS) is a fatal viral respiratory illness. More than half of the laboratory-confirmed MERS-CoV infections with a human-to-human transmission reported globally are healthcare-associated settings. On February 4, 2020, the Ministry of Health (MOH) and the Field Epidemiology Training Program Team investigated a cluster of 6 confirmed MERS cases reported by Asir Health Affairs in a private hospital in Khames Mushait-city, Saudi Arabia.

Purpose:
To identify the source, mood of transmission, epidemiologic links between cases, epidemiologic and clinical features, and contacts tracing of the cases.

Methodology:
A descriptive study was conducted by reviewing the medical records and the MOH investigation forms of the laboratory-confirmed MERS cases and contacts reported between January 19 to January 29, 2020.

Results:
A cluster of 6 confirmed cases was reported. Its source was unknown. There were 3 (50%) health care workers, 3 (50%) Saudis, and 4 (75%) females. Four (75%) had chronic diseases. The mean age was 49.4 (range 26-65) years. One death was reported with a fatality rate of (16%). Two hundred twenty-six healthcare workers (HCWs) and 85 community contacts were traced.

Conclusions:
The investigation team suggested that the source might be an asymptomatic patient from outside the hospital. Thus, it is highly likely that the weak adherence of HCWs to infection prevention and control (IPC) measures led to infection transmission to other HCWs and patients with no community transmission. As a result, cases with comorbidities suffered critical complications. Therefore, the hospital should commit to applying the MOH guidelines to detect and manage MERS cases and IPC measures.

Keywords:
Middle East respiratory syndrome, coronavirus, MERS-CoV, epidemiology, outbreak, Healthcare-associated outbreaks.

19 - Abstracts (Day 5, November 18th, 2021 Session 2)

Others

Abstract Code: 2021-FWBD-854
Presenter Name: Abdullahel Maruf Faruque
Country: Bangladesh
Presenter Email: serenity1509@gmail.com
Authors: Dr. Mahbubur Rahman, Dr. Mallick Masum Billah, Dr. Md. Omar Qayum, Dr. Meerjady Sabrina Flora, Dr. Mohammad Ferdous Rahman Sarker

Title: Health-risk Behaviors in School Going Adolescents in a Rural Subdistrict, Bangladesh, 2019

Background:
Adolescence is the usual time period of life for developing health-risk behaviors which contribute to significant mortality and morbidity in future life. Health-risk behaviors in rural adolescents are commonly overlooked. Since its inception in Dhaka in 1951, the School Health Program in Bangladesh gradually expanded and included behavior change communication training for school teachers . Being implemented in schools of 21 districts out of 64 districts two subdistricts, most of the sub-districts like Belabo are not under the coverage for health-risk behavior prevention programs for adolescents.

Purpose:
We aimed to determine the proportion of existing health-risk behaviors in school going adolescents.

Methodology:
We conducted a cross-sectional study among 9th grade students of 28 high schools at Belabo subdistrict, Narsingdi, Bangladesh from July 20 to August 5, 2019. After taking proper consent, we used a self-administered questionnaire for data collection. We performed a descriptive analysis to find out the proportion of adolescents with different health-risk behaviors.

Results:
Out of 2,132 school going adolescent students (64%) were female. The median age was 15 years. Within one-week prior data collection, 1,710 (81%) students consumed fast foods at least once, 20% students reported that they used smartphones, computers or similar devices for ≥1 hour per day. About 4% students were current smokers, 65% students felt lonely in last one year and 6% students reported of having suicidal thoughts. Among the respondents, 29% males and 19% females reported that they were physically attacked in last one year. About 8% of students reported that they were bullied in last one month.

Conclusions:
The study showed that a relatively high proportion of rural school going adolescent students were at risk of developing health risk behaviors. We recommend to extend school health programs at subdistrict level for prevention of health-risk behaviors in school going adolescents.

Keywords:
Health-risk behavior, Adolescent, Rural, Bangladesh

19 - Abstracts (Day 5, November 18th, 2021 Session 2)

Others

Abstract Code: 2021-OTH-693
Presenter Name: Ahmad Mokharshum
Country: Saudi Arabia
Presenter Email: mokharshum212@gmail.com
Author: Dr. Ahmad Mokharshum

Title: Gastrointestinal Illness Outbreak Among Female Residents at Alahsa Rehabilitation Center, Saudi Arabia, August 2021

Background:
The Rehabilitation Center in Al-Ahsa is affiliated with the Ministry of Human Resources and Social Development. The center houses males and females with disabilities. Between July 20 and August 5, a total of 27 cases of gastrointestinal illness were identified in the rehabilitation facility among female residents.

Purpose:
Describe a Gastrointestinal Illness Outbreak Among female residents at Alahsa rehabilitation center and its causes and measurements taken to prevent such outbreak.

Methodology:
The FETP team visited the kitchen, checked the temperatures on food refrigerators, and Observed how food is prepared and distributed to the units. Female team members interviewed all-female caretakers for patients to identify any dietary preferences and record information on feeding/nutritional habits. In addition, food and water samples, stool specimens from symptomatic cases, and anal swabs from food handlers were obtained.

Results:
27 cases of gastrointestinal illness were identified in the rehabilitation facility. Of those, 12 (44%) cases were admitted to the hospital. 2 (7%) cases died. The cases ranged in age between 21-59 years (median: 40 years). The most reported symptoms were diarrhea (81%) and fever (63%). No cases occurred among patients in the male section nor unit 5 in the female section. They consumed a blended diet that consisted of regular meals except for dairy products. The refrigerator for the dairy products had temperatures between 15-21 °c in the female section. Stool analysis, and culture were negative for both cases and food handlers. Food and water samples were also negative.

Conclusions:
No policies to detect outbreaks were available. Notification, monitoring, and storage systems were defective. Based on this public health investigation, we think that the most likely cause of this outbreak was dairy products, and the etiological agent was listeria Monocytogenes.

Keywords:
gastrointestinal illness, rehabilitation center, alahasa, listeria Monocytogen

19 - Abstracts (Day 5, November 18th, 2021 Session 2)

Others

Abstract Code: 2021-FWBD-874
Presenter Name: Ajmal Zahed
Country: Afghanistan
Presenter Email: Ajmalzahed7@gmail.com
Authors: Dr. Khawja Mir Islam Saeed, Dr. Mohammad Hafiz Rasooly, Ms. Fatema Hassanzada, Ms. Khatera Sufi

Title: Risk Factors of Kidney Stone Among Women in Nimroz Province, Afghanistan in 2021: A Case-control Study

Background:
Kidney stones affect about 12% of the world population at some stage in their lifetime. They have been associated with an increased risk of chronic kidney diseases, end-stage renal failure, cardiovascular diseases, diabetes, and hypertension. No official data is available about the prevalence of kidney stones in Afghanistan. Anecdotal data shows that there is an increasing pattern in the incidence of kidney stones in Nimroze province Afghanistan.

Purpose:
To determine the risk factors associated with a kidney stone among women in Nimroze province, Afghanistan.

Methodology:
This was a case-control study among women in Red Cresent Hospital (RCH) in Nimroze province during June-August 2021. Case was defined as any female with kidney stone newly diagnosis by ultrasonography, and the control was any female without kidney stone attended OPD of the hospital. The nonprobability purposive sampling technique was used. A face-to-face interview was carried out using a structured questionnaire. Bivariate analysis using Chi-square was performed to calculate Odds Ratio and 95% Confidence Interval (95%CI).

Results:
We were able to interview 184 cases and 179 control. The median age among cases was 31 years (Ranged 18-70) and in control 35 years (Ranged 18-61). The study revealed a statistically significant association between kidney stones and family history (OR 6.7, CI 4.2-10.8), daily drinking water up to two glasses (OR 4.4, CI 2.8-6.9), water taste (OR 3, CI 1.9-5.0). sedentary work >1hour per day (OR=2.6, CI=1.7-3.9), high blood pressure (OR=2.2, CI=1.2-3.9), and being obese (OR=1.9, CI=1.08-3.4). Physical exercise and dietary habits were not associated with the kidney stones.

Conclusions:
The risk of kidney stones was found high among those with positive family history, less drinking water, poor quality of water, and sedentary work. Families and the community should be educated about the modifiable risk factors of kidney stones.

Keywords:
Kidney, stones, risk, factors, women.

Seventh EMPHNET Regional Conference
Amman, Jordan

19 - Abstracts (Day 5, November 18th, 2021 Session 2)

Others

Abstract Code: 2021-OTH-685
Presenter Name: Eva Inam Al Zein
Country: Jordan
Presenter Email: eva.alzain@jfda.jo
Authors: Dr. Hala Khazally, Mr. Mamoun Sayaheen

Title: Consumers' Food Safety Knowledge and Practices During COVID-19; A Story from Jordan

Background:
In Western countries, several studies have reported changes in consumers' behaviors regarding food safety in response to the COVID-19 pandemic. The shared responsibility of food safety, between governments, food businesses, and consumers, has been well recognized and calls for extra preventive measures and recommendations be introduced for food handlers, producers, and consumers. Little, however, is known about food safety in developing countries, including Jordan.

Purpose:
This study assessed food safety knowledge and behaviors during the COVID-19 pandemic era in Jordan.

Methodology:
A web-based, self-administered, questionnaire, published via the Jordan Food and Drug Administration website and social media platforms (March-July 2021), was utilized. The questionnaire assessed COVID-19 preventive measures (11 questions), food safety knowledge, (13 questions), and food safety practices (12 questions). Descriptive statistics were presented.

Results:
Out of the total 969 valid responses, 60.7% were females, 33.5% were 38 to 49 years old, 64.8% were married, 64.3% had college or bachelors' degrees, 38.8% were from the central region, 83.5% were living in urban settings, and 32.3% had a medical background. Mean (SD) food safety knowledge score was 8.34 (2.33) (range: 0 -13). Mean food safety knowledge scores were significantly different (P<0.05) by age, marital status, education, field of study, training in food safety, employment status, monthly family income, and area of residence but not by gender and number of family members. Statistically significant correlations between mean knowledge scores and COVID-19 practices scores (p=<0.001, r=0.183) and food safety practices score (p=< 0.001, r = 0.346) were detected.

Conclusions:
Consumers in Jordan seem to have adequate food safety knowledge, and COVID-19-related preventive measures, which may transfer to better food practices and prevention of diseases such as foodborne and COVID-19. Further studies and interventions in this regard are needed in Jordan.

Keywords:
Food Safety, COVID-19, Jordan, Consumer

19 - Abstracts (Day 5, November 18th, 2021 Session 2)

Others

Abstract Code: 2021-OTH-804
Presenter Name: Mansour Al - Taj
Country: Yemen
Presenter Email: mansouraltaj@yahoo.com
Author: Prof. Abdulwahed Al serouri

Title: Impact of Multiple Hygienic Interventions on Caregivers' Behaviors in a Conflict Setting, Yemen: A Cluster-randomized Controlled Trail

Background:
Several household hygiene programs have been implemented by the Ministry of Public Health and Population and International Non-Governmental Organizations to reduce the risk factors related to child morbidity and mortality in Yemen. However, no research was conducted to assess the impact of such interventions on caregiver's hygiene behavior. We therefore carried out a cluster-randomized controlled trail to assess whether such interventions could improve caregiver's hygiene behavior.

Purpose:
To identify the impact of hygiene promotion interventions on mothers' practices on water, sanitation and hygiene

Methodology:
A six-month cluster-randomized control trial was conducted in the Hufash district of the Al-Mahweet Province in Yemen from May to October 2015. Twenty villages were randomly selected and assigned to an intervention arm that received hygiene promotional interventions and control arm. In total, 358 households were interviewed at the baseline and endpoint. A logistic regression model was fitted to data and Adjusted Odds Ratio (AOR) was used to estimate the effect size of the intervention.

Results:
The intervention made significant improvement in caregivers washing hand after using latrine (AOR 2.6, 95% CI 1.75 - 3.90) and before feeding the baby (AOR 1.8, 95% CI 1.14-2.92), safe dispose of child faces (AOR 2.0, 95% CI 1.35-2.53), covering the remaining food (AOR 1.1, 95% CI 1.08-1.19), cleaning the cooking utensils (AOR 1.27, 95% CI 1.08-1.51) and the cleanness of drinking water storage container (AOR 1.3, 95% CI 1.17-1.46). However, the intervention had no effect on caregiver's handwashing practices after cleaning child faces, before preparing food and before eating meal as well as no improvement in cleanness of the floor of kitchen.

Conclusions:
The findings from this trial reveal the important role that hygiene promotion can play in improving caregivers' behaviors that could lead to better child health at high-risk communities where access to primary health care is limited.

Keywords:
Hygiene, Caregiver, Intervention and Yemen

19 - Abstracts (Day 5, November 18th, 2021 Session 2)

Others

Abstract Code: 2021-OTH-845
Presenter Name: Lauren Miller
Country: Other
Presenter Email: lm1427@georgetown.edu
Authors: Dr. Claire Standley, Dr. Erin Sorrell, Mr. Alexander Linder

Title: Advancing Laboratory Biorisk Assessments and Implementing Validated Waste Management Systems in Libya's Public Health and Veterinary Laboratories

Background:
Implementing a comprehensive biorisk management system is critical to reduce both the safety and security risks associated with biological agents. In order to promote biorisk management best practices at the institutional level, laboratories need to undergo biorisk assessments to identify biosafety and biosecurity gaps.

Purpose:
A collaboration between the Libyan National Centre for Disease Control, the National Centre for Animal Health, Georgetown University and Jordan University of Science and Technology, resulted in an online training curriculum on the basics of biosafety and

Methodology:
Pre-training surveys were used to establish a baseline understanding of existing laboratory infrastructure, biorisk management systems and waste management practices in participating laboratories. Using these findings, we developed a training curriculum to address knowledge gaps, case study scenarios for demonstration, and a comprehensive tool for conducting biorisk assessments. Additional emphasis was placed on correctional waste management to demonstrate how biorisk assessments can inform the implementation of best practices and mitigate risks.

Results:
Results from the pre-training surveys highlighted a significant number of gaps pertaining to basic laboratory functions, biosafety and biosecurity capacity, as well as waste management capacity. Following the completion of the online training, participants will have the opportunity to pilot the lab assessment tool in their laboratory facility. The tool has been designed to automatically create an Action Plan highlighting top priority gaps to address.

Conclusions:
Libya faces perennial challenges to sustaining health security capabilities. Our aim is to support the systematic analysis of public health and veterinary laboratory structures and capabilities to provide insight on gaps specific to biosafety and waste management and support laboratory officials with the technical knowledge to validate waste management practices across national and provincial laboratories.

Keywords:
Libya, laboratory, biorisk management, biorisk assessment, waste management, biosafety, biosecurity

20 - Abstracts (Day 5, November 18th, 2021 Session 3)

Others

Abstract Code: 2021-OTH-849
Presenter Name: Kashif Hussain Qureshi
Country: Pakistan
Presenter Email: kashifqureshidoc@gmail.com
Authors: Mr. Mohammad Ali, Mr. Mudassar Hussain, Mr. Naveed Masood, Mr. Saeed Ahmed Bhurt

Title: Outbreak Investigation of Foodborne Illness in Village Bachal Soomro, District Tharparkar Sindh Pakistan; December 2020; A Retrospective Cohort Study

Background:
On 3rd December 2020, District health authorities reported 15 suspected cases of gastroenteritis including 4 deaths from village Bachal Soomro after attending mourning ceremony.

Purpose:
FELTP Fellows investigated the suspected outbreak with the objectives to determine magnitude of outbreak and to evaluate associated risk factors.

Methodology:
A retrospective cohort study was conducted on 3rd December at village Bachal Soomro, a complete cohort was traced back and a pretested structured questionnaire was adopted to get demographic, clinical, and risk factor information. A case was defined as "any individual who attended mourning ceremony at Village Bachal-Soomro on 3rd December 2020 presented with any of the symptom i.e., loose Motions, abdominal pain/cramps & drowsiness". Descriptive analysis followed by multiple logistic regression was done. Different blood, stool, oropharyngeal swabs, drinking water and milk samples were sent for microbiological and chemical investigation.

Results:
Out of 61 Participants, median age was 23 years (range 1-70 years) with 52% were male. The overall attack rate was 72% whilst the most affected age group was 10-19 years with an attack rate of 85%. Majority of the cases presented with loose motion (77%) followed by abdominal Pain (72%) vomiting (70%), body ache (48%), Drowsiness (16%) and fever (7%). Among all food items dessert (Kheerani) had highest food specific attack rate of 90%. Among all risk factors, consumption of dessert (Kheerani) (AOR: 61.3; CI=6.1-613.1), water (AOR: 23.9; CI=2.0-276.7) and Buffalo Milk (AOR: 7.9; 95% C-I=1.22-51.8) were found significantly associated. Distribution of cases shows common point source that probably due to a single source of pathogen.

Conclusions:
Dessert was prepared 10 to 12 hours prior to meal intake so outbreak was probably caused by dessert (kheerani) prepared by contaminated milk and water, with enterotoxins of Staphylococcus aureus. Community awareness on personal hygiene, proper storage/preservation of food items and provision of safe drinking water suggested.

Keywords:
Foodborne illness, Outbreak Investigation, Retrospective Cohort Study.

Seventh EMPHNET Regional Conference
Amman, Jordan

20 - Abstracts (Day 5, November 18th, 2021 Session 3)

Others

Abstract Code: 2021-EOH-823
Presenter Name: Mohammad Towhidul Anwar
Country: Bangladesh
Presenter Email: towhid45cmc@gmail.com
Authors: Dr. Farzana Islam Khan, Dr. Mahbubur Rahman, Dr. Mallick Masum Billah, Dr. Zakir Hossain Habib, Dr. Tahmina Shirin

Title: Diarrhea Outbreak for Using Impurified Canal Water in Barguna, A Southern Coastal District of Bangladesh, March 2021

Background:
In Bangladesh, people of coastal region uses canal water for drinking and daily activities during pre-monsoon seasons (March-May) as groundwater sources become unavailable. On March 15, 2021, Institute of Epidemiology, Disease Control and Research (IEDCR) was notified an outbreak of acute watery diarrhoea in Barguna district, Bangladesh.

Purpose:
We investigated the outbreak to identify sources and risk factors.

Methodology:
We conducted a case-control study (1:2 ratio) from March 16 – 23, 2021. Cases included any person, aged ≥ 5 years with passage of ≥ 3 loose watery stools / 24 hours living in Barguna district. Cases were enrolled from admitted diarrhea patients in district hospital and controls were enrolled from community. Bivariate and multivariate logistic regression was performed to calculate Odds Ratio (OR), adjusted Odds Ratio (aOR) and 95% Confidence Interval (95%CI). Stool and water samples were tested and environmental assessment was done.

Results:
Among 30 cases and 60 controls, 55% (49/90) were female. Eating panta bhat (leftover cooked rice fermented in water) prepared by impurified canal water (OR 13.5, 95%CI 3.9-49.2) and using utensils washed in canal water (OR 4.0, 95% CI 1.5-10.8) showed higher risk for diarrhoea. However, in multivariate analysis, eating panta bhat prepared by impurified canal water (AOR 10.8, 95%CI 3.2-36.2), had a strong association with diarrhoea after adjusting effect of using canal water for washing utensils. The canal water was turbid, stagnant and steady. Local people had inadequate knowledge and resource for purification of canal water. About 17% (5/30) stool samples collected from cases were positive for Vibrio cholerae in rapid test (Cholkit) and 100% (7/7) water samples collected from canals had >50 faecal coliforms/100 ml.

Conclusions:
Eating panta bhat prepared by impurified canal water was most likely associated with the outbreak. We recommended health authorities to supply water purification tablets for purification of canal water before use.

Keywords:
Impurified canal water, Panta bhat, Acute watery diarrhoea, Coastal district

20 - Abstracts (Day 5, November 18th, 2021 Session 3)

Others

Abstract Code: 2021-OTH-701
Presenter Name: Mohammed Al Amad
Country: Yemen
Presenter Email: alemadm69@gmail.com
Authors: Dr. Intesar Al Shargabi, Dr. Riham Al-Dubaiee, Dr. Sami Al-Haidari, Dr. Suaad Moghalles

Title: Bacterial Contamination of Intensive Care Units, Sana'a City, 2019, Yemen

Background:
Bacterial contamination of intensive care unit (ICU) is one of the risk factors responsible for high incidence of nosocomial infection that can significantly increase the mortality among ICU patients.

Purpose:
To investigate the bacterial contamination and pathogen isolates from ICU environment of Sana'a city hospitals.

Methodology:
A descriptive cross-sectional study in Sana'a city hospitals during 5th to 15th December 2019 was conducted. All hospitals that frequently reported mortality among ICU patients were included. Sterile swab moistened in sterile normal saline were used for collecting samples. Seven ICU sites including; patient's bed, bedside table, masks of O2 supplying apparatus, intravenous stand (IV stand), knop door, wall and floor were targeted and two samples from each site were collected. The samples were transported to National Central Public Health Laboratory for microbiological culture.

Results:
A total of 112 swabs were collected from ICU of eight hospitals. 87 (78%) yielded positive bacterial growth and 109 bacterial strains including 62% (68) gram-positive and 38% (41) gram-negative bacteria were isolated. Coagulase-negative staphylococcus, Staphylococcus aureus, and Bacillus cerus were predominate gram positive which accounted for 28% (30), 21% (23) and 10% (11) of bacterial isolates, respectively. Klebsiella species, Pseudomonas species and Acinetobacter were the gram-negative isolates accounted for 13% (14), 13% (14) and 12% (13) of all bacterial isolates, respectively. The common contaminated sites were patient's beds/bedside tables (40 strains, 37%), floors (24 strains, 22%), walls (15 strains, 14%) and masks of O2 supplying apparatus (12 strains, 11%). Knop door and IV stand were contaminated by (9 strains, 8%) of isolated bacteria.

Conclusions:
The contamination of ICU environment was high and patient's surroundings were the most contaminated. Implementations of strict quality standards of hygienic manners and effective cleaning of inanimate surfaces by hospital's infection control units and periodic monitoring by health authority are highly recommended.

Keywords:
Bacterial Contamination, Intensive Care Units, Yemen

Seventh EMPHNET Regional Conference
Amman, Jordan

20 - Abstracts (Day 5, November 18th, 2021 Session 3)

Others

Abstract Code: 2021-HIV/STI-821
Presenter Name: Rasha AlSiddig
Country: Qatar
Presenter Email: ralsiddig@hamad.qa
Authors: Dr. Elmoubashar Abd Farag, Dr. Redhwan Mohamed, Prof. Abdul Badi Abou-Samra

Title: Challenges of Health-related Research in Qatar from Perspective of Principal Investigators (2021)

Background:
Investing in health-related research is critical for improving life quality. Recent studies have endeavored to address the challenges facing the clinical research environment. However, currently there is no research which has investigated challenges of conducting research from the perspective of the principal investigators (PIs) in Qatar.

Purpose:
To study the relation between strategic challenges facing PIs during the implementation of their research project and research type.

Methodology:
An anonymous, questionnaire was employed. The survey have targeted the PIs who are conducting health-related research in Qatar. The data collected from 102 PIs and Correlation between challenges and research types have been studied using ANOVA test while the likelihood of challenges and research types has been analyzed using Odds ratio.

Results:
There is a significant correlation between financial challenges with basic research, and clinical research with P-value of (0.001) and (0.004) respectively. While HR challenges correlated with basic science research and epidemiological research with P-value of (0.05) and (0.02) respectively. Laboratory work challenges demonstrate significant correlation with basic science research, and clinical research science with P-value of (0.05) and (0.04). It has been found that the probability of experiencing financial challenges has increased significantly with clinical research and basic science research about 6 times (Odd ratio= 5.7 & 5.9 respectively), and 3 times with observational research studies (Odd ratio=3.1). While probability of HR challenges increase significantly 2 times with basic research science, and 4 times with epidemiological study with odd ratio of 2.88 and 3.1 respectively. The probability of Laboratory work challenges increases significantly about 4 times with basic research science and 6 times with clinical research study (odd ratio of 3.67 and 5.73 respectively).

Conclusions:
The association between the study type and challenges was defined. The probability of facing financial, HR, and laboratory work challenges have increased with basic science, clinical, and epidemiological research studies.

Keywords:
Principal Investigators (PIs), financial challenges, Human Resources (HR)

20 - Abstracts (Day 5, November 18th, 2021 Session 3)

Others

Abstract Code: 2021-EOH-658
Presenter Name: Reem Abu Shomar
Country: Palestine
Presenter Email: reemabushomar@gmail.com
Author: Dr. Reem Abu Shomar

Title: Anti-Microbial Resistant Bacteria in Health Care Facilities: Exploring Links with WASH – Gaza, Palestine, 2021

Background:
Antimicrobial resistance (AMR) is a growing global phenomenon however, its link to Water, Sanitation and Hygiene (WASH) remains underexplored, particularly in healthcare facilities (HCFs) where humanitarian crises prevail.

Purpose:
The study aimed to identify AMR bacteria in samples collected from WASH services in two hospitals in Gaza, and to investigate the presence of AMR genes.

Methodology:
A hospital-based cross-sectional study to detect and identify antimicrobial resistance bacteria was conducted. Random samples from water, wastewater, soap, and surface swabs (n=345) were collected from Al-Shifa and European Gaza hospitals. The Samples was screened for the presence of Enterobacteriaceae, Pseudomonas, Enterococcus, and Staphylococcus aureus. Antimicrobial susceptibility, ESBL production, Carbapenem resistance, and AMR genes were investigated.

Results:
High levels of bacterial contamination was detected in water and surface swab samples with an overall percentage 34.1%. Of the total positive microbial growth, (35.7%) on m-Endo Agar, (25.2%) on Mannitol Salt Agar, (23.8%) on Cetrimide Agar; and (12.2%) on m-Enterococcus Agar. Twenty-two percent of identified Enterobacteriaceae was positive for ESBL and (14%) was positive for Modified Hodge test (MHT). Over 2/3 of isolated Enterobacteriaceae in water and wastewater samples found resistant to Amikacin, Ceftazidime, Ceftriaxone, and Imipenem. All Enterobacteriaceae isolates from swab samples were found to be resistant to Pipracillin-Tazopactam, Amikacin, Ceftazidime, and Ceftriaxone. The prevalence of ESBL genes among Enterobacteriaceae isolates were: 25% OXA, 19.4% SHV, 2.8% KPC, 66.7% TEM, 41.7% blaCTXM, and 5.6% blaCTXM-3. For Carbapenem resistant gene (MDM), the prevalence among Enterobacteriaceae was 11.1% and among Pseudomonas was 12.5%. The antibiotic susceptibility profile was also presented for Pseudomonas, Enterococcus and S. aureus.

Conclusions:
The results underline the level of contamination with AMR bacteria in WASH samples and highlights the need to consider the safety of WASH service at HCFs as an essential aspect to fight against the spread of AMR and interrupt nosocomial transmission.

Keywords:
Antimicrobial resistant bacteria, Antimicrobial resistance genes, Healthcare Facilities, Wastewater, WASH, Water.

20 - Abstracts (Day 5, November 18th, 2021 Session 3)

Others

Abstract Code: 2021-OTH-843
Presenter Name: Tarek Alsanouri
Country: GHD
Presenter Email: tsanouri@globalhealthdev.org
Authors: Dr. Alexander Linder, Dr. Claire J. Standley, Dr. Erin M. Sorrell, Dr. Lauren Miller, Dr. Omar Elahmer

Title: Strengthening Laboratory Capacity for Sample Management and Inventory: A North Africa Regional Collaboration

Background:
Safe and secure specimen handling is a central aspect of biorisk management for diagnostic and research laboratories, across the human, animal and environmental health sectors. With respect to infectious biological agents, such practices must also take into consideration appropriate inventory and storage where indicated, and waste management systems for specimen elimination. Establishing regional networks for training, implementation, and sharing of expertise can help encourage uptake of these important practices across public and private sector laboratories, and lead to sustainable outcomes.

Purpose:
Building off previous engagements in the North Africa region, Global Health Development will collaborate with Georgetown University to develop and strengthen a network of laboratories across Morocco, Tunisia and Libya with the intent to enhance safe and secure speciment management practices with emphasis on inventory and waste management.

Methodology:
Taking a One Health approach, the project will create a network of initially ten laboratories from across the three countries, and conduct stakeholder interviews, site assessments, and other information-gathering to identify needs, priorities, and potential contextually-appropriate solutions for enhancing sample inventory, consolidation, and safe elimination in North Africa. Through training and virtual engagement, participants will have the opportunity to share experiences, learn new approaches, and contribute to building their professional networks.

Results:
Results from preliminary needs assessments have revealed gaps with respect to understanding and practices related to sample inventory, handling, and elimination. Although the project is in its early stages, preliminary reactions from potential participating laboratories has been very positive.

Conclusions:
Although further evaluation will be required to determine the short- and longer-term impact of the project, we believe this collaboration represents a promising opportunity for sustainable and impactful laboratory biorisk management capacity strengthening in a key field relevant for pandemic preparedness and response, and moreover demonstrates an effective partnership between local, regional, and international institutions.

Keywords:
laboratory networks; biorisk management; specimen inventory; waste management; One Health

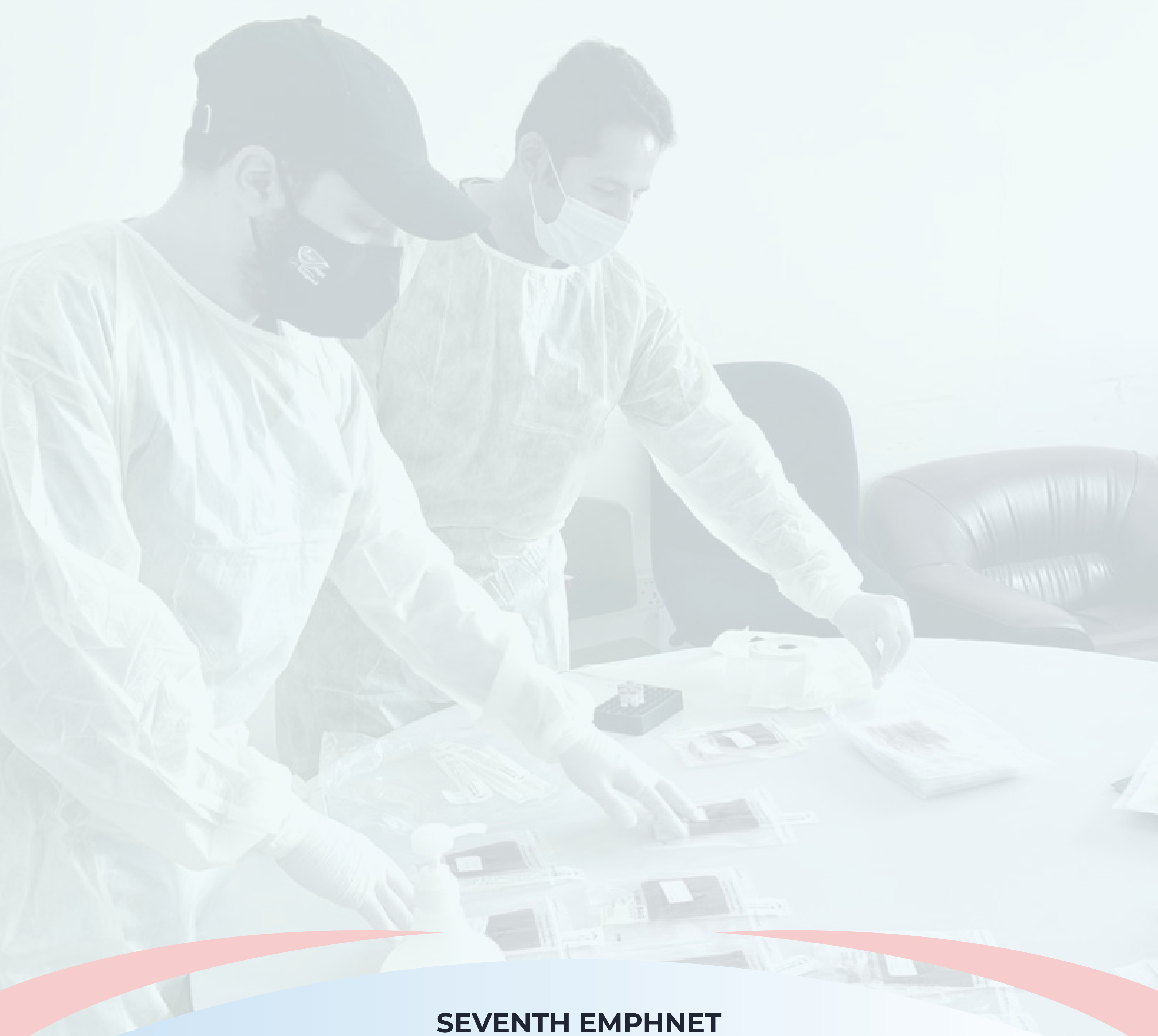

SEVENTH EMPHNET REGIONAL CONFERENCE

November 14-18, 2021

10 years

Seventh EMPHNET Regional Conference

Towards Public Health Resilience in the EMR: Breaking Barriers

EMPHNET

The Eastern Mediterranean Public Health Network
